# Synthetic Approaches to a Challenging and Unusual Structure—An Amino-Pyrrolidine Guanine Core

**DOI:** 10.3390/molecules25040797

**Published:** 2020-02-12

**Authors:** Rafael Rippel, Luís Pinheiro, Mónica Lopes, Ana Lourenço, Luísa M. Ferreira, Paula S. Branco

**Affiliations:** LAQV-REQUIMTE, NOVA School of Science and Technology, 2829-516 Caparica, Portugal; r.rippel@campus.fct.unl.pt (R.R.); l.pinheiro@campus.fct.unl.pt (L.P.); mam.lopes@campus.fct.unl.pt (M.L.); ana.lourenco@fct.unl.pt (A.L.)

**Keywords:** amino acid decarboxylation, cyclic-guanidine, Cernumidine, radical decarboxylation

## Abstract

The synthesis of an unreported 2-aminopyrrolidine-1-carboxamidine unit is here described for the first time. This unusual and promising structure was attained through the oxidative decarboxylation of amino acids using the pair of reagents, silver(I)/peroxydisulfate (Ag(I)/S_2_O_8_^2−^) followed by intermolecular (in the case of l-proline derivative) and intramolecular trapping (in the case of acyl l-arginine) by *N*-nucleophiles. The l-proline approach has a broader scope for the synthesis of 2-aminopyrrolidine-1-carboxamidine derivatives, whereas the intramolecular cyclization afforded by the l-acylarginines, when applied, results in higher yields. The former allowed the first synthesis of cernumidine, a natural alkaloid isolated in 2011 from *Solanum cernuum* Vell, as its racemic form.

## 1. Introduction

**Figure 1:**
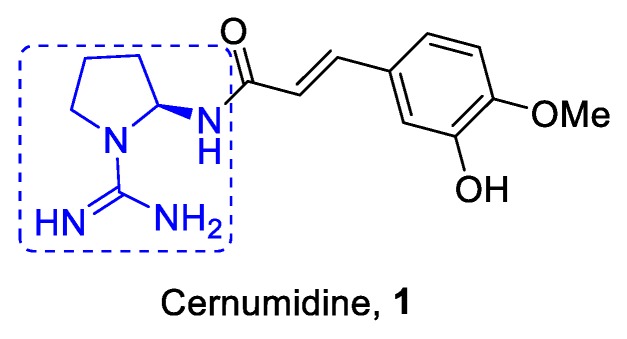
Structure of cernumidine (**1**); in marked blue is the 2-aminopyrrolidine-1-carboxamidine unit presenting the cyclic-guanidine core.

Small molecules are desirable for the pharmaceutical industry and medicinal chemistry where the search for “hits” with specific effects on cells are essential. The drug discovery community surely knows the classes of molecules associated with increased clinical success [1,2], and a large portion of the approved drugs are small molecules containing nitrogen-based aromatic heterocycles such as pyrazoles, imidazoles, benzoimidazoles, triazoles, and 2-amine-pyrimidine moieties [3]. Being aware of the dimension of the drug-like chemical space able to accommodate an enormous diversity of small molecules [4], chemical biology researchers started to look at fragment-based drug discovery as a different approach [5]. Without detracting from chemical synthesis, nature is still the best source of diversity, but problems arise when more material is needed to pursue applicability studies. Chemical modifications to improve therapeutic action, complete synthesis, and semisynthesis contribute significantly to this purpose [6,7]. In their 2016 review paper, Newman and Cragg make a detailed analysis of the relevance of natural products in the origin of new drugs from 1984 to 2014 [8]. About one-third of all approved drugs classified as “small molecules” in this period are natural products, derived from natural or synthetic products but with a natural pharmacophore. Guanidine derivatives can be found in several natural products from diverse origin [9,10] and this structural unit is present as a building block in various pharmaceuticals such as rosuvastatin a cholesterol biosynthesis inhibitor, [11] guanabenz used clinically as an antihypertensive agent, [12] imatinib a tyrosine kinase inhibitor [13] used as an anticancer drug and the blockbuster drug used to treat peptic ulcers and cimetidine a histamine H2 receptor antagonist that inhibits stomach acid production [14]. Considered to be one of the strongest organic bases (pK_HA_ = 13.6) guanidine has the capacity to bind to carboxylates, phosphates and metals with the guanidinium cation engaging special interactions between ligand/receptor or enzyme/substrate [15]. Because guanidines are super bases and oxoanion hosts, they are good supporting ligands in organometallic and coordination chemistry, and can also be used as catalysts [16]. Cernumidine (**1**) (Figure 1) is an alkaloid isolated in 2011 [17] presenting an unusual cyclic-guanidine core and displaying inhibition of interleukine-8 production by HT-29 colon carcinoma cells [18,19,20,21]. Cernumidine and other isomeric compounds isolated from the Brazilian native tree used in folk medicine, *Solanum cernuum* Vell., present a never reported 2-aminopyrrolidine-1-carboxamidine unit with five consecutive C-N bonds that, although structurally very simple and with only a labile stereogenic center, presents a major synthetic challenge to a synthetic chemist [22,23].

**Figure 2:**
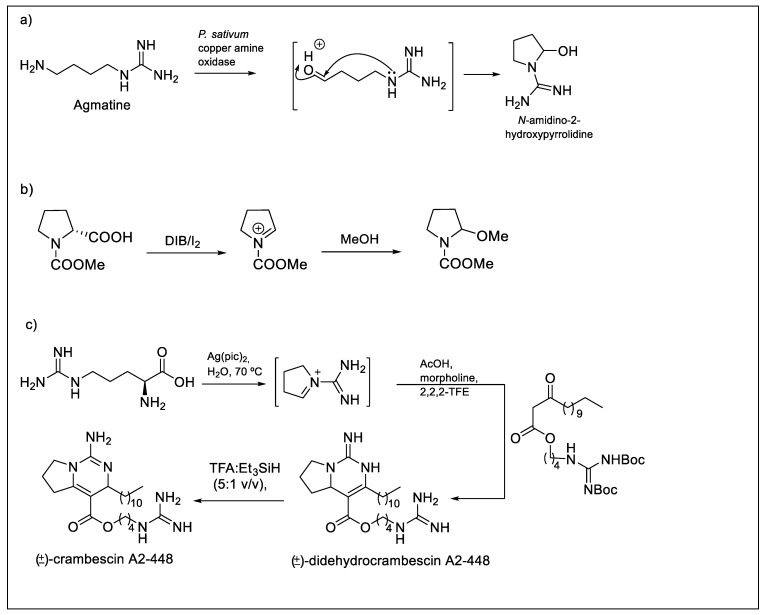
Reported efforts on the synthesis of a cyclic-guanidine core. Works from: (**a**) Ascenzi, P., et al. [24], (**b**) Boto, A., et al. [25,27], and (**c**) Silva et al [29].

Previous efforts on the synthesis of a cyclic-guanidine core were reported in the work of Ascenzi et al. who described the biological synthesis of the hemiaminal, 2-hydroxopyrrolidin-1-yl carboxamidine from agmatine ((4-aminobutyl)guanidine) using copper aminoxidase from *Pisum sativum* L. in an oxidative deamination process. The formation of the cyclic product *N*-amidino-2-hydroxypyrrolidine should occur within the enzyme’s catalytic center since the presence of free 4-guanidobutyraldehyde (shown within square brackets in Figure 2a) was never detected [24]. Although not precisely to attain a cyclic-guanidine core, Boto et al. developed a process aimed at the oxidative decarboxylation of a l-ornithine methyl carbamate derivative using the system (diacetoxyiodo)benzene or iodosylbenzene at room temperature followed by intramolecular cyclization to a 2-aminopyrrolidine nucleus [25]. In this, and other reported works, they showed that the carboxyl radical evolves by loss of carbon dioxide to produce a carbon radical which, in turn, undergoes oxidation to an *N*-acyliminium ion intermediary demonstrated through inter- and intramolecular trapping with oxygen, nitrogen and carbon nucleophiles [26,27,28]. They also showed that the reaction with proline is limited to *N*-protected carbamates (Figure 2b) [27]. In a very recent work, Silva et al. [29] on trying to elucidate the metabolic pathway of crambescins, suggested arginine and fatty acids as precursors for the broad chemical diversity of this family. Their hypothesis was corroborated with a bio-inspired synthesis of crambescin A2-448. Starting from arginine, they postulate that the oxidative decarboxylation generates in situ a highly reactive pyrrolinium salt, and through a Biginelli-like reaction between C-2/C-3 activated fatty acids and a central guanidinylated pyrrolinium on a cascade sequence the bicyclic system of crambescins was obtained in a single operation (Figure 2c). There are several procedures available to effect oxidative decarboxylation of amino acids. Previously we have already mentioned the methodology of Boto et al. involving the use of the pair of oxidizing reagents diacetoxiiodobenzene/I_2_ (DIB/I_2_) [25,26,27,28]. Others, combined this process with a Friedel–Crafts reaction in which they used aromatic rings (benzene, furan, thiophene, among others) as nucleophiles to react with the acyliminium ion formed after the oxidative decarboxylation [30,31]. Also using a hypervalent iodine reagent, the *o*-iodoxybenzoic acid, Akamanchi et al. [32] pursued the decarboxylation of amino acids to nitriles in aqueous ammonia. Other methods for amino acid decarboxylation include the use of hypohalides such as hypochlorite and hypobromite [33], N-bromosuccinimide [34], or the pair of reagents, silver(I)/peroxydisulfate (Ag(I)/S_2_O_8_^2−^) applied for the first time by Stewart et al. [35] for the preparation of aldehydes.

The formation of the metastable ion Ag(II) generated in situ, can be isolated by chelation with nitrogen aromatic ligands such as picolinic acid to form the silver picolinate salt (II), Ag(pic)_2_. The work of Clarke et al. [36] and Silverman and Zelechonok [37] helped to elucidate the mechanism of the oxidative decarboxylation reaction with amino acids. Moreover, the synergistic effect of Cu(II) on this system which can act as a co-catalyst that facilitates the oxidation of the alkyl radical to an alkene, alcohol, aldehydes, ketones or even imides has been addressed [38,39,40]. In addition to silver, the metal catalysed approaches for the decarboxylative coupling of amino acids can also be performed with Fe(I) [41], Co(II) [42], Cu(I) [43,44] Ce(IV) [45]. Although several methodologies have been developed in the last ten years, to the best of our knowledge, the decarboxylation of amino acids with basic residues in the side chain remains elusive. Moreover, the use of nitrogen nucleophiles in metal catalysed decarboxylative coupling was never reported for intermolecular reactions [46].

Here, we report for the first time the synthesis of the cyclic-guanidine core, the 2-aminopyrrolidine-1-carboxamidine unit (Figure 1) that ultimately allows us to synthesize the natural product cernumidine (**1**) and analogues in its racemic form.

## 2. Results and Discussion

**Scheme 1:**
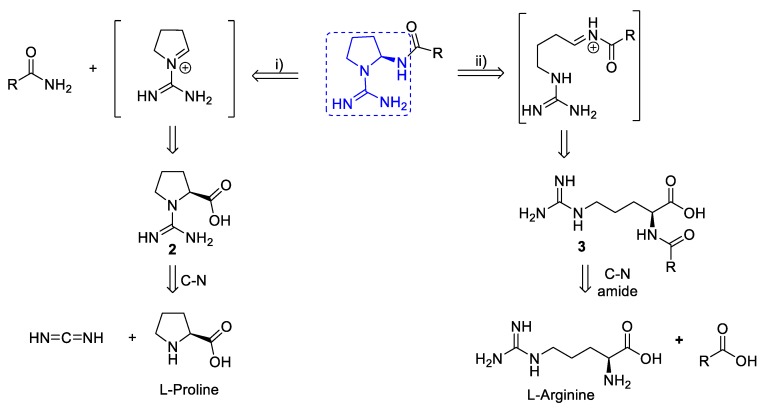
Retrosynthetic approach to the synthesis of 2-aminopyrrolidine-1-carboxamidine derivatives.

Two complementary strategies were applied for the synthesis of 2-aminopyrrolidine-1-carboxamidine derivatives (Scheme 1) starting from l-proline and l-arginine through their guanidine (**2**) and acyl derivatives (**3**), respectively. The delineated synthetic method involves two approaches: (i) oxidative decarboxylation of carbamimidoyl-l-proline (**2**) followed by the intermolecular trapping of the iminium intermediate by nucleophiles and, (ii) oxidative decarboxylation of (**3**) followed by intramolecular trapping of the acyliminium intermediate.

### 2.1. Studies on the Oxidative Decarboxylation of l-arginine Derivatives (***3***)

Several l-arginine derivatives were synthesized as described in Table 1. Following the literature procedure [47] *N*-benzoylarginine (**3a**) was obtained in 47% yield by reaction with benzoyl chloride.

**Scheme 2:**
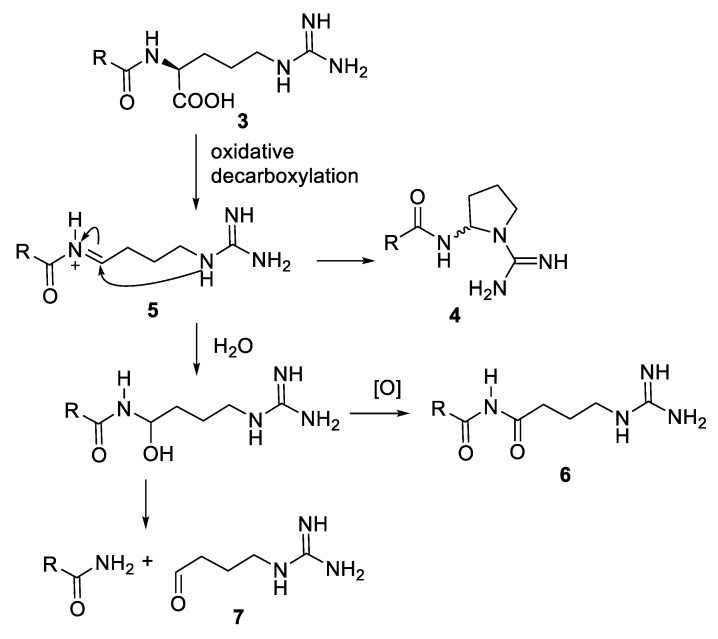
Proposed mechanism for the oxidative decarboxylation of *N*^α^-acyl-l-arginine derivatives (**3**) to 2-aminopyrrolidine-1-carboxamidine derivatives (**4**).

Other acyl chlorides were used when commercially available (as in the case of the synthesis of compound **3g**) or in situ prepared by reaction of the corresponding carboxylic acid with SOCl_2_ (Method A—compounds **3c**, **3e,** and **3f** were thus prepared). Alternatively, compounds **3b**, **3d,** and **3h** were prepared using carbonyldiimidazole (CDI) as coupling reagent (Method B) [48]. Next, we turned our attention to the oxidative decarboxylation of *N*^α^-acyl-l-arginine derivatives (**3**) (Scheme 2). Based on the literature [29], we propose that the oxidative decarboxylation of **3** will generate an iminium intermediate (**5**), which goes through an intramolecular cyclization to **4**. Depending on the reaction conditions, **5** can be trapped by water to the hemiaminal that is further oxidized to the imide (**6**) or cleavage to the aldehyde (**7**) and the corresponding amide.

Screening of the reaction conditions and results are presented in Table 2. The first assays for the oxidative decarboxylation were performed on derivative **3a** using the pair of oxidizing reagents diacetoxyiodobenzene/iodine (DIB/I_2_) [25,49]. After several attempts, when a mixture of acetic acid and DCM was used as solvent (Table 2, entry 7) compound **4a** was obtained in 59% yield, probably due to the higher solubility of **3a** in this solvent mixture.

Next, we decided to turn our attention to the procedure described by Zelechonok [37] and use the pair of reagents AgNO_3_/(NH_4_)_2_S_2_O_8_. These conditions lead to much faster reactions and have the advantage that they can be performed in water, which is very suitable for these substrates. It is worth to mention that since the physical separation of **4a** and **6a** proved to be challenging, from there on whenever the formation of **6a** was observed only relative amounts of each product will be described in Table 2. Using AgNO_3_/(NH_4_)_2_S_2_O_8_ (0.1:1.2) 39% conversion to compound **4a** was observed together with compound **6a** (Table 2, entry 8). The formation of imides in these conditions were already reported by Huang [40] on the oxidative decarboxylation of *N*-acyl amino acids induced by Ag^+^/Cu^2+^/S_2_O_8_^2−^ in water. To obviate the formation of **6a** the reaction was performed in other solvents, such as DMF, AcOH or MeOH but it proved to be slower with the formation of complex mixtures (Table 2, entry 9 to 10) or incomplete when MeOH was used (Table 2, entry 11). When the reaction was performed in AcOH/H_2_O (Table 2, entry 12) in addition to compound **4a** and **6a**, compound **7** was also identified in 26% by NMR (Supplementary Figure S1) [50]. Since the reaction proved to be unsuccessful when performed in organic solvents, we decided to optimize the results attained in entry 8 by increasing the amount of catalyst and oxidant (Table 2, entry 13). Despite of the higher conversion observed, the increased load of persulfate also doubled the conversion to **6a**. Being aware of the synergetic effect of copper in decarboxylation reactions, CuSO_4_ was applied to our system [38,40]. The use of catalytic amounts of CuSO_4_ with an excess of oxidant (3 equiv., Table 2, entry 14) lead to discouraging results. However, with 1 equivalent of CuSO_4_ the conversion of the product increased to 76% (89% based on the recovered starting material, Table 2, entry 15) despite the formation of **6a**. Increasing the temperature to 60 ˚C lead to a complete reaction in 0.5 h as a mixture of compound **4a** and **6a** (Table 2, entry 16). Only by inverting the addition of the reagents and making the arginine derivative react first with CuSO_4,_ the formation of **6a** was suppressed and compound **4a** was isolated in 72% yield (Table 2, entry 17).

The reaction was then extended to compounds **3b**–**3h**. Complex mixtures were obtained with compounds **3c**, **3d**, **3e** and **3f** all bearing moieties able to be involved in radical reactions. Positive results were only achieved for **4b** and **4h** although, in the latter, the reaction yield was low due to incomplete reaction (Table 3). As expected, in this last case it was observed by HPLC-ESI-MS the presence of two peaks, which would correspond to the two diastereomers of **4h** as a result of the racemization at the C-2 position of the pyrrolidine ring being formed (Supplementary Figure S2). However, for our surprise analysis of **3h** also shows two peaks in the HPLC-ESI-MS instead of only one (Supplementary Figure S3). These peaks co-eluted with the two diastereomers of **4h** (Supplementary Figure S2a). This means that racemization occurs not only during the oxidative decarboxylation but also during the formation of the dipeptide.

Next we turned our attention to the alternative strategy involving the oxidative decarboxylation of carbamimidoyl-l-proline (**2**) using the same oxidative system (AgNO_3_/(NH_4_)_2_S_2_O_8_).

### 2.2. Studies on the Oxidative Decarboxylation of Carbamimidoyl-l-proline (***2***)

**Scheme 3:**
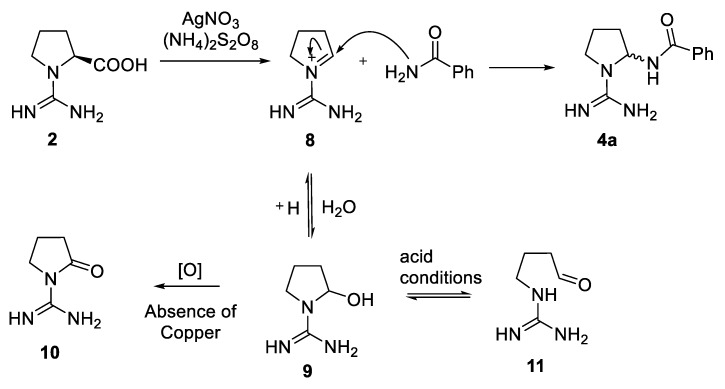
Proposed mechanism for the oxidative decarboxylation of carbamimidoyl-l-proline (**2**) to 2-aminopyrrolidine-1-carboxamidine (**4a**).

We believe that this approach proceeds through a mechanism like the one proposed for the arginine derivatives. As in the latter, the oxidative decarboxylation of **2** generates an iminium ion (**8**) that will be trapped by benzamide to form **4a** (Scheme 3). Here, once again, the reaction conditions will play a crucial role in the reaction outcome. The iminium (**8**) can be trapped by water to the hemiaminal (**9**) that can be further oxidized to the pyrrolidone (**10**) or be cleaved to the aldehyde (**11**). Screening of the reaction conditions is presented in (Table 4). Initially, our attention was devoted to the solubility of benzamide, and therefore the first assays (Table 4, entries 1–6) were carried out in organic solvents, but none proved to be suitable to afford the desired product **4a**. Moreover, in all these assays it was possible to observe in the NMR spectra (not isolated and not quantified) the formation of pyrrolidone (**10**) as the major product and hemiaminal (**9**) as a minor product (Supplementary Figure S4, data in accordance with literature [24]). Next, considering the solubility of **2** we decided to fully adopt the conditions used for the decarboxylation of **3** by using water as solvent and CuSO_4_ as a co-catalyst. In these conditions (Table 4, entries 7–8) the formation of **10** was supressed entirely and for the first time, the formation of the desired product **4a** was observed.

Curiously, the lower reaction time and formation of product (Table 4, entry **8**) contrasts directly with the result observed in entry 7 where only hemiaminal (**9**) was formed during the reaction, which indicates that the temperature is crucial for the reaction outcome. Afterwards, we investigated the role of copper in the formation of **10** (Table 4, entries 9 and 10). In the absence of any nucleophile when the reaction is carried out without copper (Table 4, entry 9) compound **10** was observed as the main product, whereas in the presence of copper the major product was the hemiaminal (**9**) (Table 4, entry **10**), which suggests that copper prevents the oxidation of **9**. Once the formation of **10** was suppressed, we turned our attention to finding strategies that would avoid the formation **9**. We started by changing the reaction solvent to MeOH (Table 4, entry 11) and ACN (Table 4, entry 12) but in the former, the reaction did not occur and in the latter, the yield of **4a** was low. Since the reaction proved to be successful only in water, we investigate if the conversion of the hemiaminal (**9**) into the iminium (**8**) was possible. We anticipated that under acidic conditions it should be possible to favour the formation of **8** from **9**. By adding catalytic amounts of *p*-toluenesulfonic acid (*p*-TsOH) to the reaction (Table 4, entry 13) we only observed the formation of **4a**. However, we were not able to separate the desired product from the *p*-TsOH. Nonetheless, inspired by the previous result, we decided to use an acid resin (Amberlyst 15) that was easily removed from the reaction mixture and allowed the isolation of the desired product in 56% yield (Table 4, entry 14). With these conditions, we were able to observe an inverse correlation between the yield and reaction time. By increasing the reaction time, the reaction yield decreases (Table 4, entries 15–17). Reducing the reaction time (to 6 h) the yield also decreased (Table 4, entry 18) and the product **4a** was obtained in only 18% yield. These findings suggest that the reaction outcome has close relation between the reactivity of the reagents and product stability. That was confirmed by mixing **4a** and Amberlyst 15 in water at 60 °C for 24 h, which results in a 1:1 mixture of hemiaminal (**9**) and **4a**. However, despite the product stability, we believed that other factors might justify the low yield of the reaction. In all the assays that were performed the yield of the reaction contrasts with the information obtained by the NMR analysis of the reaction mixture. The NMR always led us to conceive that we were in the presence of a simple mixture of the desired product and the correspondent benzamide. Thus, knowing already that degradation of product occurs during the reaction, we decided to analyse the decarboxylation step of our reaction. For that purpose, the reaction mixture attained (Table 4, entry 10) was analysed by HPLC-ESI-MS (Supplementary Figure S5). With a retention time of 4.1 min, it was possible to identify compound **9** by the peak at *m*/*z* 130 that correspond to the [M + H]^+^ and the pyrrolidone (**10**) with the peaks at *m*/*z* 128 and *m*/*z* 146 that correspond to the [M + H]^+^ and [M + H+ H_2_O]^+^, respectively. With a retention time of 4.5 min the peaks at *m*/*z* 156 and *m*/*z* 313 in negative mode correspond to the ions [M − H]^-^ and [2M − H]^-^ of the starting material **2** which are in accordance in positive mode with the peaks at *m*/*z* 158 and *m*/*z* 315. The LC-MS indicates the presence of other compounds which shows the lability of the intermediates involved and indicates that, even prior to the formation of our product, the outcome of our reaction is being affected by a variety of divergent pathways. This factor, combined with the instability of the products, may explain the low to moderate yields obtained in this approach.

Afterwards, to examine the scope of this decarboxylating coupling reaction, various amides were reacted under the optimized reaction conditions. Remarkably, at this time we were able to achieve several 2-aminopyrrolidine-1-carboxamidine derivatives (Table 3) as compounds **4c**, **4d**, and **4e** that were not attainable by the method involving the oxidative decarboxylation of the *N*^α^-acyl-l-arginine derivatives **3c**, **3f**, and **3g**. Gladly, although in its racemic form, we were able to synthesize for the first time the cernumidine alkaloid (**1**) in 22% yield from reaction of compound **2** with acetylated isoferulic amide.

To fully explore the scope of the reaction, sulfinate salts were tested as nucleophiles without success. We believe that the sulfinate salts are even more prone to retro-elimination of the nucleophile, and therefore the products were not stable under the acidic conditions of the reaction. The use of other nucleophiles is limited by the stability of the product being formed and the acidic conditions used, which is only compatible with non-basic nucleophiles.

## 3. Materials and Methods

All the reagents and solvents were obtained commercially (Merck KGaA, Darmstadt, Germany), and these were used without further purification. The solvents used were dried using current laboratory techniques. Thin-layer chromatography (TLC) was carried out on aluminium backed Kieselgel 60 F254 silica gel plates (Merck KGaA, Darmstadt, Germany). Plates were visualized under UV light (254 and/or 366 nm). Reversed phase column chromatography was carried out on silica gel LiChoroprep RP-18 (40–63 µm, Merck KGaA, Darmstadt, Germany). Ultraviolet spectroscopy (UV) was recorded on a Thermo Corporation spectrophotometer (Waltham, MA, USA), Helius γ, on quartz cell support. Absorption spectrum measurements were made in the range of 190–320 nm. Infrared spectroscopy (IR) was recorded on a PerkinElmer Spectrum Two (Waltham, MA, USA) in the range of 4000–400 cm^−1^. ^1^H and ^13^C NMR spectra were recorded on a Brucker ARX400 (Billerica, MA, USA) at 400 and 101 MHz, respectively. Chemical shifts are reported in parts per million (ppm, δ units). The following NMR abbreviations are used: s = singlet, d = doublet, t = triplet, q = quartet, m = multiplet, brs = broad singlet. The relative amount of each compound presented in Table 2 was based on the analysis of the crude NMR, where the individualized integral of the H-2 proton of each compound was used. HPLC-ESI-MS was acquired on an Agilent 1200 Series LC (Santa Clara, CA, USA) with a quaternary pump, ALS, TCC, DAD, and Agilent G6130B LC/MSD (Santa Clara, CA, USA) with API-ES source, in FIA mode or LC mode with a Zorbax-SB-C18 (4.6 × 150 mm, 5 µm, Agilent, Santa Clara, CA, USA) (Porto University) and in a QqTOF Impact IITM mass spectrometer (Bruker Daltonics, Billerica, MA, USA) operating in the high resolution mode. Samples were analyzed by flow injection analysis (FIA) using an isocratic gradient 50A:50B of 0.1% formic acid in water (A) and 0.1% of formic acid in acetonitrile (B), at a flow rate of 10 µL·min^−1^ over 15 min. Calibration of the TOF analyzer was performed with a calibrant solution of sodium formate 10 mM. The full scan mass spectra were acquired over a mass range of 50–1000 *m*/*z*, at a spectra rate of 0.2 Hz. Data was processed using Data Analysis 4.2 software (Lisbon University, Bruker, Billerica, MA, USA, 2015). ESI-HRMS (Electrospray ionization-high resolution mass spectrometry) was performed in an LTQ Orbitrap XLTM mass spectrometer (Thermo Fischer Scientific, Bremen, Germany) controlled by LTQ Tune Plus 2.5.5 and Xcalibur 2.1.0. The capillary voltage of the electrospray ionization (ESI) was set to 3100 V. The capillary temperature was 275 °C. The sheath gas flow rate (nitrogen) was set to 6 (arbitrary unit as provided by the software settings). The capillary voltage was 49 V and the tube lens voltage 130 V. The description of these spectra follows the format: mass (*m/z*); attribution in the molecule. The designation [M + H]^+^ always corresponds to the molecular ion of interest.

### 3.1. General Procedure for the Synthesis of Arginine Derivatives

Method A (**3a**, **3c**, **3e**–**3g**)

To a solution of the corresponding carboxylic acid in toluene (volume to make a 0.5 M solution of substrate), SOCl_2_ (5 equiv.) was slowly added and the reaction was stirred at reflux temperature for 1 h. Then, volatiles were removed under reduced pressure to afford the desired acyl chloride.

To a round-bottom flask charged with a solution of l-Arginine in water (volume to make a 0.2 M solution of substrate) Na_2_CO_3_ (2 equiv.) was added. The solution was cooled at 0 °C and the acyl chloride (1.2 equiv.) was added portion wise. The reaction mixture was stirred at 0 °C for 10 min and then at r.t. until complete consumption of the starting material (24 to 72 h). After that, the mixture was acidified with a solution of HCl 3 M to pH 1, and the aqueous phase was washed 3× with DMC. The resulting aqueous phase was then basified with NaOH 4 M to pH 8, evaporated until dryness and purified by using RP18 column chromatography. The collected fractions were analyzed by UV-Vis and were then combined according to the obtained UV-spectra to afford the desired product.

Method B (**3b**, **3d** and **3h**)

To a solution of the correspondent carboxylic acid (1.2 equiv.) in dry DCM (volume to make a 0.5 M solution of substrate) CDI (1.5 equiv.) was added, and the reaction was stirred until complete consumption of the starting material (TLC, DCM/MeOH 19:1). Then, the reaction mixture was concentrated under vacuum and to the attained residue a solution of the arginine derivative (1 equiv.) in DMF (volume to make a 0.5 M solution of substrate) and DMAP (0.1 equiv.) were added and the reaction was stirred at 80 °C until complete consumption of the starting material (TLC, DCM/MeOH/H_2_O 13:7:1). After the consumption of the starting material, the solvent was removed under reduced pressured and the reaction mixture was purified by RP-18 column chromatography. The collected fractions were analyzed by UV-vis and combined according to the obtained UV-spectra.

*Compound***3a** [51]. Following the general procedure A, from 4 g of l-Arginine compound **3a** was obtained in 47% yield; m.p. 285–288 °C. IV (*ATR*) ν_máx_ (cm^−^^1^) 3200, 1677, 1615, 1592. ^1^H RMN (400 MHz, D_2_O) δ 7.80 (d, *J* = 7.7 Hz, 2H, H-3′ and H-7′), 7.62 (t, *J* = 7.2 Hz, 1H, H-5′), 7.54 (t, *J* = 7.6 Hz, 2H, H-4′ and H-6′), 4.41 (dd, *J* = 8.1, 5.1 Hz, 1H, H-2), 3.23 (t, *J* = 6.8 Hz, 2H, H-5), 2.07–1.92 (m, 1H, H-3a), 1.91–1.77 (m, 1H, H-3b), 1.75–1.63 (m, 2H, H-4). ^13^C NMR (101 MHz, D_2_O) δ 178.22 (C-1), 169.94 (C-1′), 156.62 (C-6), 133.42 (C-2′), 132.06 (C-5′), 128.64 (C-3′ e H-7′), 126.97 (C-4′ e H-6′), 55.04 (C-2), 40.58 (C-5), 28.95 (C-3), 24.50 (C-5).

*Compound***3b**. Following the general procedure B, from 1 g of l-Arginine compound **3b** was obtained in 59% yield. m.p. 90–93 °C. IR (*ATR*) ν_máx_ (cm^−^^1^) 3160, 1604, 1543, 1501, 1393, 1252. ^1^H RMN (400 MHz, D_2_O) δ 7.79 (d, *J* = 8.6 Hz, 2H, H-3′ e H-7′), 7.05 (d, *J* = 8.7 Hz, 2H, H-4′ e H-6′), 4.39 (dd, *J* = 7.8, 5.2 Hz, 1H, H-2), 3.88 (s, 3H, OMe), 3.22 (t, *J* = 6.7 Hz, 2H, H-5), 2.05–1.91 (m, 1H, H-3a), 1.89–1.76 (m, 1H, H-3b), 1.74–1.61 (m, 2H, H-4). ^13^C NMR (101 MHz, D_2_O) δ 178.72 (C-1), 169.30 (C-1′), 161.91 (C-5′), 156.62 (C-6), 129.09 (C-3′ e C-7′), 125.72 (C-2′), 113.88 (C-4′ e C-6′), 55.41 (OMe), 55.14 (C-2), 40.63 (C-5), 28.87 (C-3), 24.55 (C-4). HRMS (ESI) *m*/*z* calculated for C_14_H_20_N_4_O_4_ [MH^+^]: 308.14846; Found: 308.14556.

*Compound***3c** [52]. Following the general procedure **A**, from 2 g of l-Arginine compound **3c** was obtained in 55% yield. m.p. 190–192 °C. IR (*ATR*) ν_máx_ (cm^−^^1^) 3333, 3161, 3046, 2944, 1667, 1567. ^1^H RMN (400 MHz, CD_3_OD) δ 7.54–7.44 (m, 3H, H-3′, H-5′ e H-9′), 7.43–7.31 (m, 3H, H-6′, H-7′ e H-8′), 6.73 (d, *J* = 15.8 Hz, 1H, H-2′), 4.46 (dd, *J* = 7.6, 4.8 Hz, 1H, H-2), 3.23 (tt, *J* = 13.5, 6.8 Hz, 2H, H-5), 2.04–1.91 (m, 1H, H-3a), 1.89–1.76 (m, 1H, H-3b), 1.75–1.62 (m, 2H, H-4). ^13^C NMR (101 MHz, CD_3_OD) δ 177.61 (C-1), 166.61 (C-1′), 157.30 (C-6), 140.14 (C-3′), 134.93 (C-4′), 129.28 (C-7′), 128.45 (C-6′ e C-8′), 127.48 (C-5′ e C-9′), 120.83 (C-2′), 54.29 (C-2), 40.67 (C-5), 29.80 (C-3), 24.86 (C-4).

*Compound***3d**. Following the general procedure B, from g of l-Arginine compound **3d** was obtained in 55% yield. m.p. 112-115 °C. IR (*ATR*) ν_máx_ (cm^−^^1^) 3400, 2900, 1638, 1605, 1573, 1510, 1394, 1251. ^1^H RMN (400 MHz, CD_3_OD) δ 7.38–7.27 (m, 3H, H-3′e, H-5′ e H-9′), 6.80 (d, *J* = 8.5 Hz, 2H, H-6′ e H-8′), 6.48 (d, *J* = 15.7 Hz, 1H, H-2′), 4.36 (dd, *J* = 8.0, 4.8 Hz, 1H, H-2), 3.72 (s, 3H, OMe), 3.20–3.04 (m, 2H, H-5), 1.93–1.79 (m, 1H, H-3a), 1.77–1.64 (m, 1H, H-3b), 1.65–1.53 (m, 2H, H-4). ^13^C NMR (101 MHz, CD_3_OD) δ 179.18 (C-1), 168.46 (C-1′), 162.48 (C-7′), 158.71 (C-6), 141.32 (C-3′), 130.48 (C-5′ e H-9′), 128.91 (C-4′), 119.70 (C-2′), 115.26 (C-6′ e C-8′), 55.83 (OMe), 42.06 (C-5), 31.19 (C-3), 26.32 (C-4). HRMS (ESI) *m*/*z* calculated for C_16_H_22_N_4_O_4_ [MH]^+^: 334.16411; Found: 334.16135.

*Compound***3e**. Following the general procedure A, from 0.5 g of l-Arginine compound **3e** was obtained in 63% yield. m.p. 125–128 °C. IR (*ATR*) ν_máx_ (cm^−^^1^) 3258, 3160, 2969, 2872, 1633, 1557, 1394. ^1^H RMN (400 MHz, D_2_O) δ 7.35 (t, *J* = 7.4 Hz, 2H, H-6′ e H-8′), 7.31–7.22 (m, 3H, H-5′, H-6′ e H-9′), 4.05 (dd, *J* = 8.3, 4.2 Hz, 1H, H-2), 3.04–2.85 (m, 4H, H-5 e H-3′), 2,63 (t, *J* = 6.9 Hz, 2H, H-2′), 1.72–1.59 (m, 1H, H-3a), 1.56–1.43 (m, 1H, H-3b), 1.15 (p, *J* = 7.5 Hz, 2H, H-4). ^13^C NMR (101 MHz, D_2_O) δ 178.55 (C-1), 174.90 (C-1′), 156.50 (C-6), 140.33 (C-4′), 128.58 (C-6′ e C-8′), 128.49 (C-5′ e C-9′), 126.35 (C-7′), 54.32 (C-2), 40.54 (C-5), 37.24 (C-2′), 31.26 (C-3′), 28.69 (C-3), 24.12 (C-4). HRMS (ESI) *m*/*z* calculated for C_15_H_23_N_4_O_3_ [MH]^+^: 307.17647; Found: 307.17718.

*Compound***3f**. Following the general procedure A, from 0.5 g of l-Arginine compound **3f** was obtained in 81% yield. m.p. 138–141 °C. IR (*ATR*) ν_máx_ (cm^−^^1^) 3262, 3172, 3056, 2864, 1634, 1557, 1495, 1394. ^1^H RMN (400 MHz, D_2_O) δ 7.50–7.29 (m, 5H, H-4′, H-5′, H-6′, H-7′ e H-8′), 4.19 (dd, *J* = 8.1, 4.8 Hz, 1H, H-2), 3.68 (d, *J* = 14.4 Hz, 1H, H-2′a), 3.60 (d, *J* = 14.8 Hz, 1H, H-2′b), 3.11 (t, *J* = 6,9 Hz, 2H, H-5), 1.89–1.77 (m, 1H, H-3a), 1.75–1.61 (m, 1H, H-3b), 1.59–1.43 (m, 2H, H-4). ^13^C NMR (101 MHz, D_2_O) δ 178.47 (C-1), 173.97 (C-1′), 156.54 (C-6), 135.15 (C-3′), 129.06 (C-5′ e C-7′), 128.89 (C-4′ e C-8′), 127.25 (C-6′), 54.57 (C-2), 42.43 (C-2′), 40.49 (C-5), 28.77 (C-3), 24.38 (C-4). HRMS (ESI) *m*/*z* calculated for C_14_H_21_N_4_O_3_ [MH]^+^: 293.16082; Found: 293.16134.

*Compound***3g**. Following the general procedure A, from 0.5 g of l-Arginine compound **3g** was obtained in 38% yield. m.p. 108–111 °C. IR (*ATR*) ν_máx_ (cm^−^^1^) 3029, 1719, 1632, 1563, 1474, 1364, 1203. ^1^H RMN (400 MHz, CD_3_OD) δ 7.23 (t, *J* = 7.8 Hz, 2H, H-4′ e H-6′), 7.07 (t, *J* = 7.2 Hz, 1H, H-5′), 6.99 (d, *J* = 7.9 Hz, 2H, H-3′ e H-7′), 4.01–3.90 (m, 1H, H-2), 3.09 (t, *J* = 6.6 Hz, 2H, H-5), 1.91–1.74 (m, 1H, H-3a), 1.74–1.54 (m, 3H, H-3b e H-4). HRMS (ESI) *m*/*z* calculated for C_13_H_19_N_4_O_4_ [MH^+^]: 295.14008; Found: 295.13994.

*Compound***3h**. [53]. Following the general procedure B, from 0.5 g of l-Arginine compound **3h** was obtained in 31% yield. ^1^H RMN (400 MHz, D_2_O) δ 7.73 (s, 1H, H-6′), 6.97 (s, 1H, H-5′), 4.37–4.29 (m, 1H, H-2′), 4.18 (dd, *J* = 12.4, 5.0 Hz, 1H, H-2), 3.27–3.12 (m, 2H, H-5), 3.12–3.02 (m, 1H, H-3′a), 3.00–2.90 (m, 1H, H-3′b), 1.93–1.78 (m, 1H, H-3a), 1.76–1.62 (m, 1H, H-3b), 1.62–1.48 (m, 2H, H-4), 1.38 (s, 9H, H-3′’). HRMS (ESI) *m*/*z* calculated for C_17_H_29_N_7_O_5_ [MH]^+^: 412.2303; Found: 412.2300.

### 3.2. Synthesis of Carbamimidoyl-l-proline (***2***) [54]

To a round-bottom flask charged with a solution l-Proline in a mixture of water (volume to make a 5.7 M solution of substrate) and methanol (volume to make a 4.3 M solution of substrate) a solution of cyanamide (1.5 equiv.) in water (volume to make a 4.8 M solution of substrate) was added and the reaction mixture was stirred at 100 °C for 16 h. After complete consumption of the starting material, the reaction mixture was concentrated under vacuum and washed several times with isopropanol to afford the desired product as a white solid. ^1^H NMR (400 MHz, D_2_O) δ 4.27 (dd, *J* = 8.6, 2.9 Hz, 1H), 3.62–3.53 (m, 1H), 3.48 (dd, *J* = 16.8, 8.7 Hz, 1H), 2.30 (d, *J* = 10.9 Hz, 1H), 2.18 (dd, *J* = 6.5, 3.1 Hz, 1H), 2.10–1.89 (m, 2H). ^13^C NMR (101 MHz, D_2_O) δ 177.77, 154.73, 62.42, 47.82, 31.02, 23.32.

### 3.3. General Procedure for the Preparation of Compounds ***4*** from Decarboxylation of Arginine Derivatives

Following the adapted procedure reported by Huang et al. [40] to a round-bottom flask charged with a solution of derivatized L-arginine in water (volume to make a 0.4 M solution of substrate) at 60 °C, CuSO_4_.5H_2_O (1 equiv.) was added, and the solution was stirred for 10 min. Then, AgNO_3_ (0.15 equiv.) and NH_4_S_2_O_8_ (1.5 equiv.) were added and the reaction was stirred at r.t. for 30 min (the reaction color evolves from blue to a green solution).

After complete consumption of the starting material, the reaction mixture was concentrated under vacuum and purified by RP-18 column chromatography. The collected fractions were analyzed by UV-vis and combined according to the obtained UV-spectra.

*Compound***4a**. Following the general procedure and starting from 50 mg of **3a** compound **4a** was obtained in 72% yield. ^1^H NMR (400 MHz, D_2_O) δ 7.72 (d, *J* = 7.5 Hz, 1H), 7.61 (t, *J* = 7.2 Hz, 1H), 7.50 (t, *J* = 7.5 Hz, 1H), 5.87 (d, *J* = 6.1 Hz, 1H), 3.60 (t, *J* = 7.5 Hz, 1H), 3.43 (m, 1H), 2.39–2.07 (m, 1H). ^13^C NMR (101 MHz, D_2_O) δ 179.30, 171.02, 134.50, 133.14, 129.73, 128.06, 56.12, 41.66, 30.03, 25.58. HRMS (ESI) *m*/*z* calculated C_12_H_17_N_4_O [MH]^+^: 233.13969; Found: 233.13931.

*Compound***4b**. Following the general procedure and starting from 50 mg of **3b** compound **4b** was obtained in 80% yield. ^1^H RMN (400 MHz, D_2_O) δ 7.57 (d, *J* = 8.7 Hz, 2H, H-3′ e H-7′), 6.87 (d, *J* = 8.7 Hz, 2H, H-4′ e H-6′), 5.75–5.64 (m, 1H, H-2), 3.73 (s, 3H, OMe), 3.54–3.44 (m, 1H, H-5a), 3.38–3.28 (m, 1H, H-5b), 2.27–2.09 (m, 2H, H-3), 2.08–1.94 (m, 2H, H-4). ^13^C NMR (101 MHz, CD_3_OD) δ 179.18, 168.46, 162.47, 158.70, 141.31, 132.28, 130.47, 128.91, 122.49, 119.69, 115.25, 114.90, 114.53, 55.82, 42.06, 31.19, 26.32. HRMS (ESI) *m*/*z* calculated C_13_H_19_N_4_O_2_ [MH]^+^: 263.15025; Found: 263.15060.

*Compound***4h**. Following the general procedure and starting from 50 mg of **3h** compound **4h** was obtained in 7% yield ^1^H NMR (400 MHz, D_2_O) δ 8.64 (s, 1H), 7.33 (s, 1H), 5.71 (d, J = 5.7 Hz, 1H), 4.46–4.34 (m, 1H), 3.61–3.48 (m, 1H), 3.46–3.37 (m, 1H), 2.17–2.07 (m, 1H), 1.96–1.92 (m, 2H), 1.80–1.70 (m, 1H), 1.68–1.51 (m, 2H), 1.39 (s, 9H) (observed in the crude mixture). ESI-HPLC-MS, RT 12.6 min, [M + H]^+^ *m/z*: 412.2; [M − H]^+^ *m/z*: 410.2; RT 12.8 min, [M + H]^+^ *m/z*: 412.2; [M − H]^+^ *m/z*: 410.1. HRMS (ESI) *m*/*z* calculated C_16_H_28_N_7_O_3_ [MH]^+^: 366.2248; Found: 366.2245.

### 3.4. General Procedure for the Preparation of Compounds ***4*** from Decarboxylation of Carbamimidoyl-l-proline (***2***)

Following the adapted procedure reported by Huang et al. [40], to a round-bottom flask charged with a solution of derivatized l-Proline in water (volume to make a 0.8 M solution of substrate) at r.t., CuSO_4_·5H_2_O (1 equiv.), AgNO_3_ (0.15 equiv.) and NH_4_S_2_O_8_ (1.5 equiv.) were added and the reaction was stirred for 30 min at r.t. Then, the reaction was heated to 60 °C and Amberlyst 15 (15% weight of substrate) and the corresponding nucleophile (1.1 equiv.) were added and the reaction was stirred for 16–18 h. After that the reaction mixture was diluted with MeOH and filtered under a celite pad (washed with water and MeOH several times). The filtrate was evaporated until dryness and purified by using RP18 column chromatography. The collected fractions were analyzed by UV-vis and combined according to the obtained UV-spectra to afford the desired product.

*Compound***1** [17]. Following the general procedure and starting from 100 mg of **2** and 1.1 equiv. of amide, compound **1** was obtained in 22% yield. It is worth to mention that the acetyl group protecting the ferulic amide was removed during the reaction due to the acidic conditions used. ^1^H NMR (400 MHz, CD_3_OD) δ 7.54 (d, *J* = 15.6 Hz, 1H), 7.11 (d, *J* = 1.8 Hz, 1H), 7.03 (d, *J* = 8.3 Hz, 1H), 6.79 (d, *J* = 8.2 Hz, 1H), 6.47 (d, *J* = 15.6 Hz, 1H), 5.77 (d, *J* = 5.2 Hz, 1H), 3.86 (s, 3H), 3.59–3.52 (m, 1H), 3.42–3.35 (m, 1H), 2.33–2.23 (m, 2H), 2.15–2.08 (m, 1H), 2.04–1.99 (m, 1H). ^13^C NMR (101 MHz, CD_3_OD) δ 169.96, 156.27, 151.32, 149.98, 149.55, 149.26, 144.60, 127.80, 126.30, 123.78, 117.06, 111.75, 64.78, 56.13, 32.48, 24.18, 23.80. ESI-MS [M + H]^+^ *m/z*: 305.1.

*Compound***4a**. Following the general procedure and starting from 100 mg of **2** and 1.1 equiv. of benzamide, compound **4a** was obtained in 56% yield. In accordance with spectra data of compound obtained by method A.

*Compound***4c**. Following the general procedure and starting from 100 mg of **2** and 1.1 equiv. of amide, compound **4c** was obtained in 22% yield. ^1^H NMR (400 MHz, (CD_3_OD) δ 7.62 (d, *J* = 15.8 Hz, 1H), 7.60–7.55 (m, 2H), 7.42–7.33 (m, 3H), 6.71 (d, *J* = 15.7 Hz, 1H), 5.76 (d, *J* = 6.1 Hz, 1H), 3.63–3.53 (m, 1H), 3.44–3.34 (m, 1H), 2.39–2.20 (m, 2H), 2.15–1.98 (m, 2H). ^13^C NMR (101 MHz, CD_3_OD) δ 169.46, 156.78, 144.14, 135.89, 131.33, 130.04, 129.11, 120.39, 65.27, 48.17, 33.41, 23.77. ESI-MS [M + H]^+^ *m/z*: 259.1. HRMS (ESI) *m*/*z* calculated C_14_H_19_N_4_O [MH]^+^: 259.15534; Found: 259,1561.

*Compound***4d**. Following the general procedure and starting from 100 mg of **2** and 1.1 equiv. of amide, compound **4d** was obtained in 32% yield. ^1^H NMR (400 MHz, CD_3_OD) δ 7.28–7.22 (m, 2H), 7.20–7.13 (m, 3H), 5.59 (d, *J* = 5.4 Hz, 1H), 3.43 (dd, *J* = 12.5, 5.3 Hz, 1H), 3.33 (dd, *J* = 9.0, 6.9 Hz, 1H), 2.90 (t, *J* = 7.4 Hz, 2H), 2.53 (t, *J* = 7.4 Hz, 2H), 2.23–2.00 (m, 2H). ^13^C NMR (101 MHz, CD_3_OD) δ 180.48, 175.53, 156.79, 136.18, 130.08, 129.59, 128.06, 65.27, 43.17, 33.28, 24.26, 23.69. ESI-MS [M + H]^+^ *m/z*: 261.1. HRMS (ESI) *m*/*z* calculated C_14_H_21_N_4_O [MH]^+^: 261.17154; Found: 261.1718.

*Compound***4e**. Following the general procedure f and starting from 100 mg of **2** and 1.1 equiv. of amide, compound **4e** was obtained in 17% yield. ^1^H NMR (400 MHz, CD_3_OD) δ 7.31–7.17 (m, *J* = 11.7, 7.6 Hz, 1H), 5.66 (d, *J* = 5.2 Hz, 1H), 3.57–3.51 (m, 3H), 3.41–3.33 (m, 1H), 2.31–2.21 (m, 2H), 2.14–2.07 (m, 1H), 2–1.95 (m, 1H). ^13^C NMR (101 MHz, CD_3_OD) δ 176.66, 156.72, 141.66, 129.49, 127.33, 64.96, 48.00, 38.34, 33.08, 32.56, 23.87, 23.55. HRMS (ESI) *m*/*z* calculated C_13_H_19_N_4_O [MH]^+^: 247.15589; Found: 247.1560.

*Compound***4f**. Following the general procedure and starting from 100 mg of **2** and 1.1 equiv. of amide, compound **4f** was obtained in 51% yield. ^1^H NMR (400 MHz, D_2_O) δ 6.36–6.29 (m, 2H), 5.80 (d, *J* = 2.6 Hz, 1H), 5.79–5.75 (m, 1H), 3.62–3.54 (m, 1H), 3.46–3.38 (m, 1H), 2.33–2.26 (m, 2H), 2.17–2.10 (m, 1H), 2.04–1.98 (m, 1H). ^13^C NMR (101 MHz, D_2_O) δ 167.55, 129.37, 127.98, 63.83, 46.81, 31.93, 22.30. HRMS (ESI) *m*/*z* calculated C_8_H_15_N_4_O [MH]^+^: 183.1240; Found: 183.1236.

*Compound***4g**. Following the general procedure and starting from 100 mg of **2** and 1.1 equiv. of amide, compound **4g** was obtained in 51% yield. ^1^H NMR (400 MHz, D_2_O) δ 5.66 (d, J = 5.6 Hz, 1H), 3.63–352 (m, 1H), 3.44–3.32 (m, 2H), 2.32–2.06 (m, 4H), 2.02 (s, 3H). ^13^C NMR (101 MHz, D_2_O) δ 173.37, 155.36, 63.69, 46.72, 31.84, 22.27, 21.01. HRMS (ESI) *m*/*z* calculated C_7_H_15_N_4_O [MH]^+^: 171.1240; Found: 171.1241.

## 4. Conclusions

The first metal catalysed decarboxylative coupling of amino acids bearing basic residues were here described. In this process, nitrogen nucleophiles were used for the first time for the iminium trapping through intermolecular reactions. Excitingly, through this method, novel approaches to a challenging and unusual amino-pyrrolidin-guaninium core were reported. This privileged structure is an unexplored motif in natural products and very promising for the design of bioactive synthetic derivatives. Moreover, efforts are being devoted to establish an enantioselective approach for the synthesis of compound **4**. Also, the promising result obtained with the dipeptide **3h** where compound **4h** was obtained, definitely makes the preparation of novel peptidomimetics within reach.

## Figures and Tables

**Table 1:** Procedures used via acyl chloride (Method A) or coupling reagent (Method B) for the preparation of *N*^α^-l-arginine acyl derivatives (**3**).

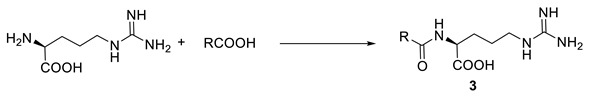
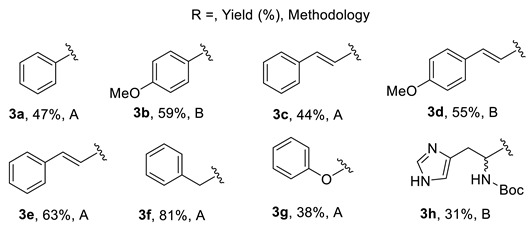

Method A: Acyl chloride (1.1 equiv.), l-arginine (1 equiv.) in water (1.15 M), Na_2_CO_3_ 2 equiv. (pH ≈ 10) at 0 °C then 24 h r.t.; Method B: Carboxylic acid (1,2 equiv.) in dry CH_2_Cl_2_ (0.5 M), CDI (1.5 equiv.) then l-arginine (1 equiv.) 0.5 M in DMF and DMAP (0.1 equiv.). For each compound the synthetic method used is assigned bellow it.

**Table 2:** Screening and results for the oxidative decarboxylation of *N*^α^-benzoyl-l-arginine derivatives (**3a**).

Entry	Oxidant	Additive (equiv.)	Solvent	T (°C)	Time (h)	(3a) Relative Amount (%) ^a^	(4a) Relative Amount (%) ^a^	(6a) Relative Amount (%) ^a^	(7) Relative Amount (%) ^a^
1	DIB/I_2_ (2:0.5)	-	CH_2_Cl_2_	r.t.	48	>90	vestigial	-	-
2 ^f^	DIB/I_2_ (2:0.5)	-	DMF	r.t.	18	100	-	-	-
3	DIB/I_2_ (4:1)	-	CH_3_CN	reflux	56	>90	vestigial	-	-
4 ^f^	DIB/I_2_ (4:1)	-	CH_2_Cl_2_	r.t.	1.5	100	-	-	-
5	DIB/I_2_ (4:1)	-	CH_2_Cl_2_	reflux	48	92	8	-	-
6 ^g^	DIB/I_2_ (4:1)	-	DMSO	r.t.	48	-	-	-	-
7 ^f^	DIB/I_2_ (2:0.5)	-	AcOH/CH_2_Cl_2_ (3:1)	reflux	72	-	59 ^e^	-	-
8 ^b^	Ag(I)/S_2_O_8_^2^^−^ (0.1:1.2)	-	H_2_O	r.t.	3	53	39	8	-
9 ^b,g^	Ag(I)/S_2_O_8_^2^^−^ (0.2:1.2)	-	DMF	r.t.	>24	-	-	-	-
10 ^b,g^	Ag(I)/S_2_O_8_^2^^−^ (0.15:1.2)	-	AcOH	r.t.	96	-	-	-	-
11 ^b,f^	Ag(I)/S_2_O_8_^2^^−^ (0.15:1.5)	-	MeOH	r.t.	48	100	-	-	-
12 ^b^	Ag(I)/S_2_O_8_^2^^−^ (0.15:1.5)	-	AcOH:H_2_O (1:1)	r.t.	20	-	35	39	26
13 ^c^	Ag(I)/S_2_O_8_^2^^−^ (0.15:1.5)	-	H_2_O	r.t.	3	-	69	17	11
14 ^g^	Ag(I)/S_2_O_8_^2^^−^ (0.15:3)	CuSO_4_ (0.2)	H_2_O	r.t.	3	-	-	-	-
15 ^c^	Ag(I)/S_2_O_8_^2^^−^ (0.15:1.5)	CuSO_4_ (1)	H_2_O	r.t.	3	-	89	7	4
16	Ag(I)/S_2_O_8_^2^^−^ (0.15:1.5)	CuSO_4_ (1)	H_2_O	60	0.5	-	83	8	5
17 ^d^	Ag(I)/S_2_O_8_^2^^−^ (0.15:1.5)	CuSO_4_ (1)	H_2_O	60	0.5	-	72 ^e^	-	-

^a^ relative amounts of the compounds based on NMR results of the crude mixture; ^b^ The concentration of **3a** was 0.036 M; ^c^ The concentration of **3a** was 0.072 M; ^d^ Reactions at entries 16 and 17 differ in the order of addition of reagents; ^e^ Pure isolated compound; ^f^ No reaction; ^g^ Complex mixture.

**Table 3:** Scope of substrates for the oxidative decarboxylation of l-arginine derivatives (**3**) and carbamimidoyl-l-proline (**2**).

From l-arginine derivatives (**3**)
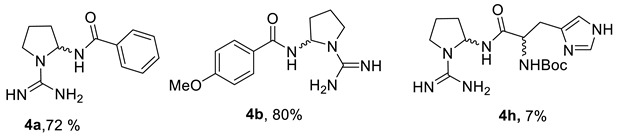
From carbamimidoyl-l-proline (**2**)
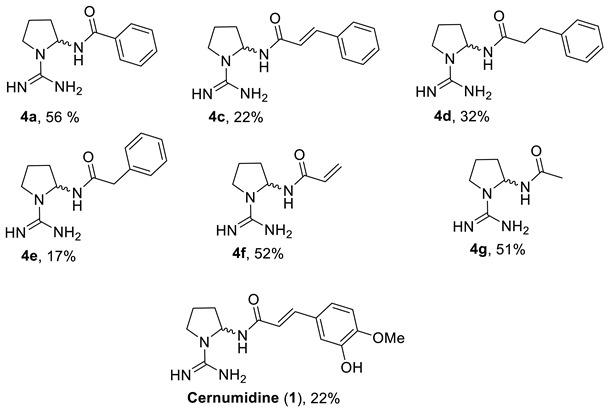

**Table 4:** Screening and results for the oxidative decarboxylation of carbamimidoyl-l-proline (**2**) and trapping the iminium intermediate with benzamide.

Entry	Oxidant	Additive	Solvent	T (°C)	Time (h)	(9) ^a^	(10) ^a^	(4a) Yield (%)
1	Ag(I)/S_2_O_8_^2^^−^ (0.3:3)	-	ACN/H_2_O	r.t.	5.5	✓ ^b^	✓ ^a^	-
2	Ag(I)/S_2_O_8_^2^^−^ (0.3:3)	-	ACN/H_2_O	r.t.	72	✓ ^b^	✓ ^a^	-
3	Ag(I)/S_2_O_8_^2^^−^ (0.3:3)	-	AcOH	r.t.	72	✓ ^b^	✓ ^a^	-
4	Ag(I)/S_2_O_8_^2^^−^ (0.3:3)	-	DMA	r.t.	72	✓ ^b^	✓ ^a^	-
5	Ag(I)/S_2_O_8_^2^^−^ (0.3:3)	-	ACN/H_2_O	80	6	✓ ^b^	✓ ^a^	-
6	Ag(I)/S_2_O_8_^2^^−^ (0.3:3)	-	DMA	r.t.	6	-	-	-
7	Ag(I)/S_2_O_8_^2^^−^ (0.15:1)	CuSO_4_	H_2_O	r.t.	15	✓ ^a^	-	-
8	Ag(I)/S_2_O_8_^2^^−^ (0.15:1)	CuSO_4_	H_2_O	reflux	2	✓ ^a^	-	✓ ^b^
9 ^d^	Ag(I)/S_2_O_8_^2^^−^ (0.15:1)		H_2_O	reflux	2	✓ ^b^	✓ ^a^	-
10 ^d^	Ag(I)/S_2_O_8_^2^^−^ (0.15:1)	CuSO_4_	H_2_O	reflux	2	✓^a^	-	-
11 ^e^	Ag(I)/S_2_O_8_^2^^−^ (0.15:1)	CuSO_4_	MeOH	reflux	16	-	-	-
12	Ag(I)/S_2_O_8_^2^^−^ (0.15:1)	CuSO_4_	ACN	reflux	6	-	✓ ^b^	9
13	Ag(I)/S_2_O_8_^2^^−^ (0.15:1)	CuSO_4_/TsOH (20 mol%)	H_2_O	reflux	16	-	-	✓ ^a^
14	Ag(I)/S_2_O_8_^2^^−^ (0.15:1)	CuSO_4_/Amberlite 15	H_2_O	60 °C	16	-	-	56 ^c^
15	Ag(I)/S_2_O_8_^2^^−^ (0.15:1)	CuSO_4_/Amberlite 15	H_2_O	60 °C	24	-	-	50 ^c^
16	Ag(I)/S_2_O_8_^2^^−^ (0.15:1)	CuSO_4_/Amberlite 15	H_2_O	60 °C	48	-	-	33 ^c^
17	Ag(I)/S_2_O_8_^2^^−^ (0.15:1)	CuSO_4_/Amberlite 15	H_2_O	60 °C	72	-	-	22 ^c^
18	Ag(I)/S_2_O_8_^2^^−^ (0.15:1)	CuSO_4_/Amberlite 15	H_2_O	60 °C	6	-	-	18 ^c^

^a^ Major product; ^b^ Minor product; ^c^ AgNO_3_ (0.15 equiv); CuSO_4_ (1 equiv), (NH_4_)_2_S_2_O_8_ (1 equiv), Amberlite 15 (20% *w*/*w*) in water (1 M) at 60 °C overnight (16–18 h); ^d^ Absence of benzamide; ^e^ No reaction.

## Supplementary Materials

The following are available online. 1H, 13C, DEPT 135 and Mass spectra of compounds **1**, **2**, **3a**–**h** and **4a**–**h** and Figure S1–S4.
