# Supplementary material for: Synthetic Approaches to a Challenging and Unusual Structure—An Amino-Pyrrolidine Guanine Core
Source: Molecules. 2020 Feb 12;25(4):797. doi: 10.3390/molecules25040797 (PMC7070370; doi:10.3390/molecules25040797)
Supplement: Supplementary file 1 [file molecules-25-00797-s001.pdf]

SYNTHETIC APPROACHES TO A CHALLENGING AND UNUSUAL STRUCTURE FROM NATURE - AN AMINO-PYRROLIDINE GUANINE CORE

Rafael Rippel, Luís Pinheiro, Mónica Lopes, Ana Lourenço, Luísa M. Ferreira* and Paula S. Branco*

LAQV-REQUIMTE, NOVA School of Science and Technology, 2829-516 Caparica, Portugal.

Contents

1. NMR and Mass Spectra S2

1.1. ^{1}H, DEPT 135 NMR and ESI-HRMS spectra of L-Arginine derivatives S2

Compound 3a S2

Compound 3b S3

Compound 3c S4

Compound 3d S5

Compound 3e S7

Compound 3f S8

Compound 3g S10

Compound 3h S11

1.2. ^{1}H and ^{13}C NMR spectra of carbamimidoyl-L-proline (2) S11

1.3. ^{1}H, ^{13}C NMR and Mass spectra of compounds 4 and Cernumidine (1) S12

Compound 4a S14

Compound 4b S15

Compound 4c S17

Compound 4d S19

Compound 4e S21

Compound 4f S22

Compound 4g S24

2. Figures S27

1. NMR and Mass Spectra

1.1. ^{1}H, DEPT 135 NMR and ESI-HRMS spectra of L-Arginine derivatives

Compound 3a

^{1}H NMR (D_2O, 400 MHz) spectrum of compound **3a**

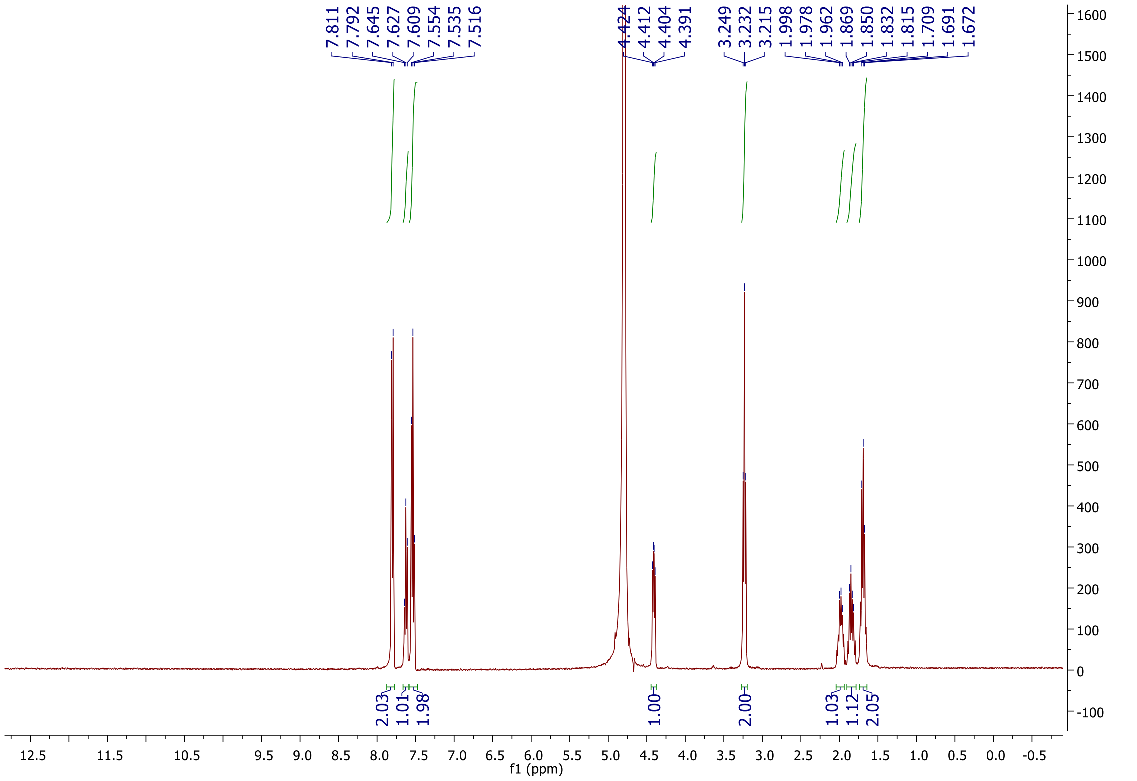

DEPT 135 NMR spectrum of compound **3a**

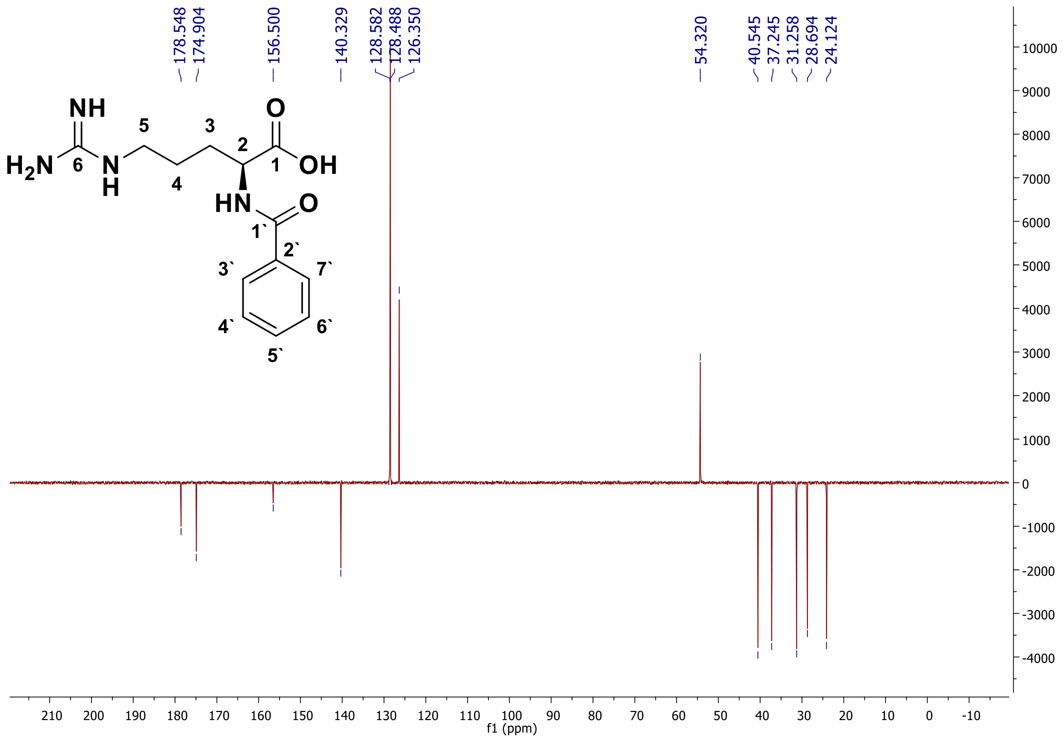

Compound 3b

^{1}H NMR (D_2O, 400 MHz) spectrum of compound **3b**

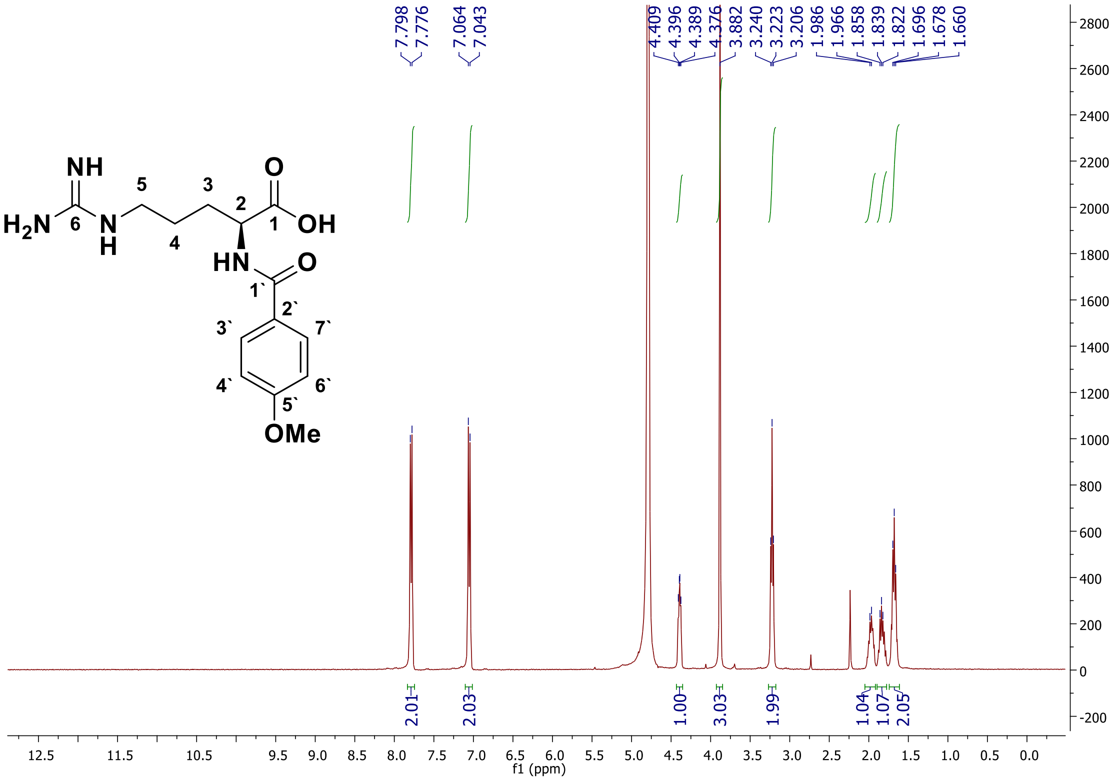

DEPT 135 NMR spectrum of compound **3b**

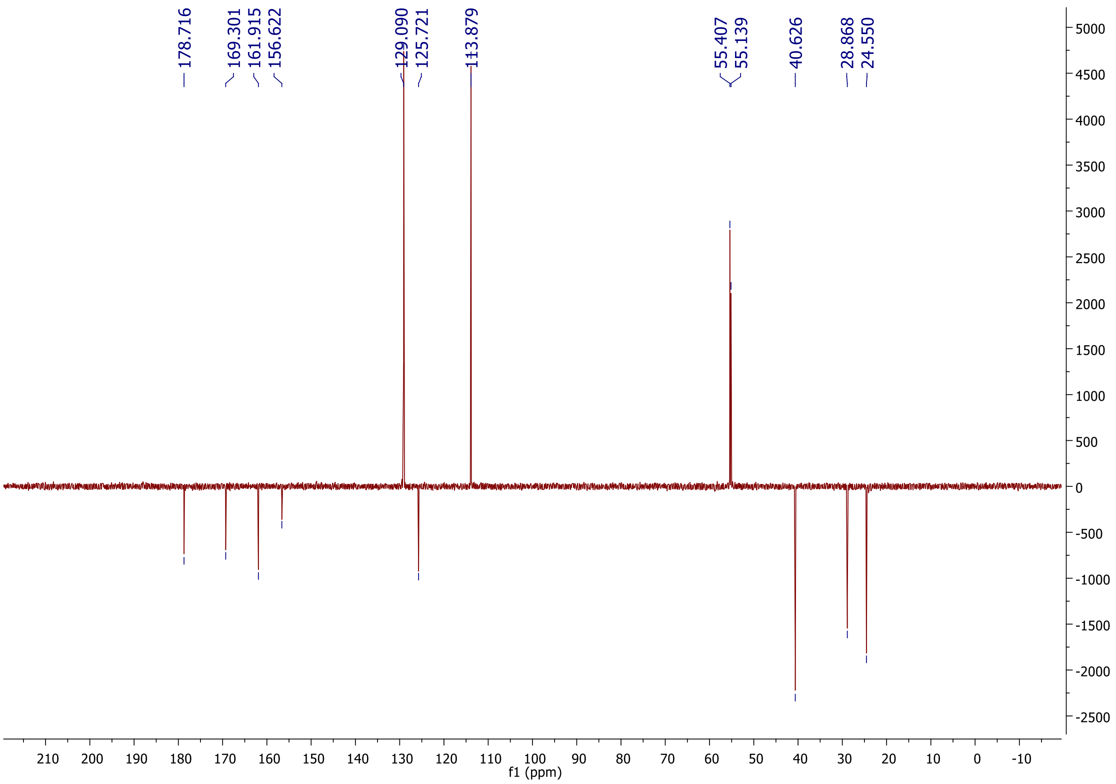

ESI-HRMS(+) of compound **3b**

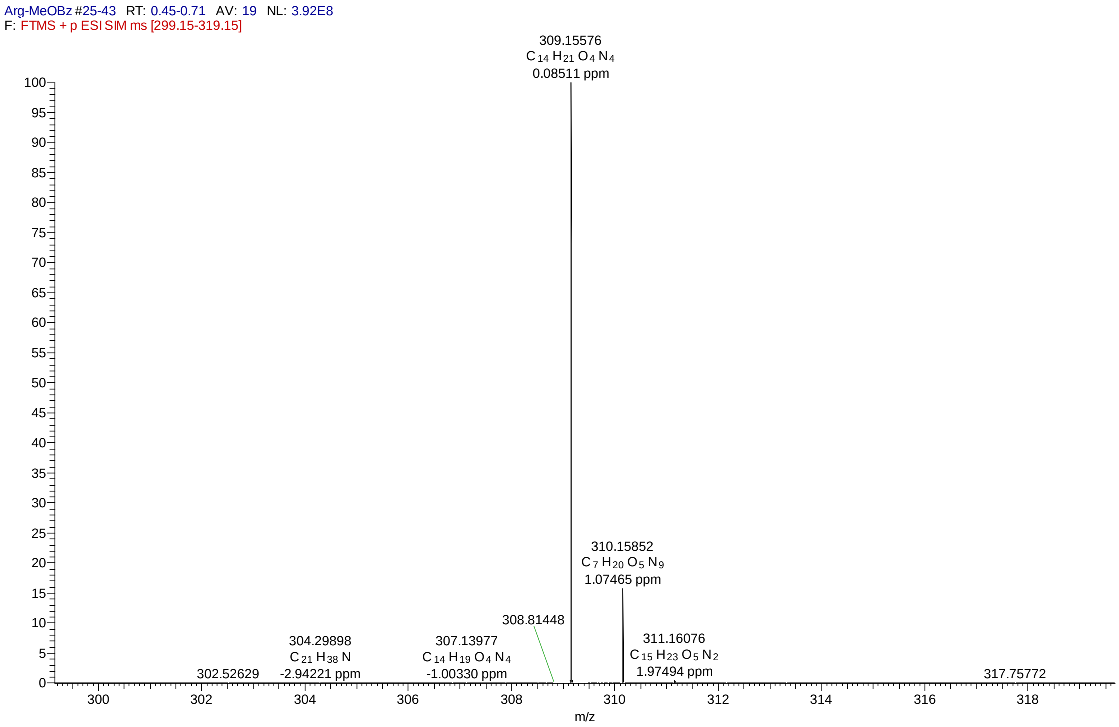

Compound 3c

^{1}H NMR (CD_3OD, 400 MHz) spectrum of compound **3c**

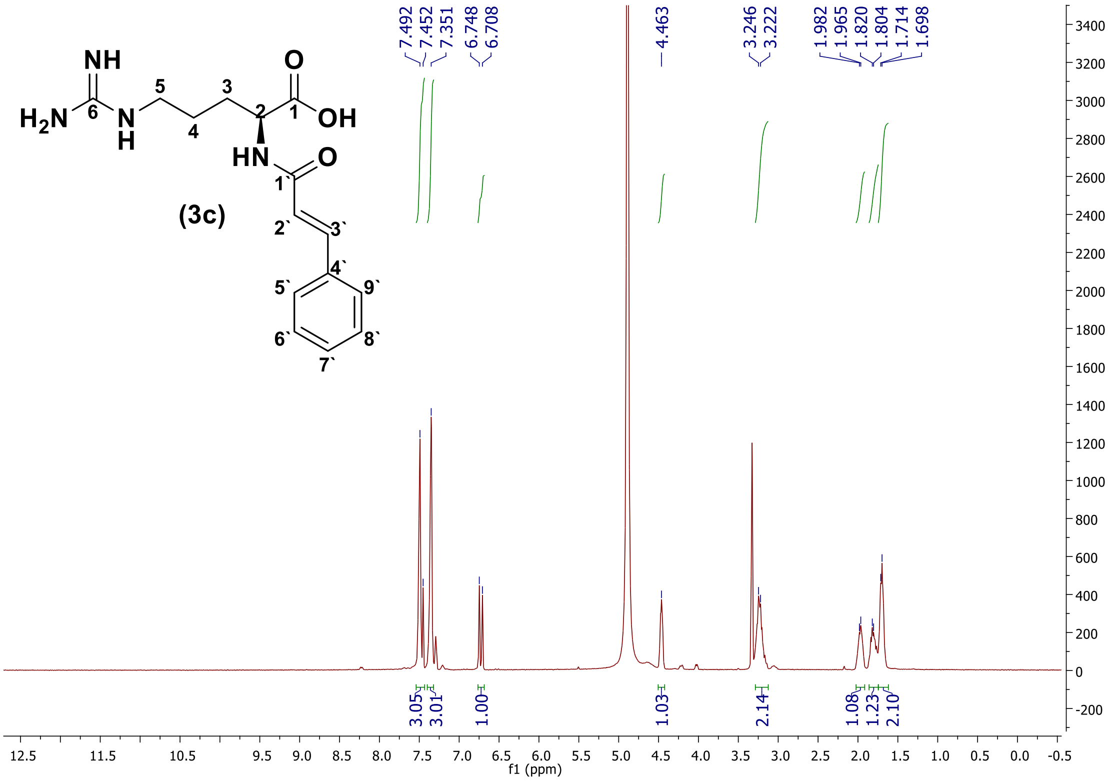

DEPT 135 NMR spectrum of compound **3c**

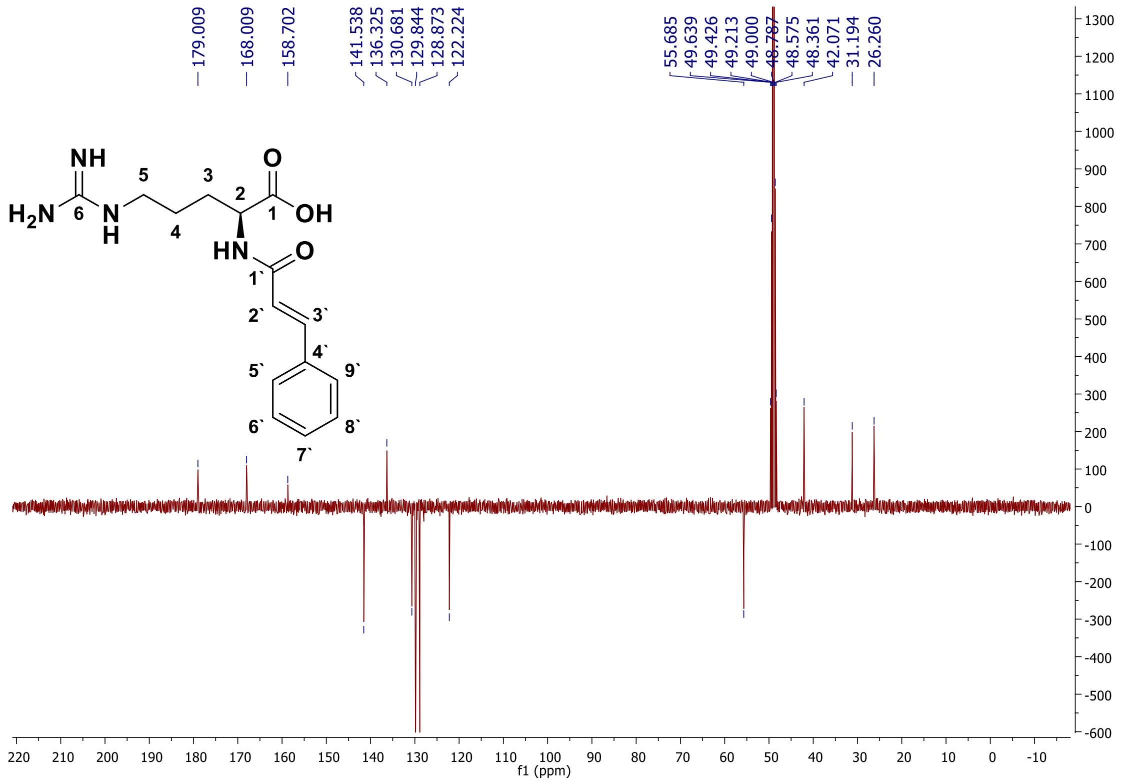

Compound 3d

^{1}H NMR (CD_3OD, 400 MHz) spectrum of compound **3d**

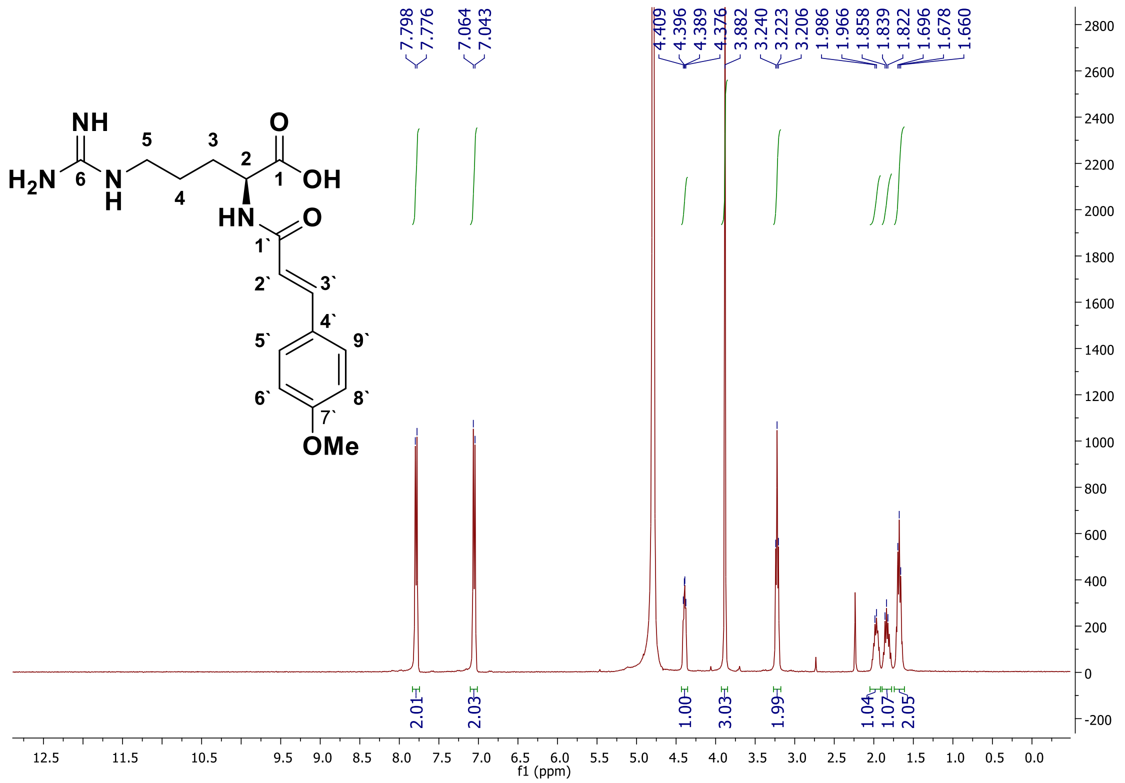

DEPT 135 NMR spectrum of compound **3d**

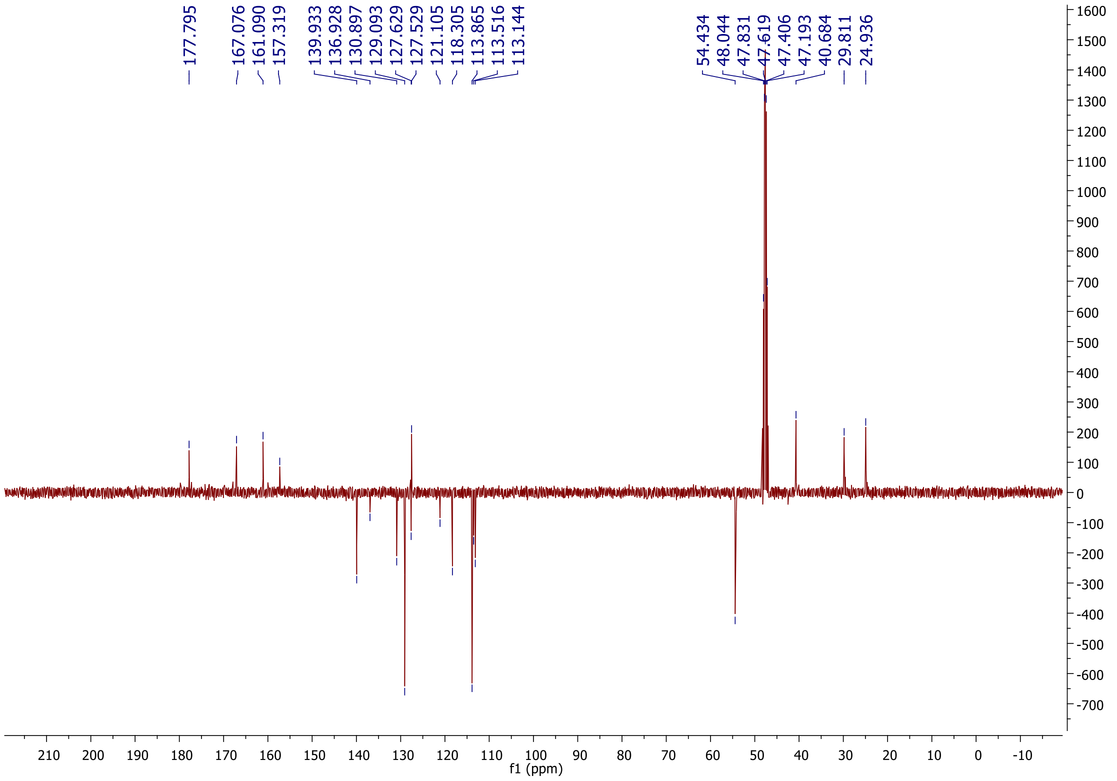

ESI-HRMS(+) of compound **3d**

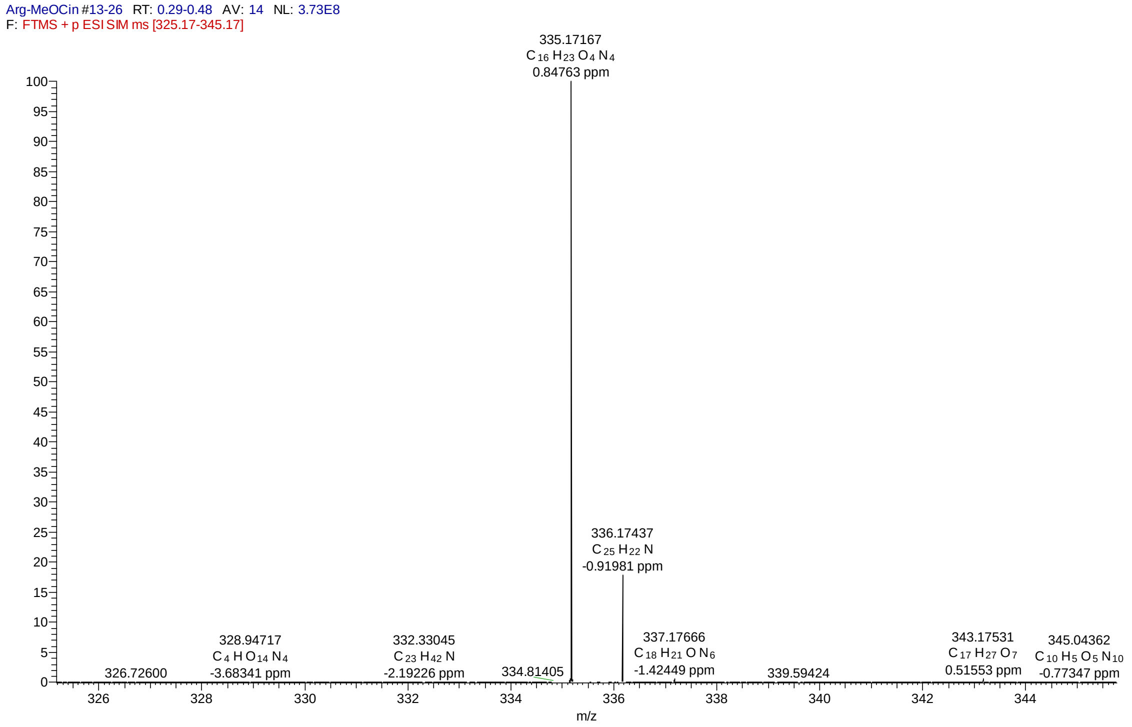

Compound 3e

^{1}H NMR (D_2O, 400 MHz) spectrum of compound **3e**

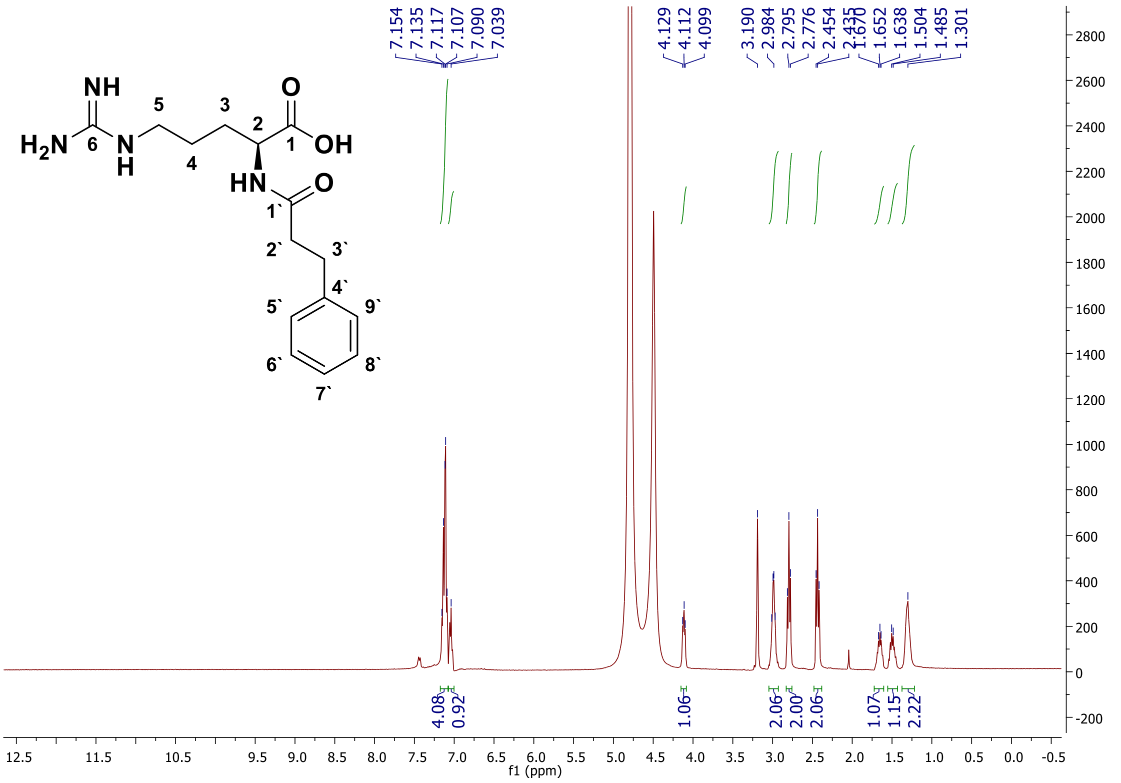

DEPT 135 NMR spectrum of compound **3e**

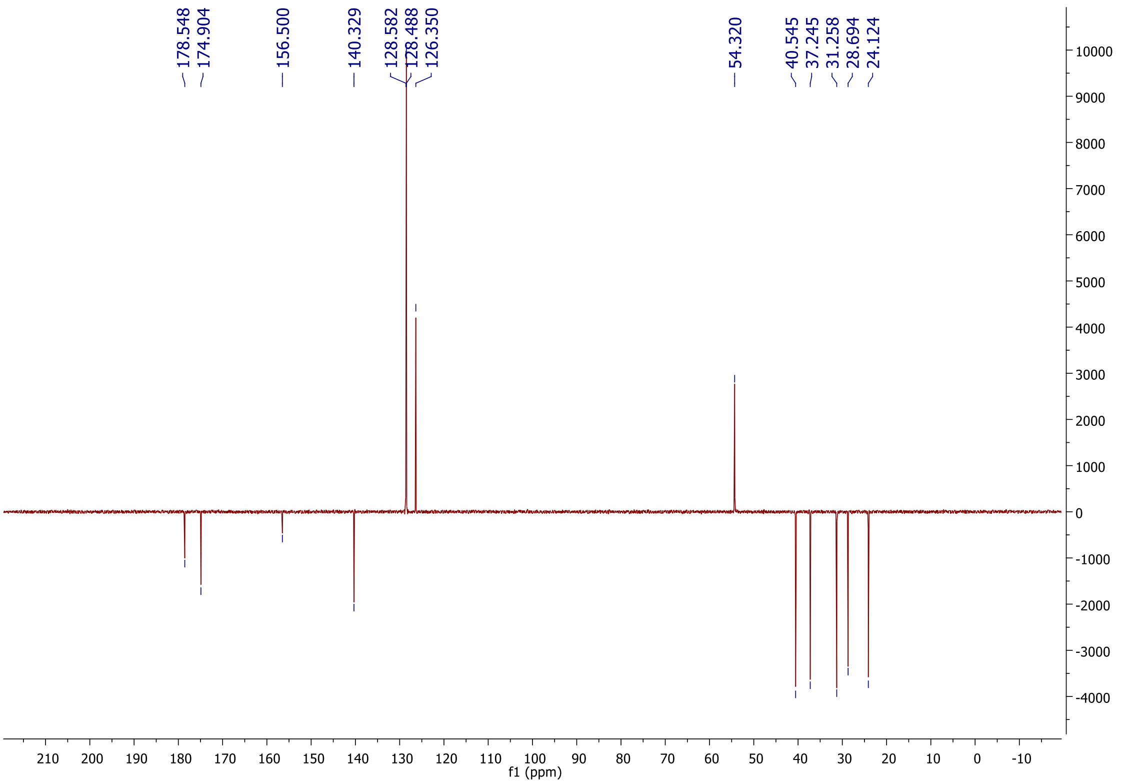

ESI-HRMS(+) of compound **3e**

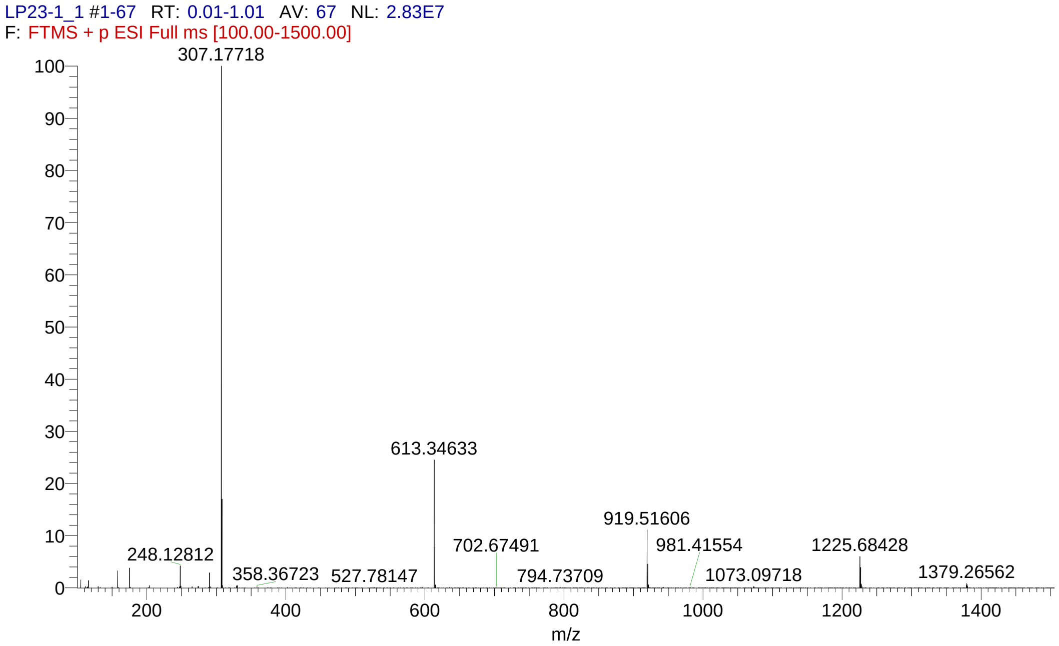

Compound 3f

^{1}H NMR (D_2O, 400 MHz) spectrum of compound **3f**

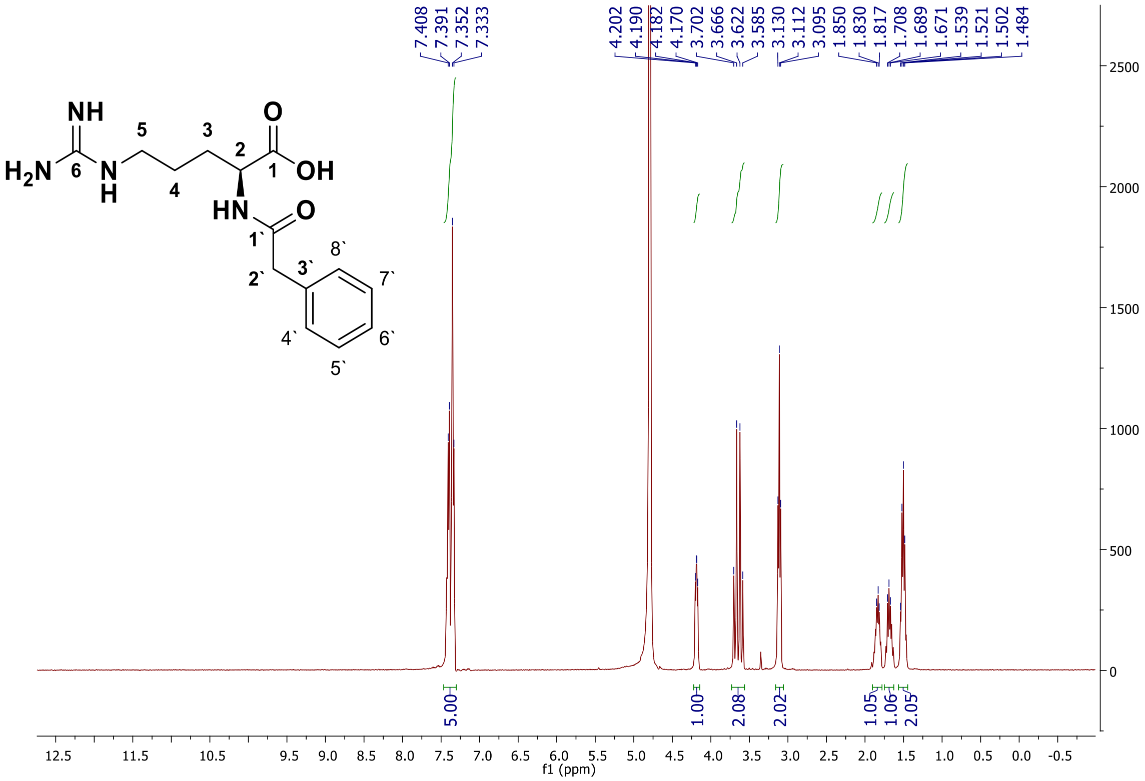

DEPT 135 NMR spectra of compound **3f**

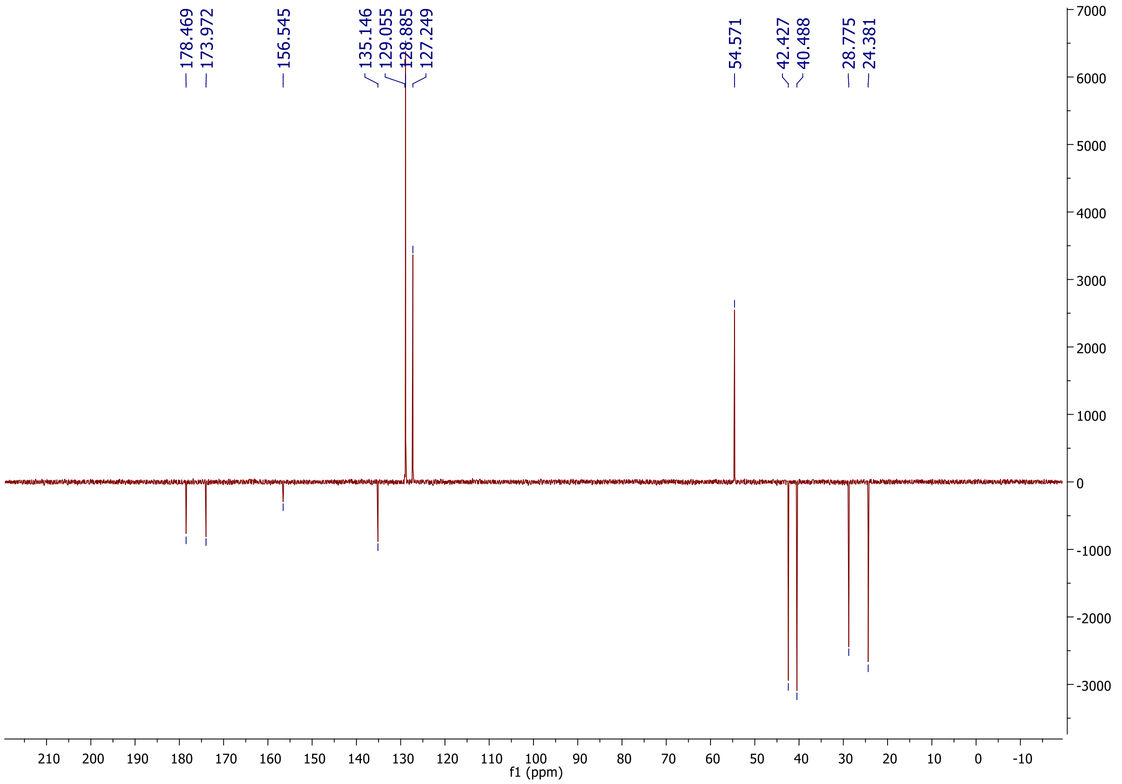

ESI-HRMS(+) of compound **3f**

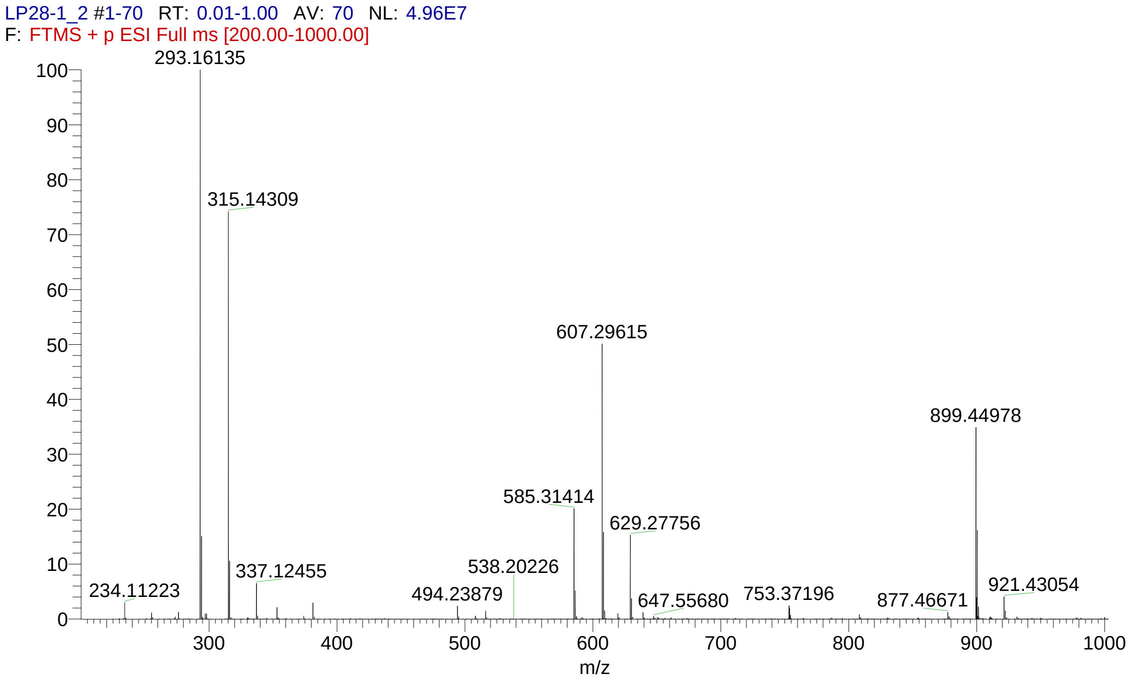

Compound 3g

^{1}H NMR (CD_3OD, 400 MHz) spectrum of compound **3g**

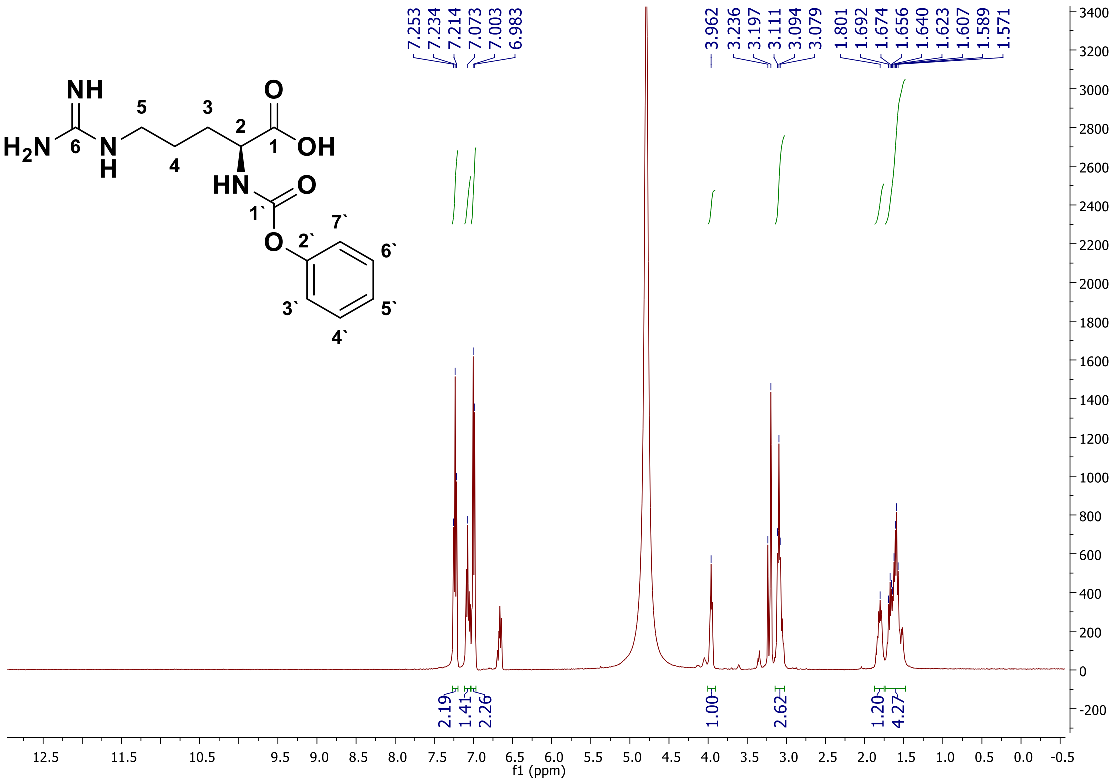

ESI-HRMS(+) of compound **3g**

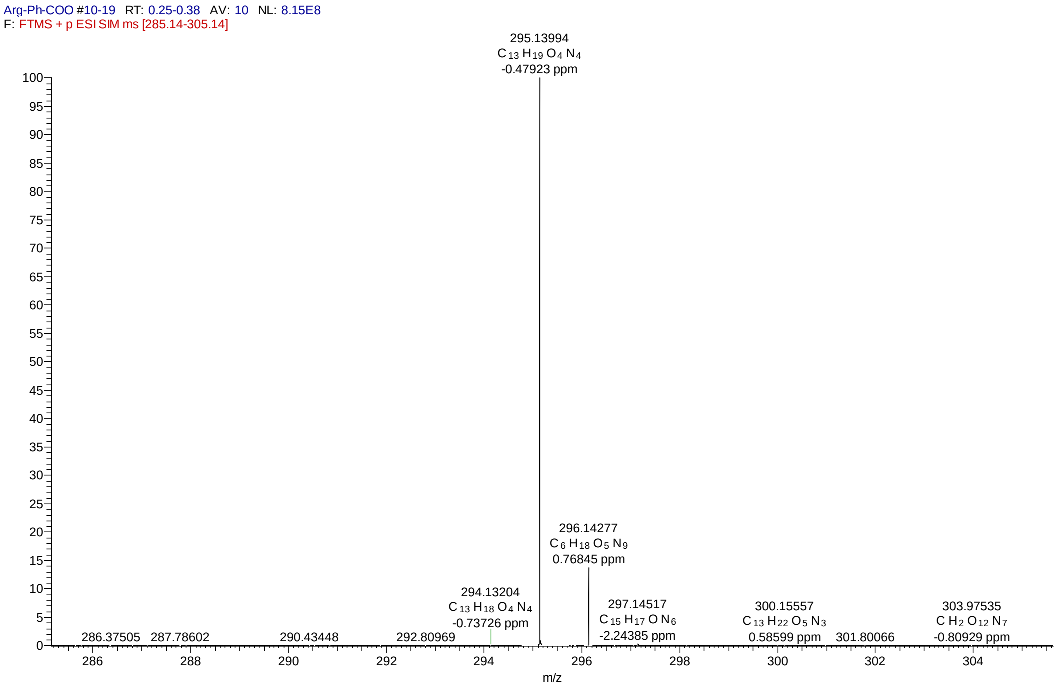

Compound 3h

^1H NMR (D_2O, 400 MHz) spectrum of compound **3h**

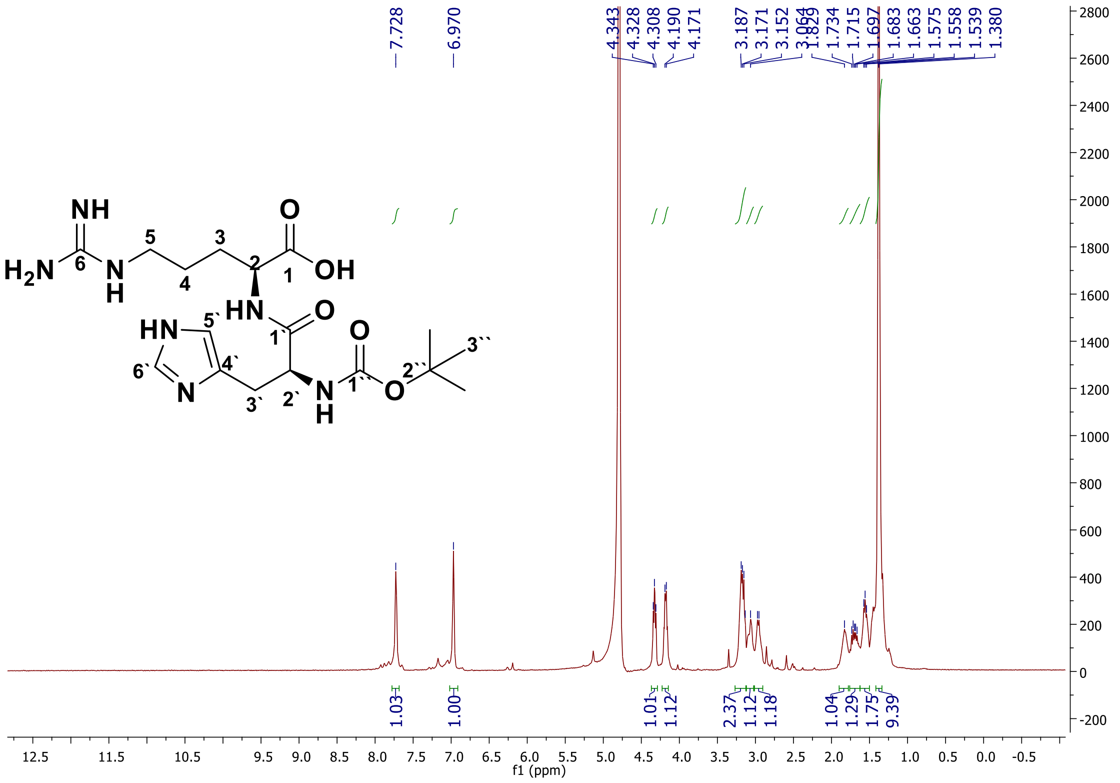

1.2. ^1H and ^{13}C NMR spectra of carbamimidoyl-L-proline (2)

^1H NMR (D_2O, 400 MHz) spectrum of compound **2**

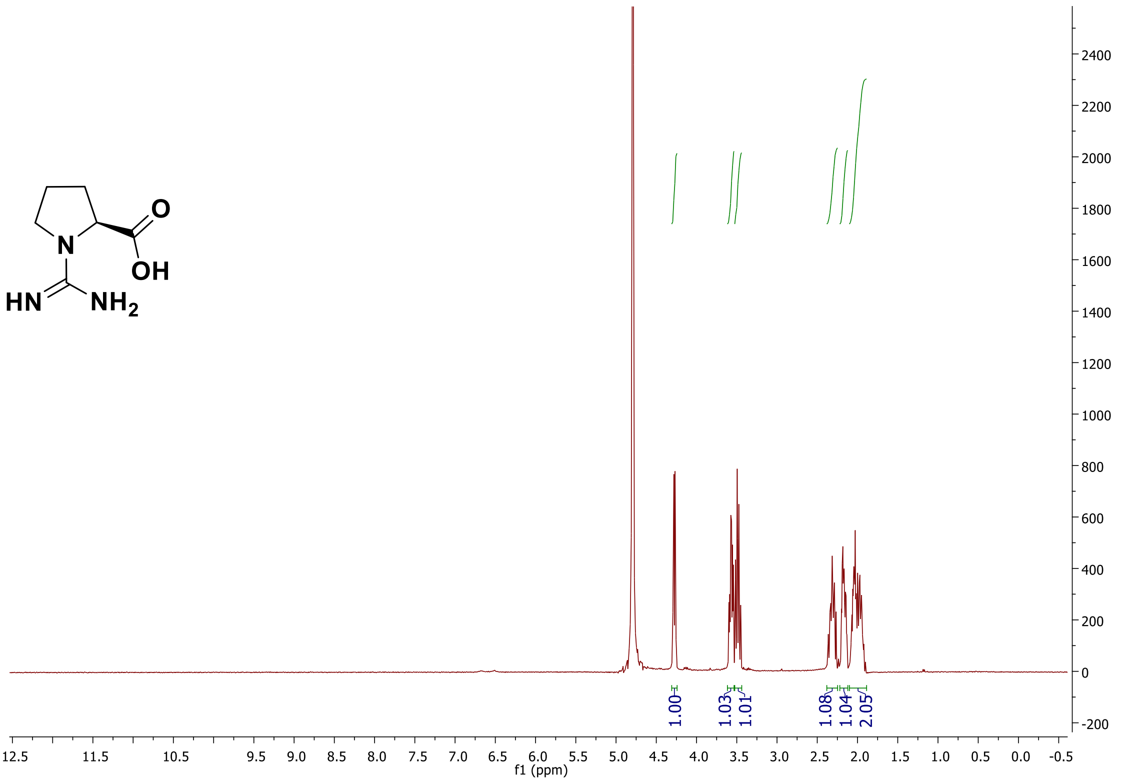

^{13}C NMR (D_2O, 101 MHz) spectrum of compound **2**

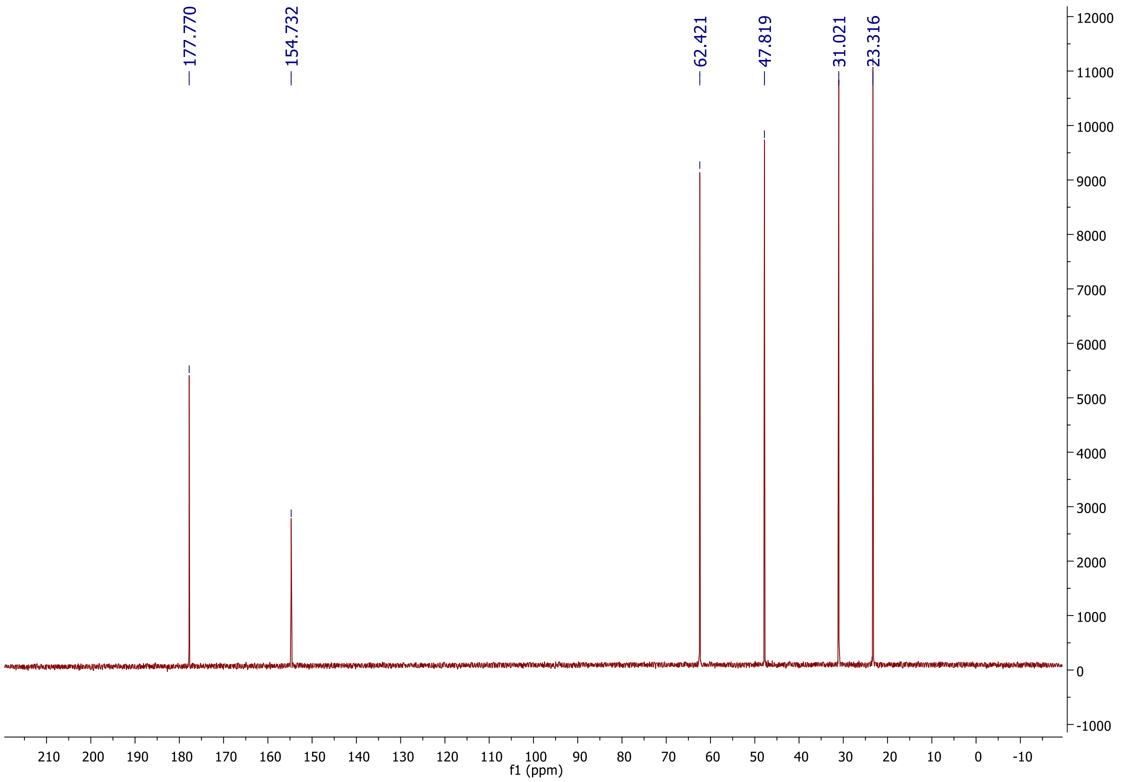

1.3. ^{1}H, ^{13}C NMR and Mass spectra of compounds 4 and Cernumidine (1)

^{1}H NMR (CD_3OD, 400 MHz) spectrum of Cernumidine (**1**)

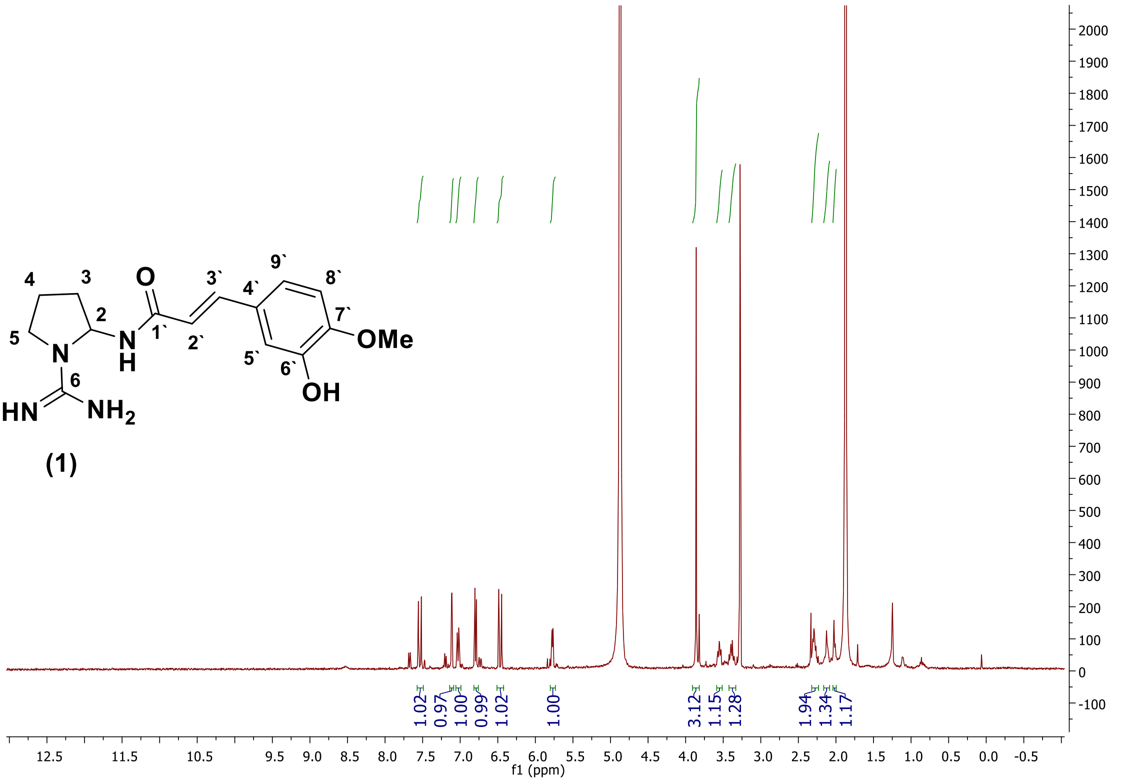

^{13}C NMR (CD_3OD, 400 MHz) spectrum of Cernumidine (**1**)

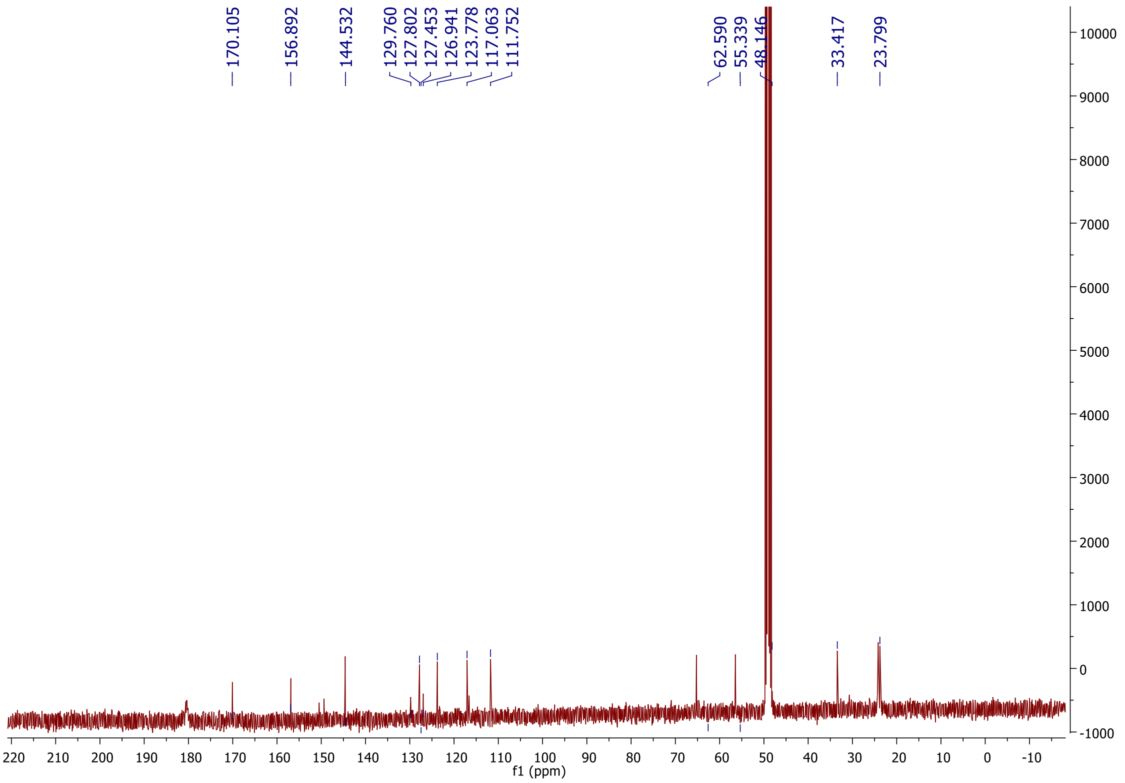

ESI-MS(+) Cernumidine (**1**)

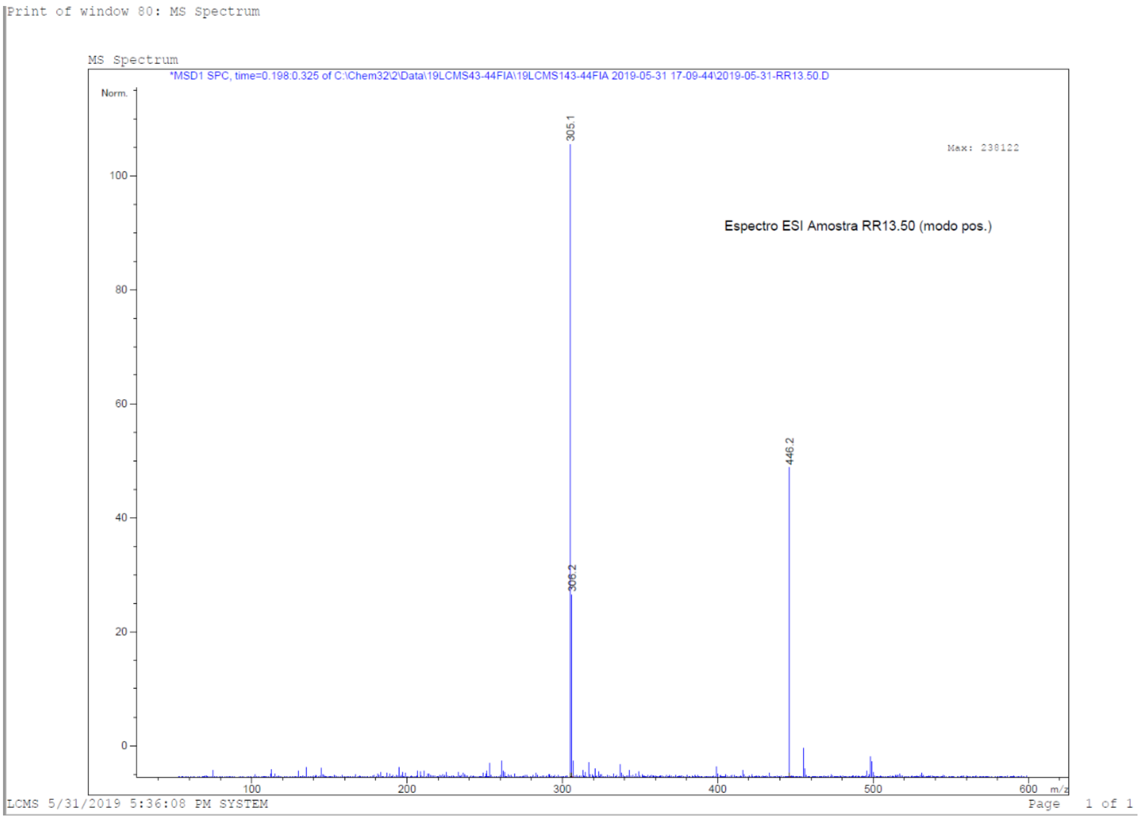

Compound 4a

^{1}H NMR (D_2O, 400 MHz) spectrum of compound **4a**

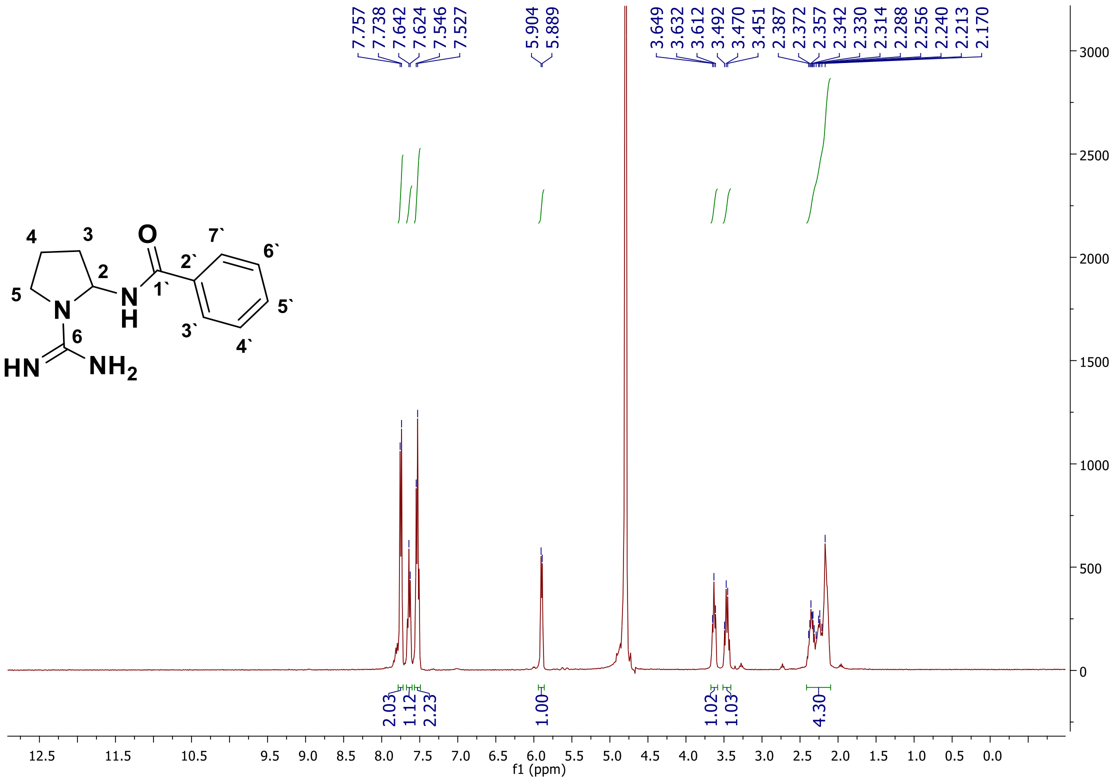

^{13}C NMR (D_2O, 101 MHz) spectrum of compound **4a**

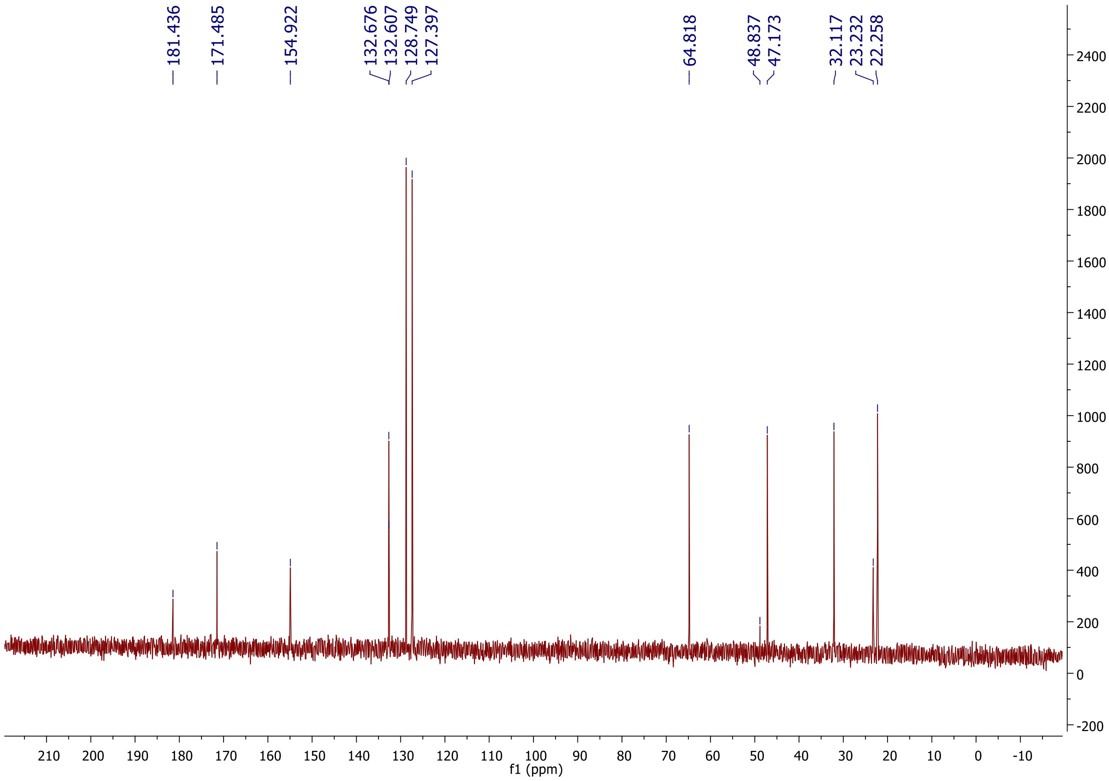

ESI-HRMS(+) of compound **4a**

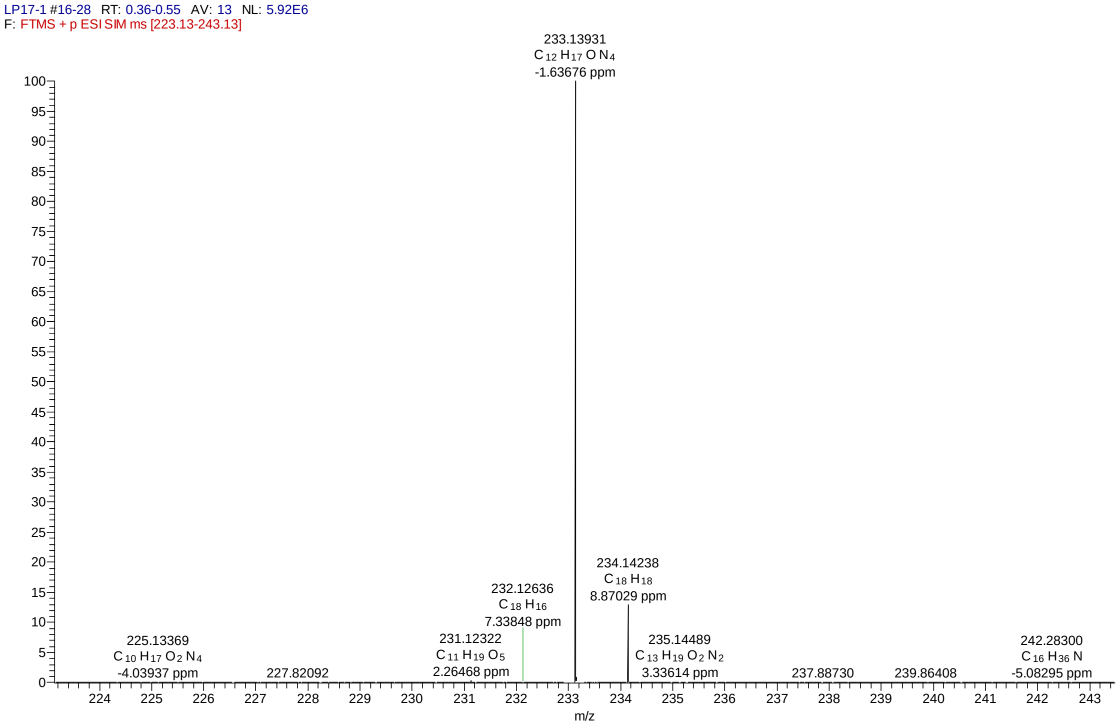

Compound 4b

^{1}H NMR (D_2O, 400 MHz) spectrum of compound **4b**

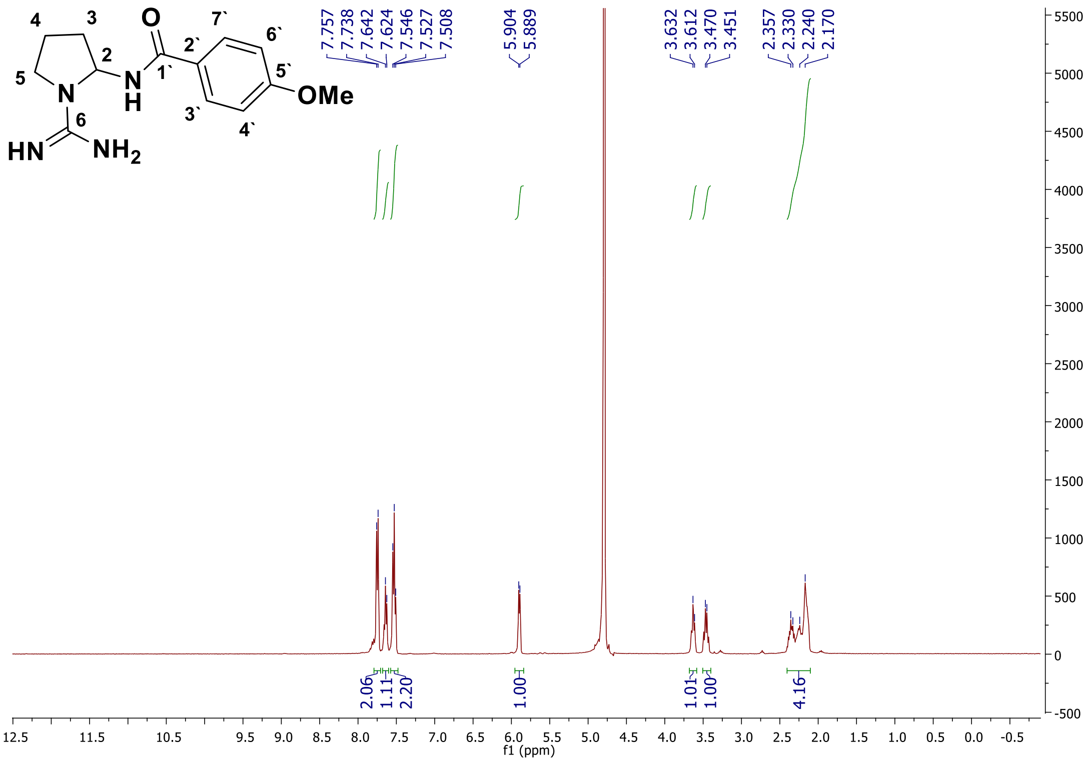

^{13}C NMR (D_2O, 101 MHz) spectrum of compound **4b**

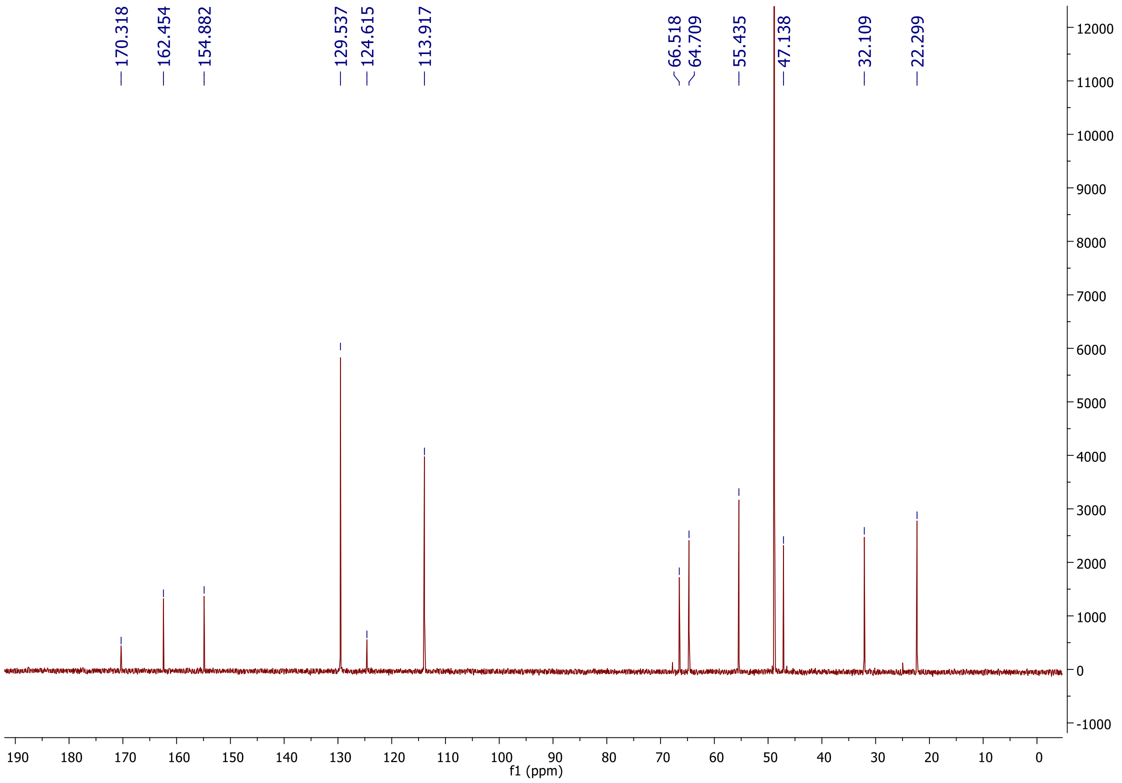

ESI-HRMS(+) of compound **4b**

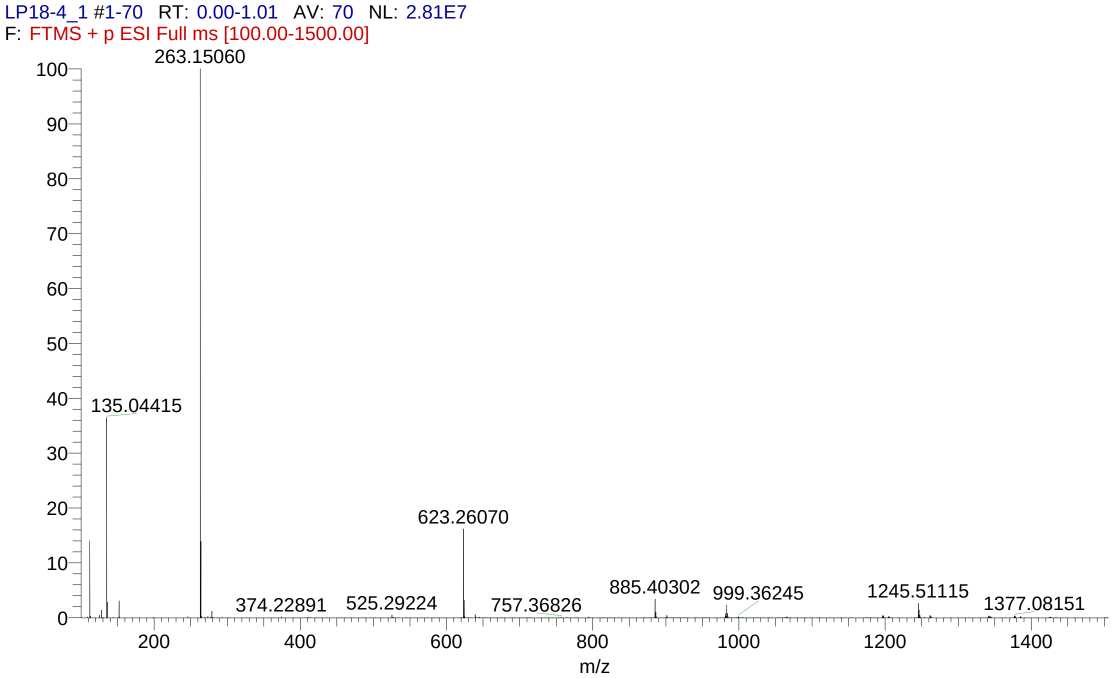

Compound 4c

^{1}H NMR (CD_3OD, 400 MHz) spectrum of compound **4c**

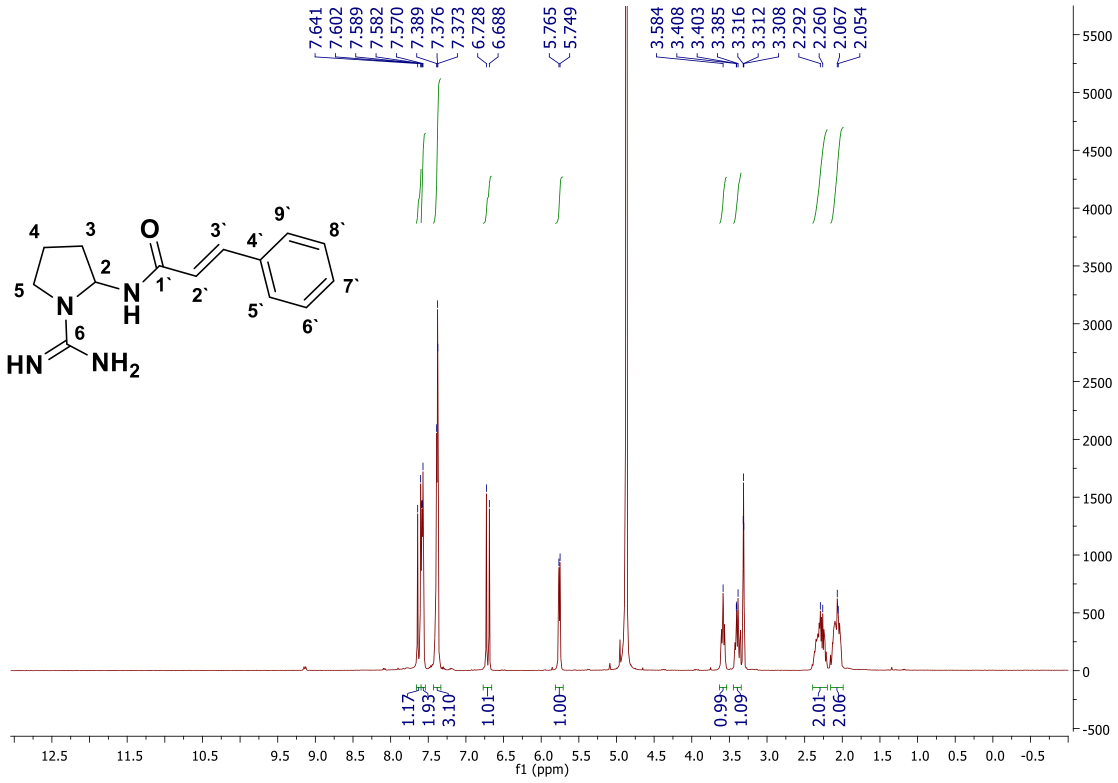

^{13}C NMR (CD_3OD, 101 MHz) spectrum of compound **4c**

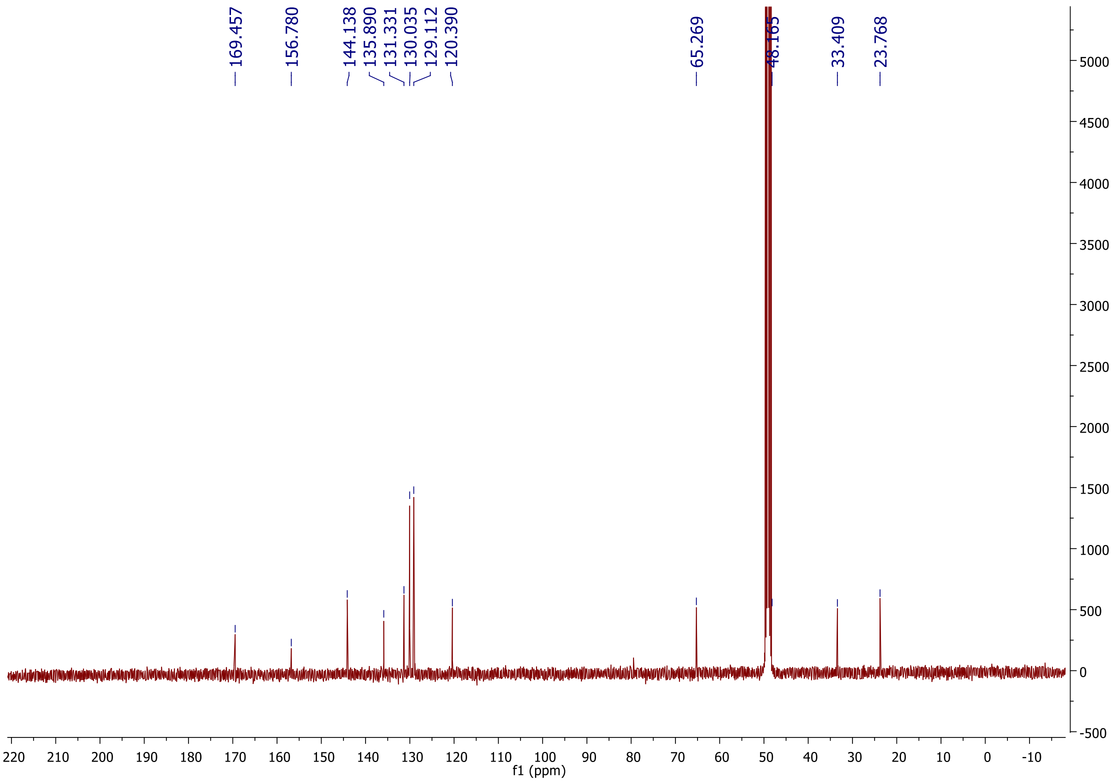

ESI-MS(+) compound **4c**

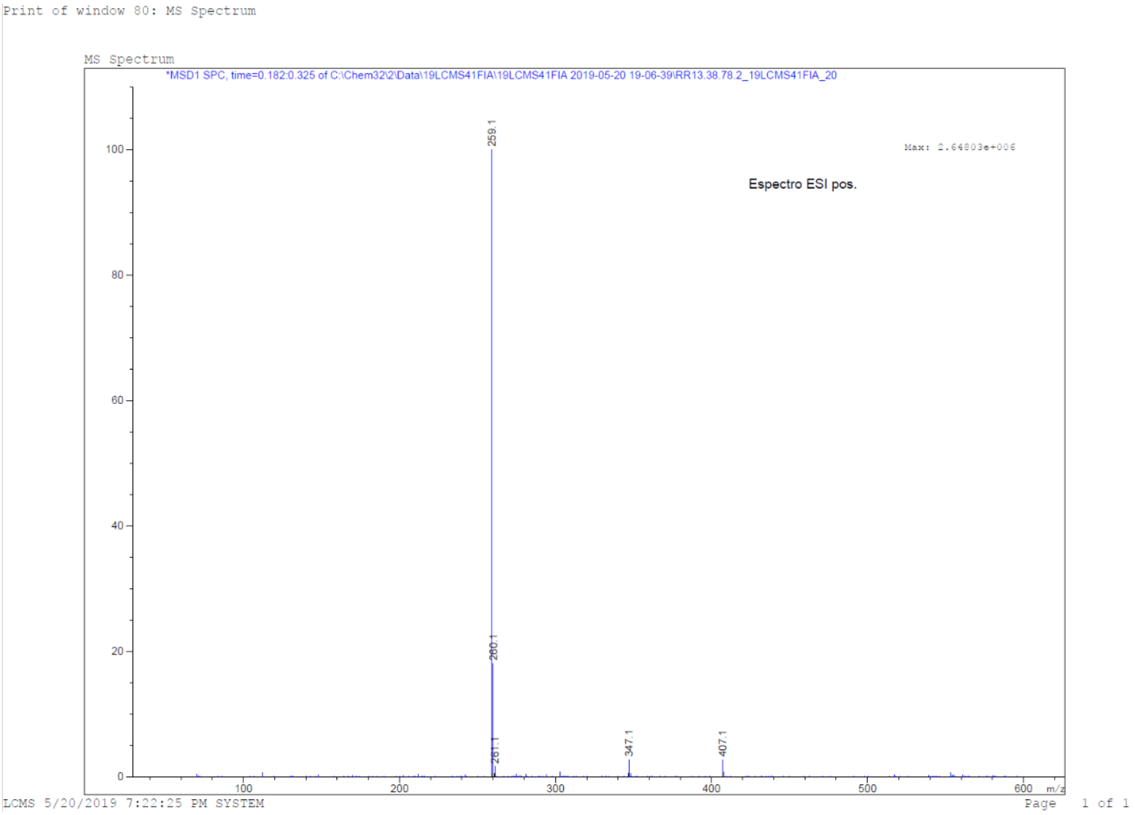

ESI-HRMS(+) of compound **4c**

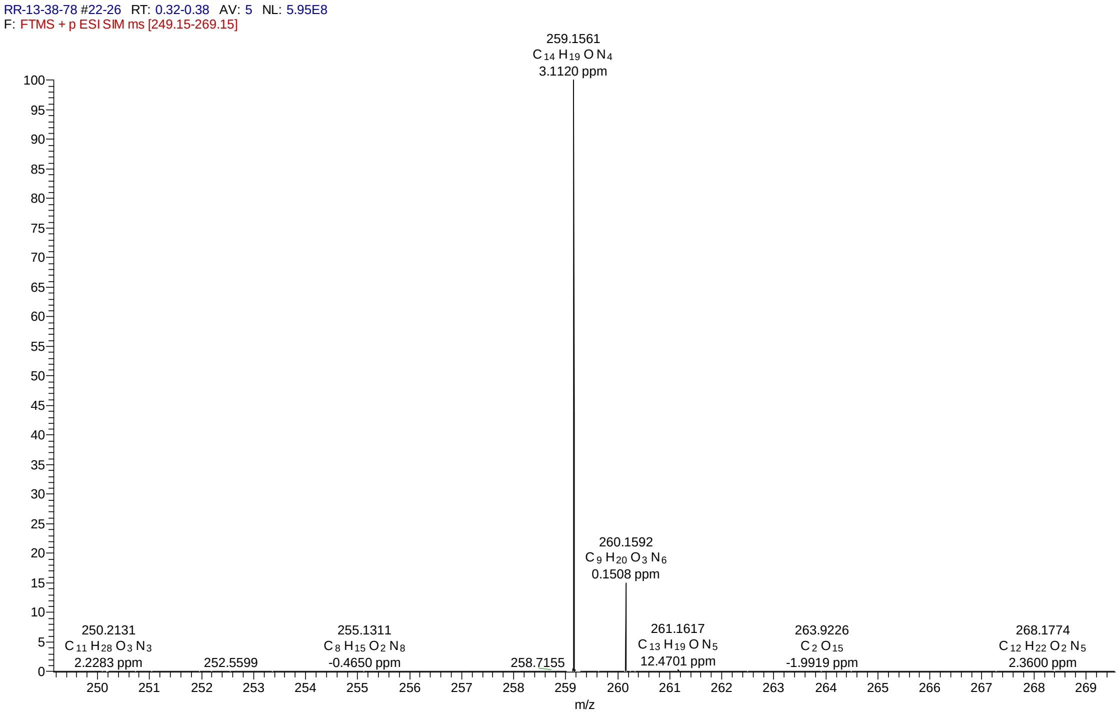

Compound 4d

^{1}H NMR (CD_3OD, 400 MHz) spectrum of compound **4d**

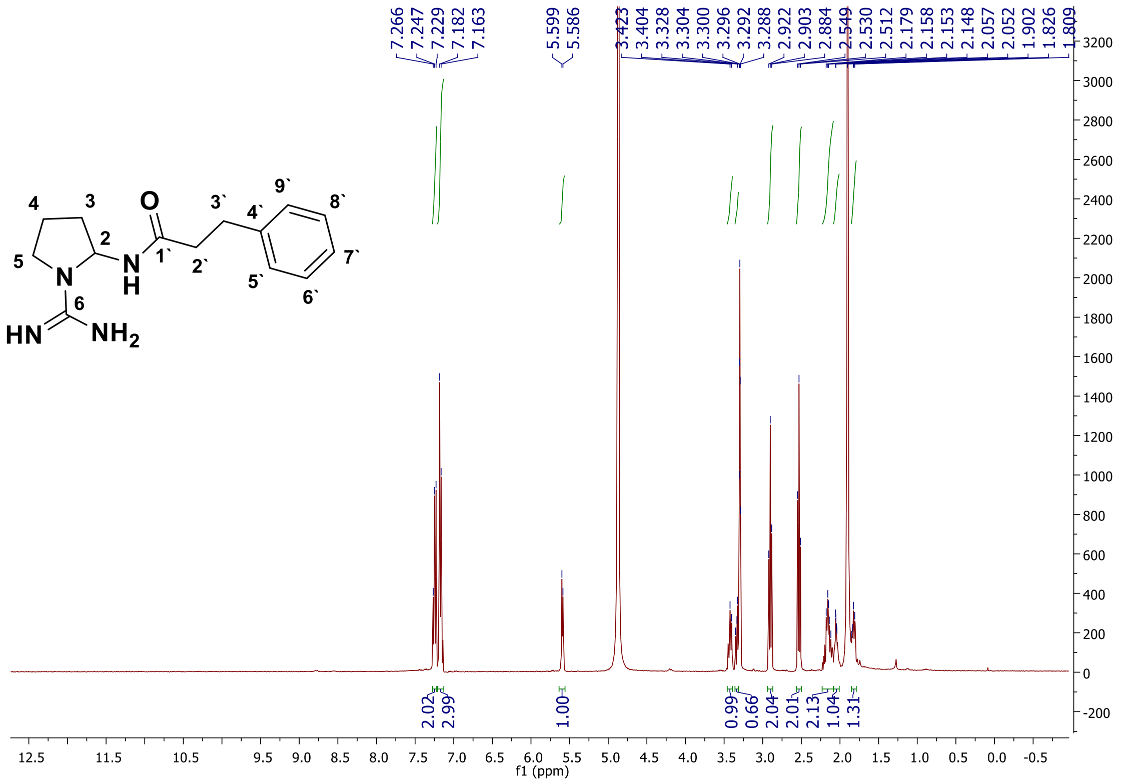

^{13}C NMR (CD_3OD, 101 MHz) spectrum of compound **4d**

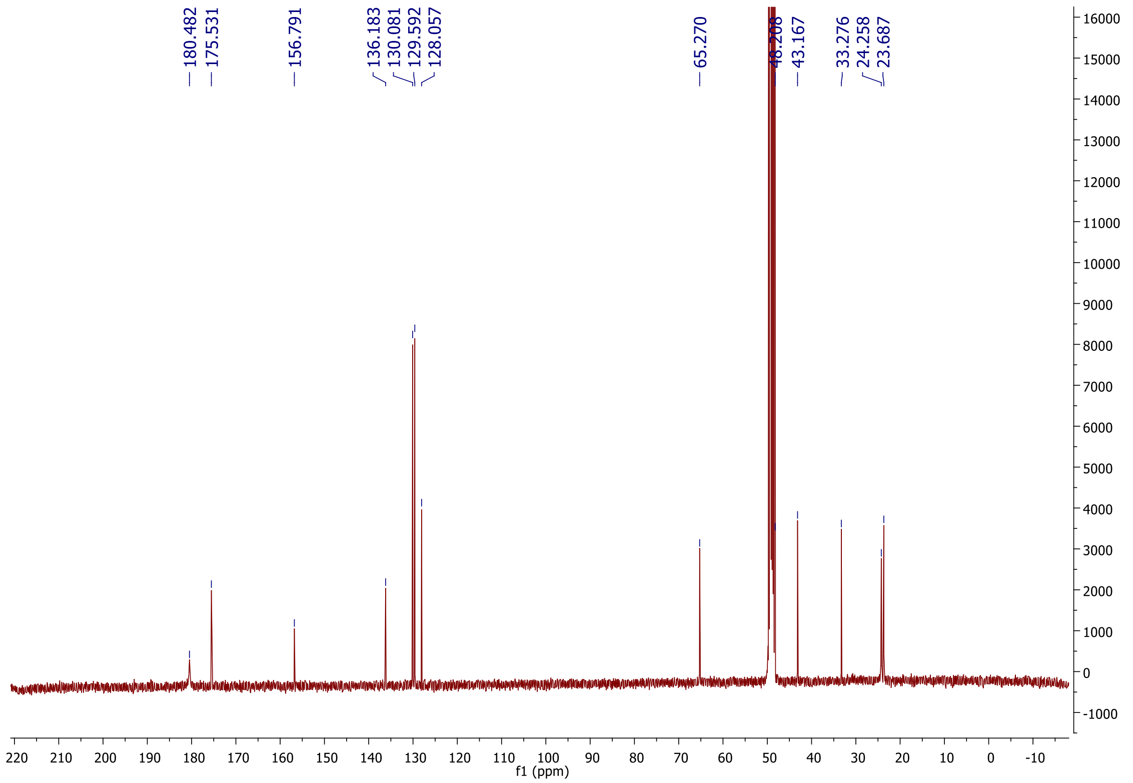

ESI-MS(+) compound **4d**

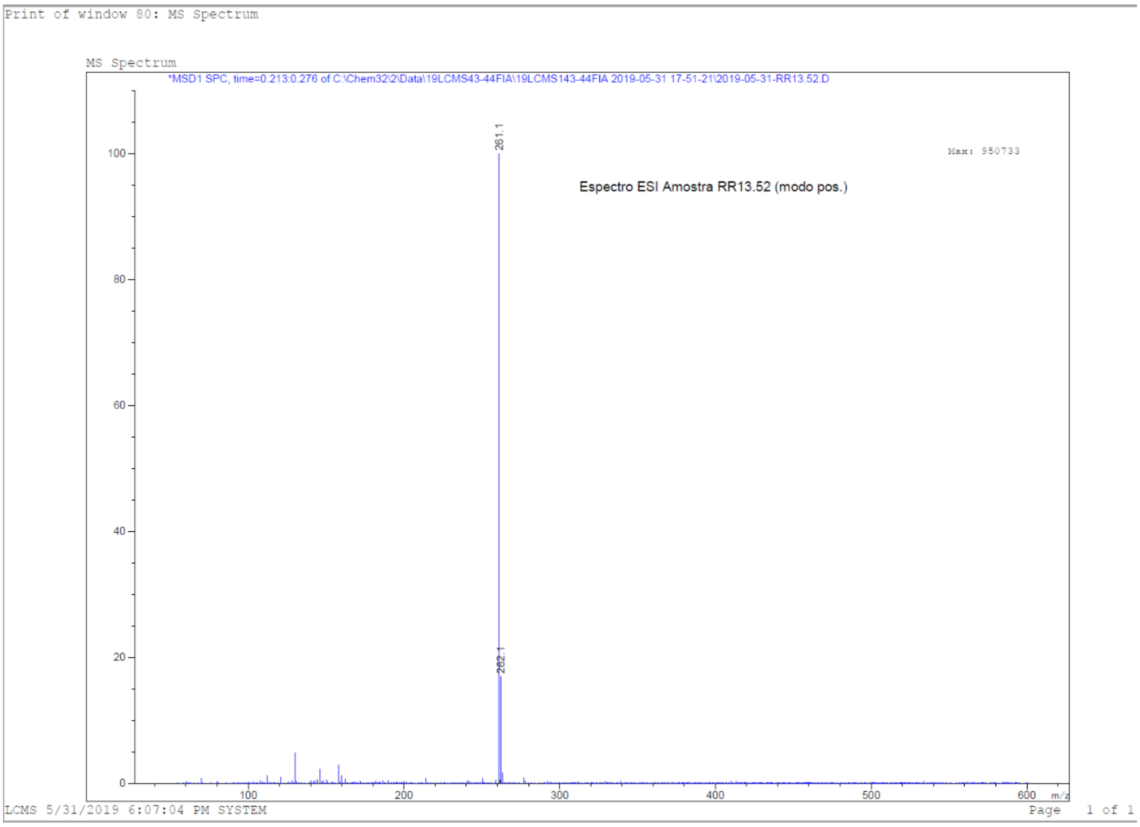

ESI-HRMS(+) of compound **4d**

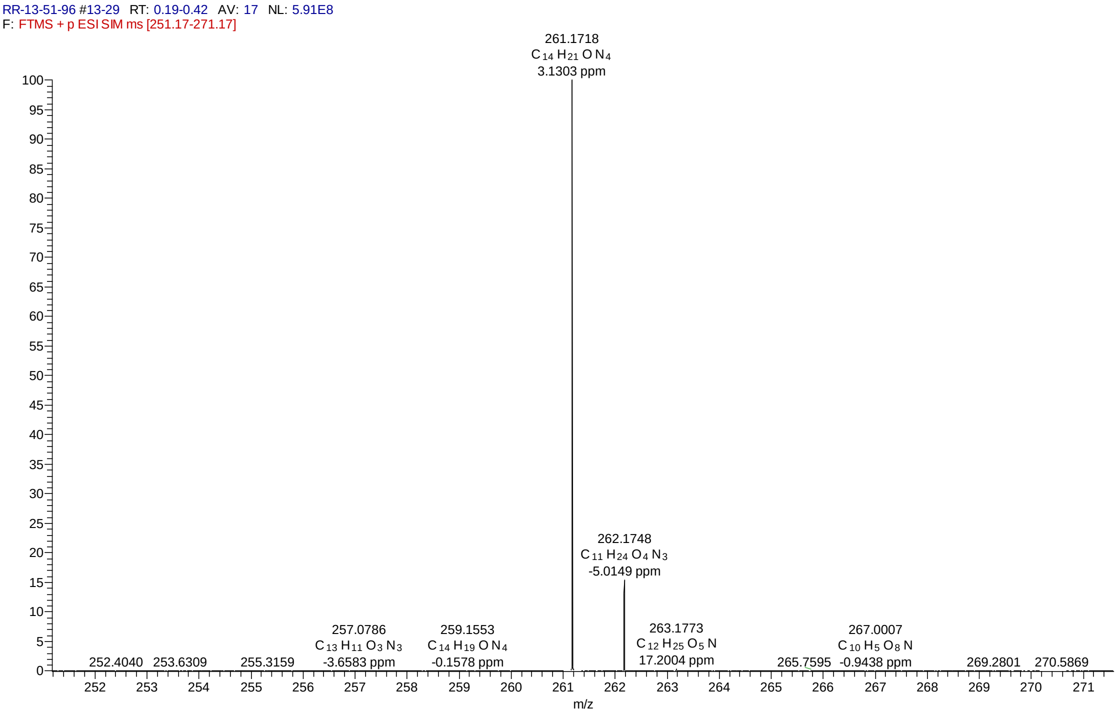

Compound 4e

^{1}H NMR (CD_3OD, 400 MHz) spectrum of compound **4e**

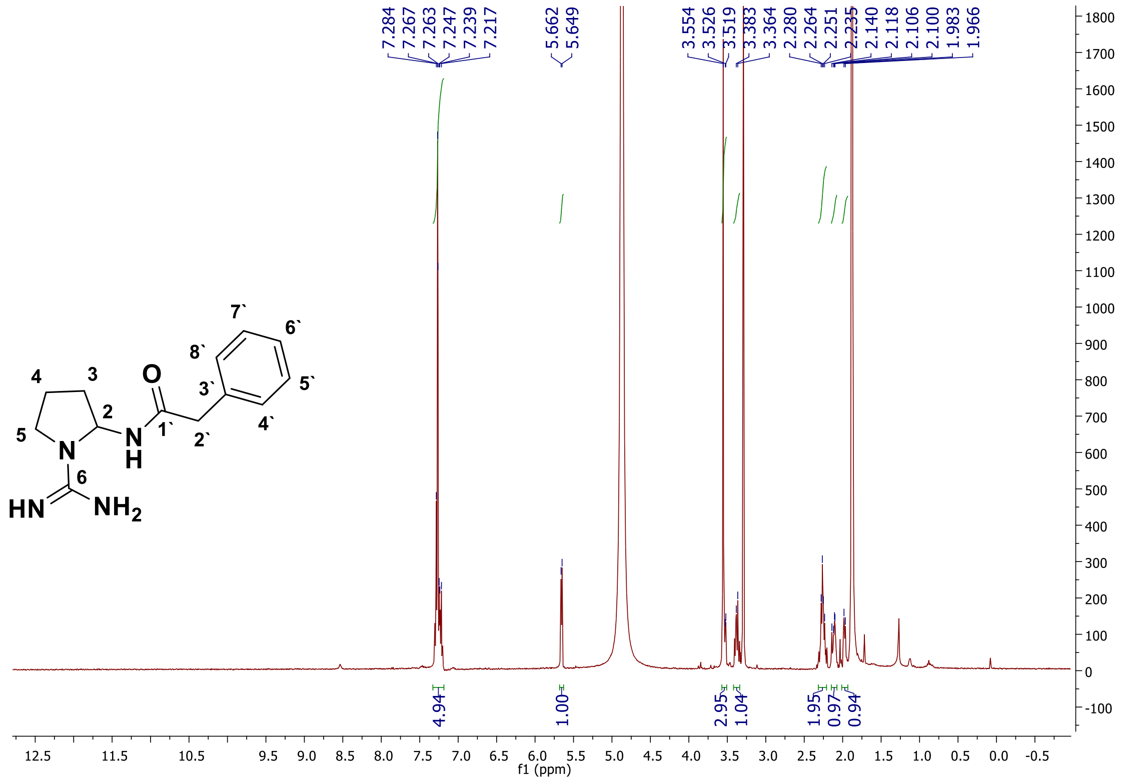

^{13}C NMR (CD_3OD, 400 MHz) spectrum of compound **4e**

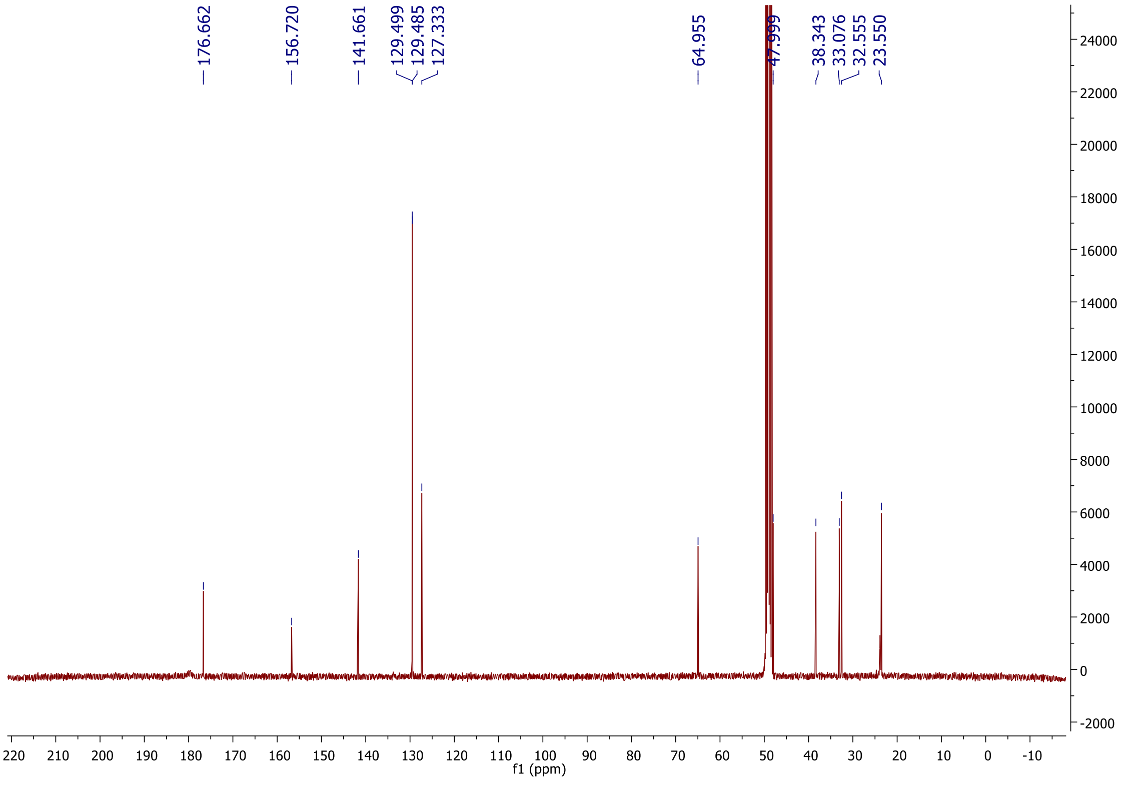

ESI-HRMS(+) of compound **4e**

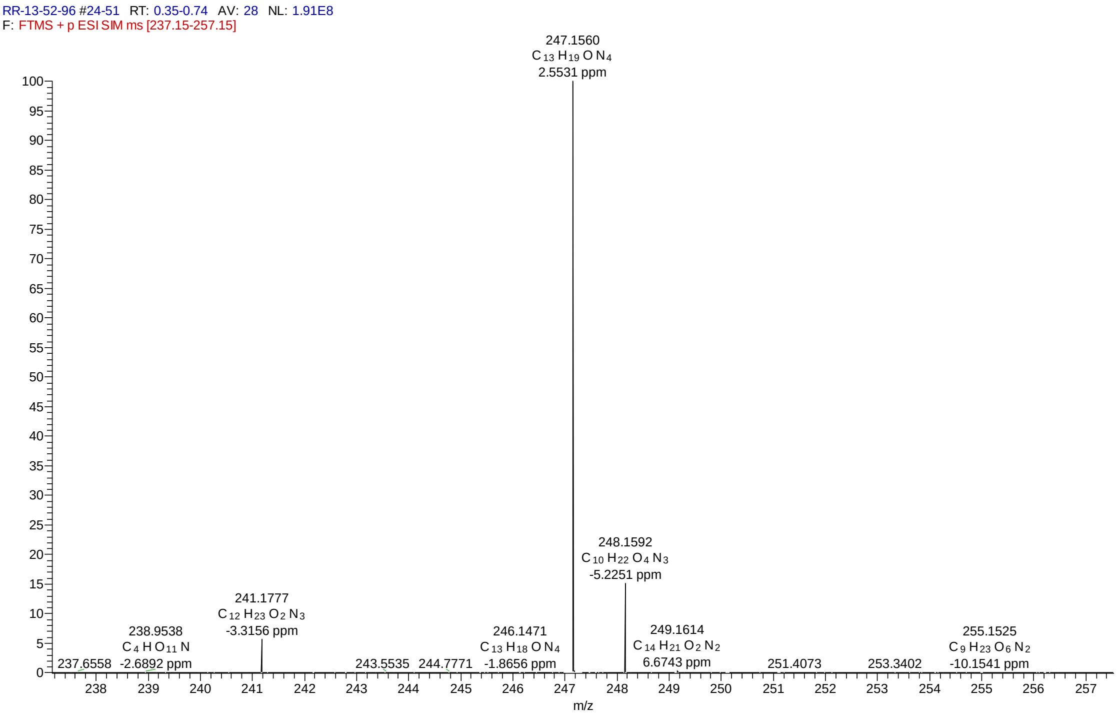

Compound 4f

^{1}H NMR (D_2O, 400 MHz) spectrum of compound **4f**

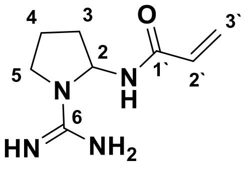

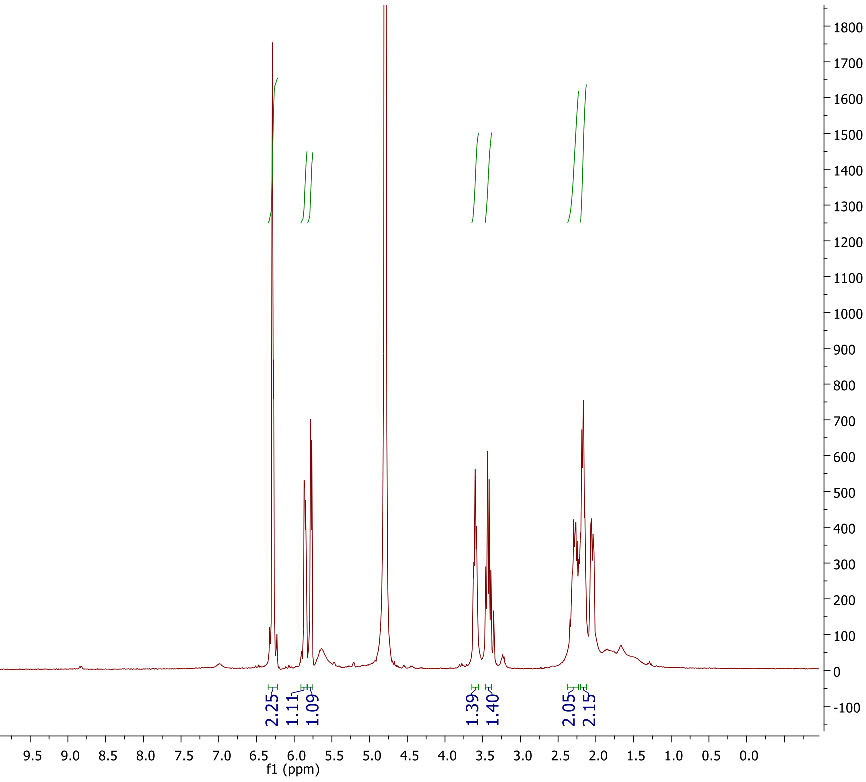

^{13}C NMR (D_2O, 101 MHz) spectrum of compound **4f**

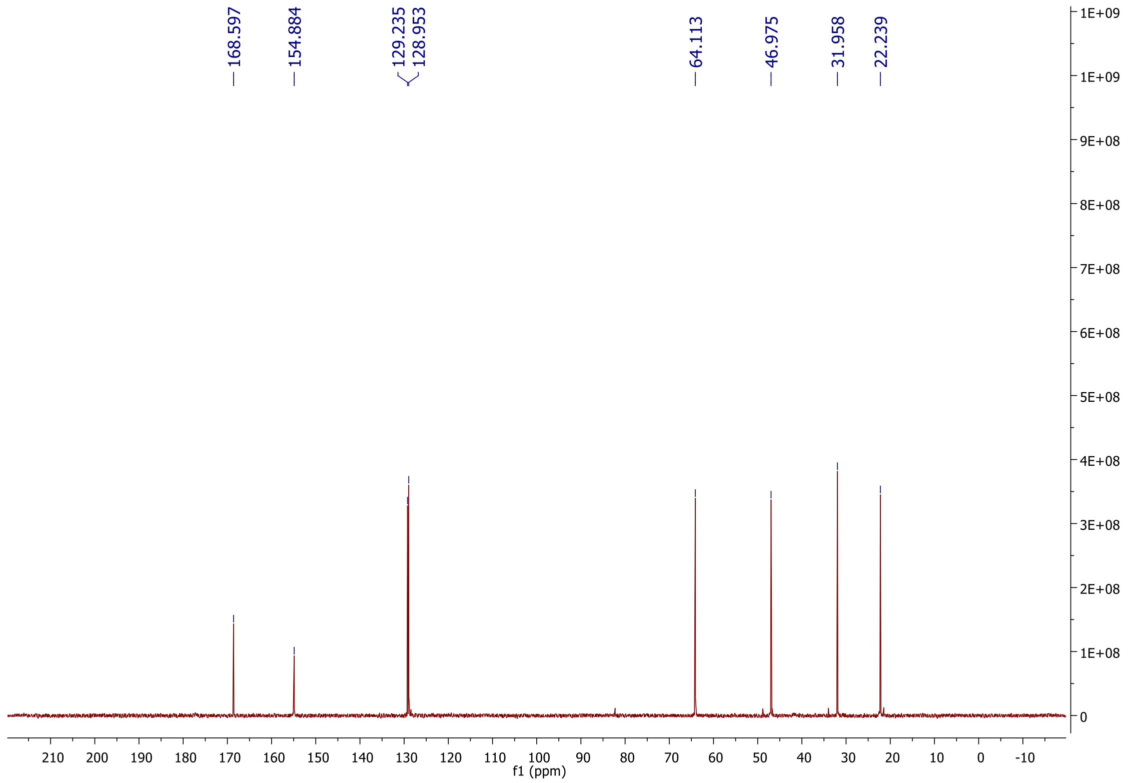

ESI-HRMS(+) of compound **4f**

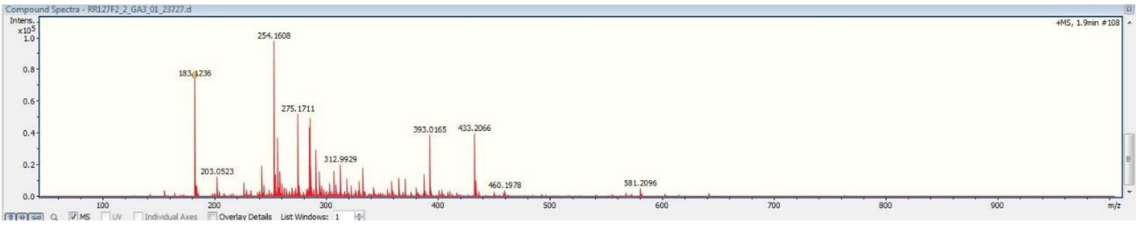

MF C8H14N4O

MW 182.1167

accurate mass	ion formula	exact mass	err (ppm)
183.1236	C8H15N4O	183.1240	2.2

Compound 4g

^{1}H NMR (D_2O, 400 MHz) spectrum of compound **4g**

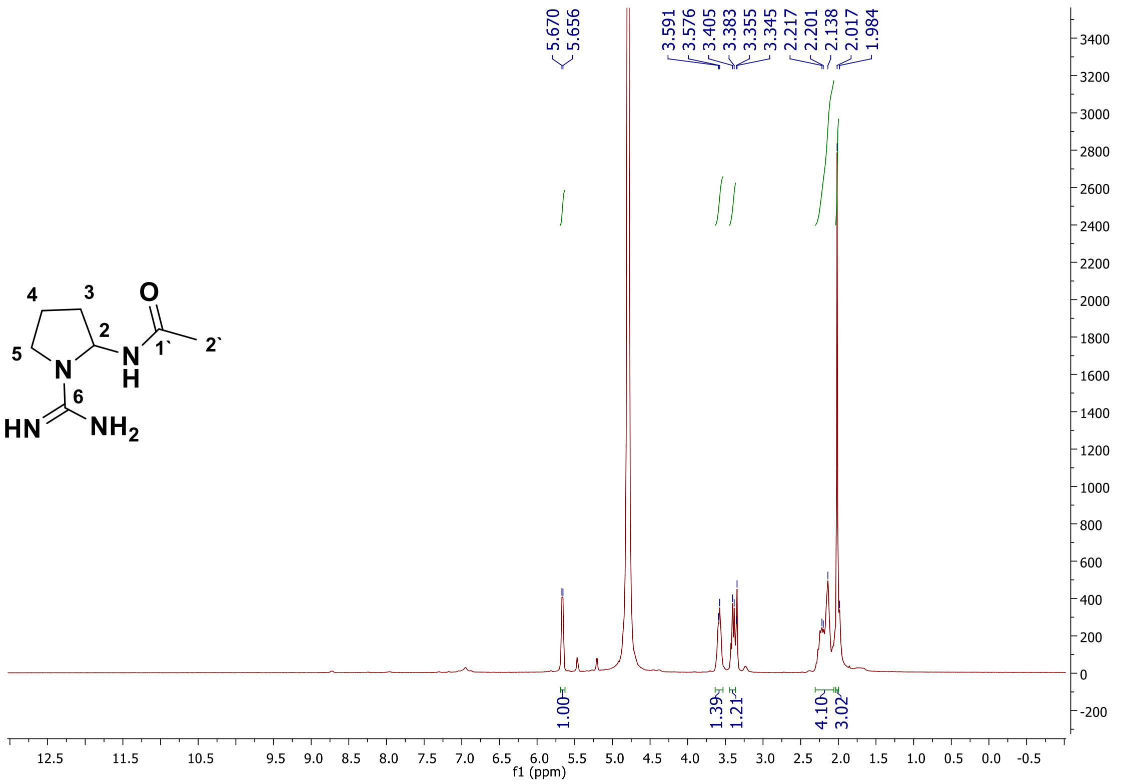

^{13}C NMR (D_2O, 101 MHz) spectrum of compound **4g**

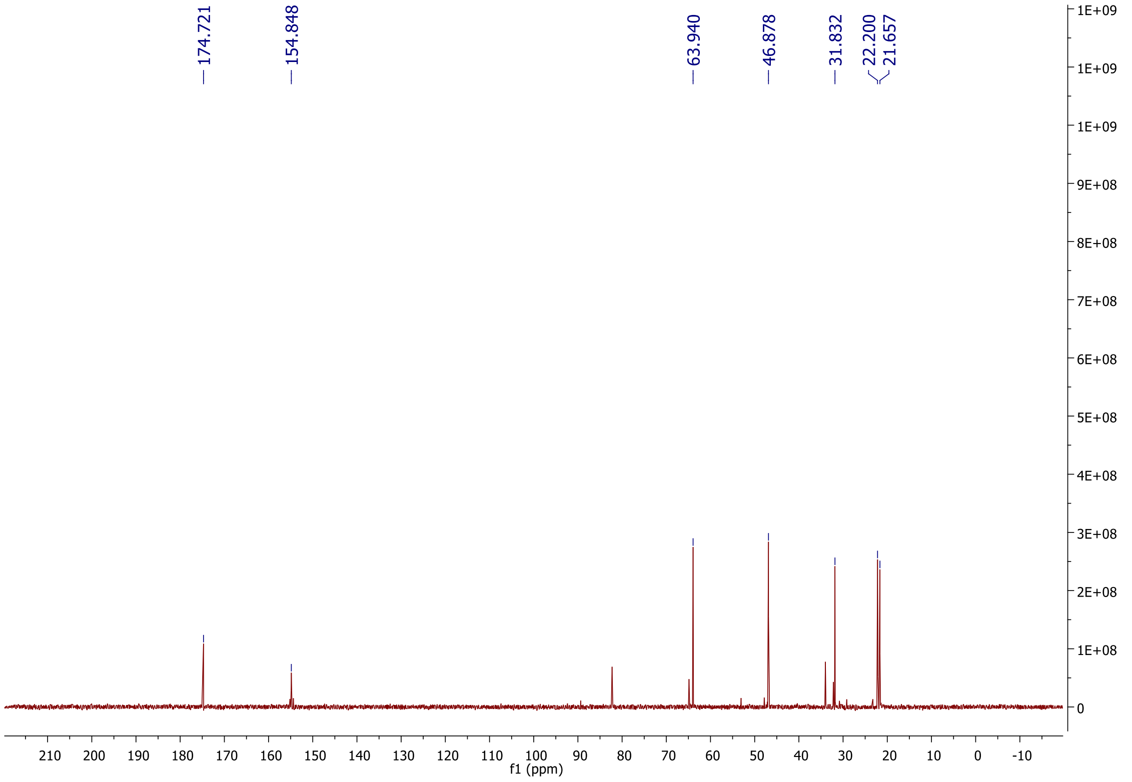

ESI-HRMS(+) of compound **4g**

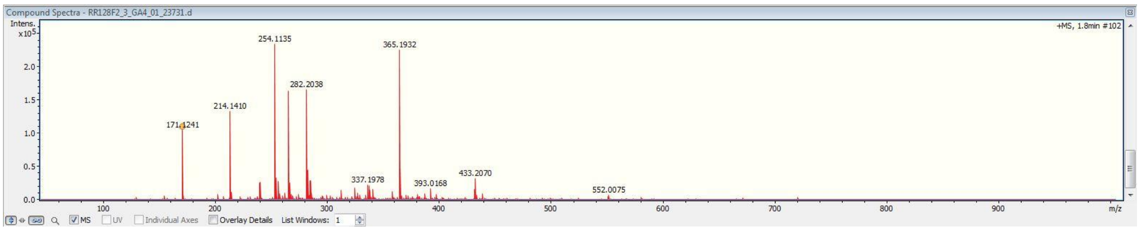

MF $C_7H_{14}N_4O$
MW 170.1167

accurate mass	ion formula	exact mass	err (ppm)
171.1241	$C_7H_{15}N_4O$	171.1240	-0.4

Compound 4h

^{1}H NMR (D_2O, 400 MHz) spectrum of compound **4h**

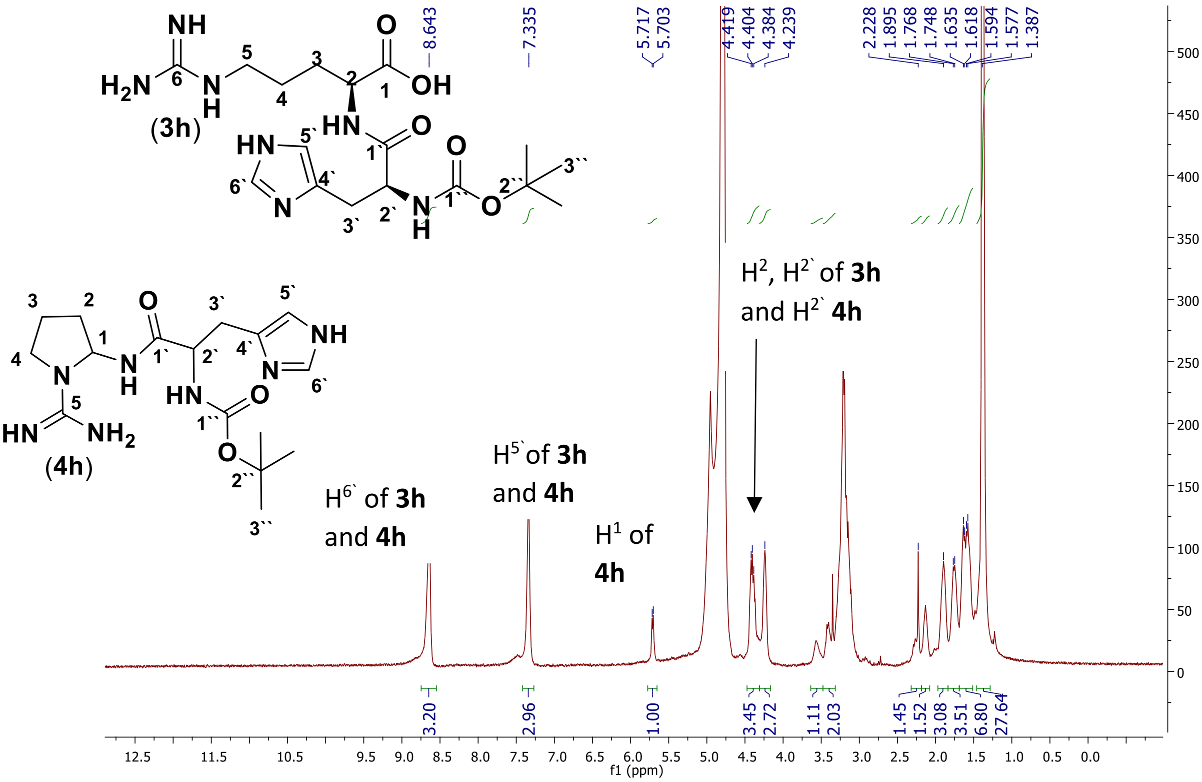

ESI-HRMS(+) of compound **4h**

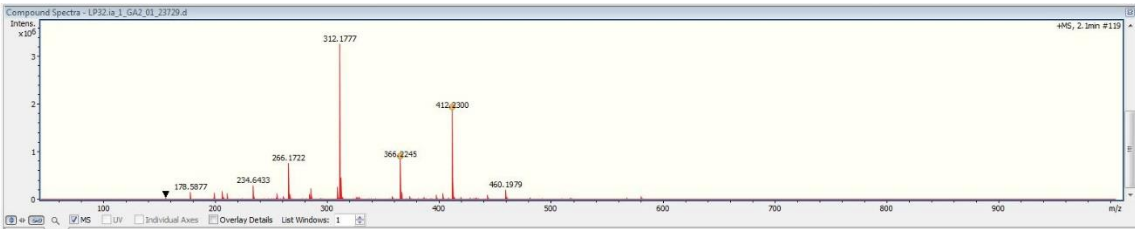

MF $C_{16}H_{27}N_7O_3$ and $C_{17}H_{29}N_7O_5$

MW 365.2175 and 411.2230

accurate mass	ion formula	exact mass	err (ppm)
366.2245	$C_{16}H_{28}N_7O_3$	366.2248	0.9
412.2300	$C_{17}H_{30}N_7O_5$	412.2303	0.6

2. Figures

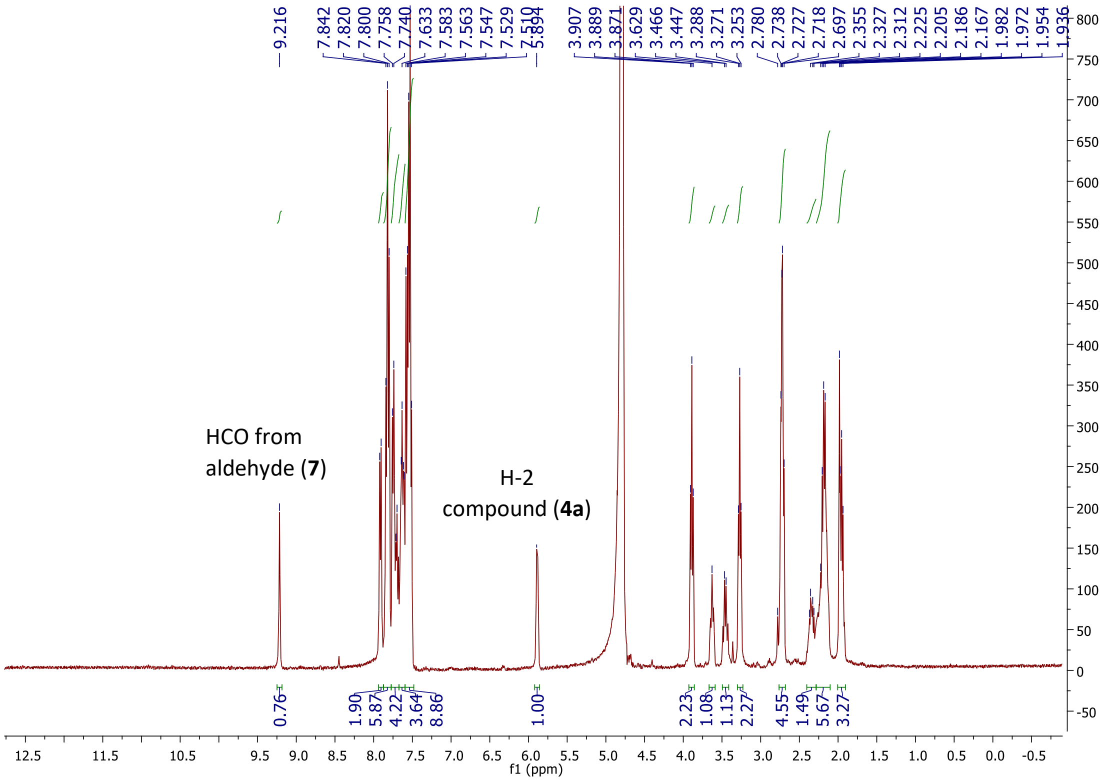

Figure S1. ^{1}H-NMR of the mixture of compounds **4a**, **6a** and **7**

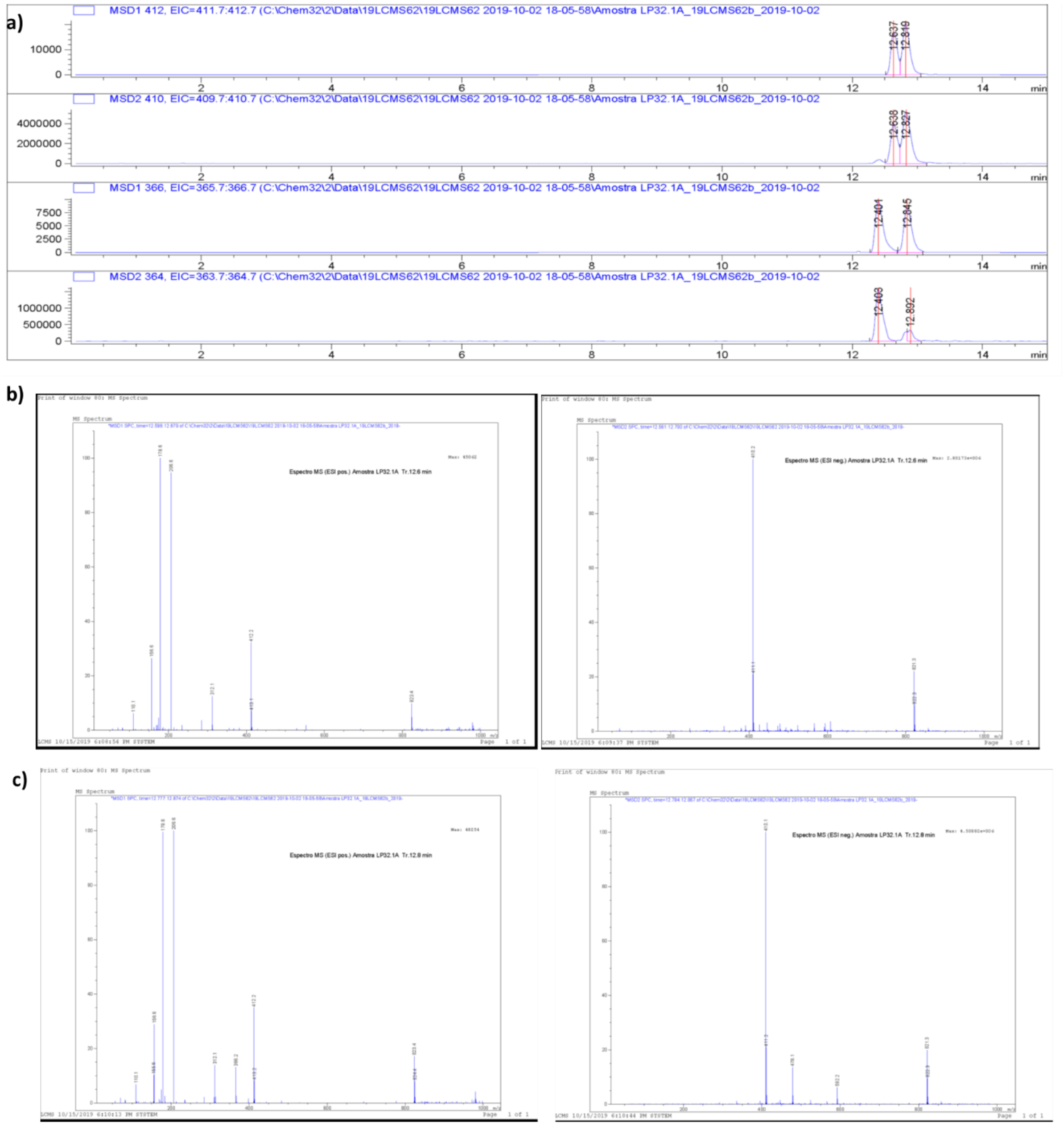

Figure S2. a) HPLC-ESI-MS chromatogram of reaction mixture to obtain compound **4h**, where the presence of two pairs of the diastereomers can be observed at 12.6 and 12.8 min; b) and c) extracted mass spectra of compound **4h** at 12.6 and 12.8 respectively (in positive *m*/*z* 366 and negative mode *m*/*z* 364)

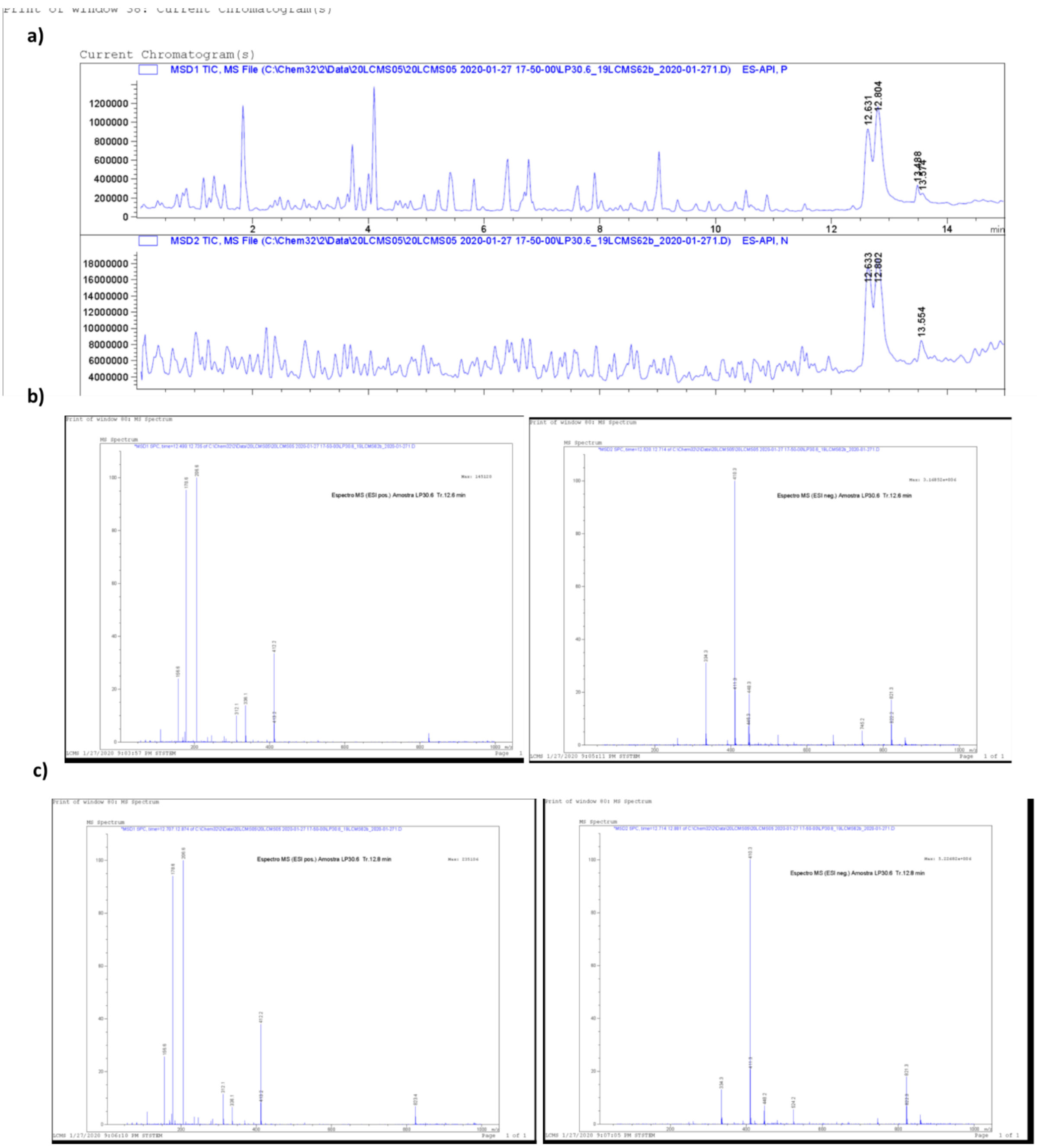

Figure S3. a) HPLC-ESI-MS chromatogram of compound **3h**, where the presence of two pairs of the diastereomers can be observed at 12.6 and 12.8 min; b) and c) extracted mass spectra of compound **3h** at 12.6 and 12.8 respectively (in positive *m/z* 412 and negative mode *m/z* 410)

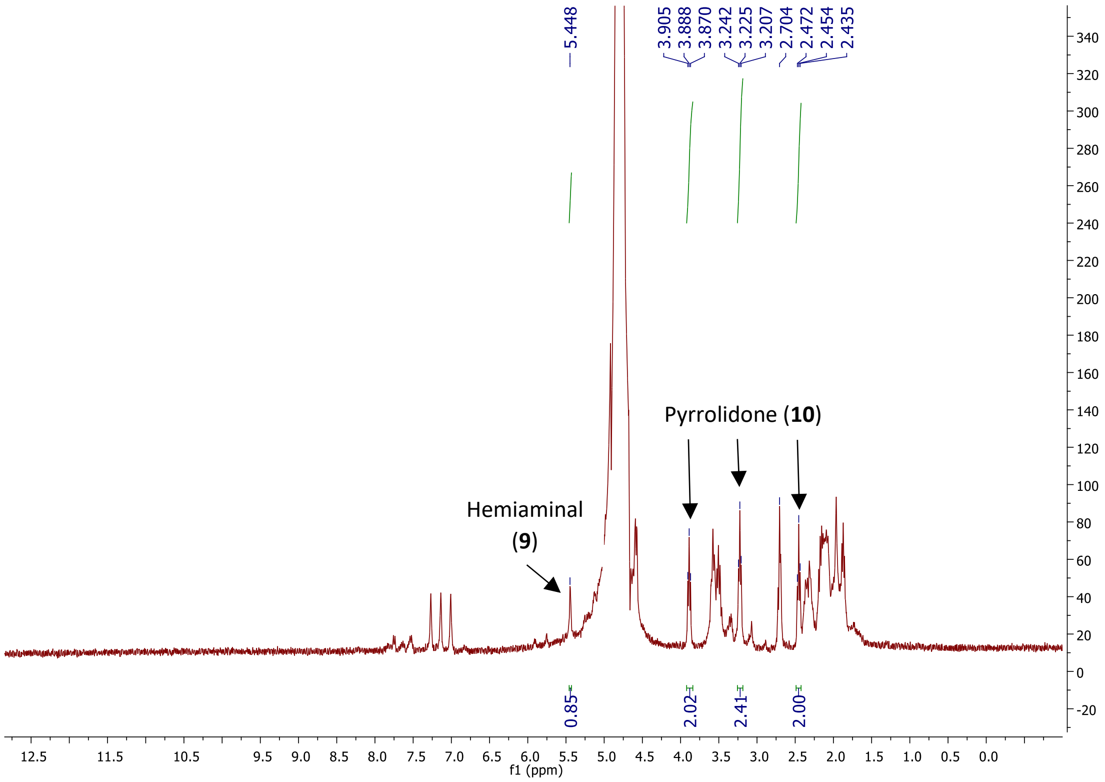

Figure S4. ^{1}H NMR spectrum of the crude mixture where the presence of hemiaminal (**9**) and pyrrolidone (**10**) were observed (signals between 7.2 and 7.0 ppm are due to the presence of oxidant). It is worth to mention that altough the reaction was performed in the presence of benzamide, the unreacted portion of the amide was removed during the work-up ($CHCl_3$ extractions).

a)

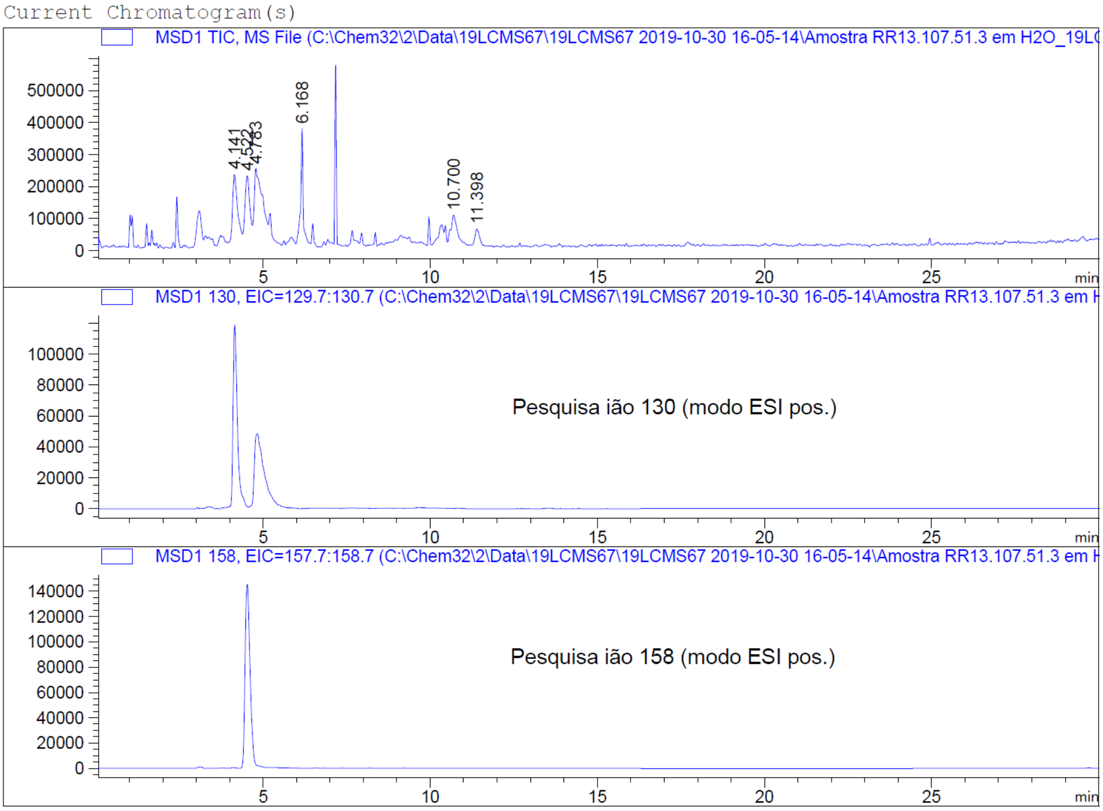

Current Chromatogram(s)
MSD1 TIC, MS File (C:\Chem32\2\Data\19LCMS67\19LCMS67 2019-10-30 16-05-14\Amostra RR13.107.51.3 em H2O_19L
6.168
4.141
4.522
4.783
10.700
11.398
MSD1 130, EIC=129.7:130.7 (C:\Chem32\2\Data\19LCMS67\19LCMS67 2019-10-30 16-05-14\Amostra RR13.107.51.3 em H
Pesquisa ião 130 (modo ESI pos.)
MSD1 158, EIC=157.7:158.7 (C:\Chem32\2\Data\19LCMS67\19LCMS67 2019-10-30 16-05-14\Amostra RR13.107.51.3 em H
Pesquisa ião 158 (modo ESI pos.)
min

b)

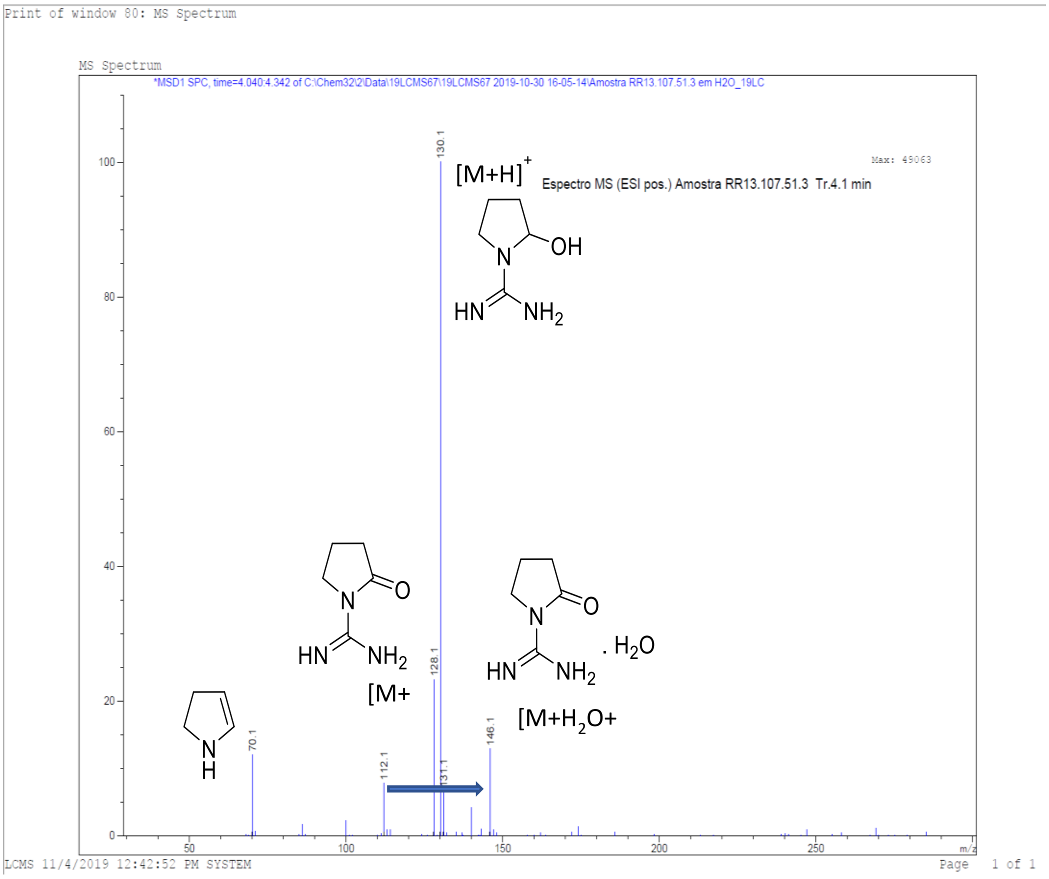

Print of window 80: MS Spectrum
MS Spectrum
Espectro MS (ESI pos.) Amostra RR13.107.51.3 Tr.4.1 min
[M+H]+
OH
N
HN
NH2
O
[M+
. H2O
[M+H2O+
130.1
128.1
146.1
112.1
70.1
131.1
LCMS 11/4/2019 12:42:52 PM SYSTEM
Page 1 of 1

c)

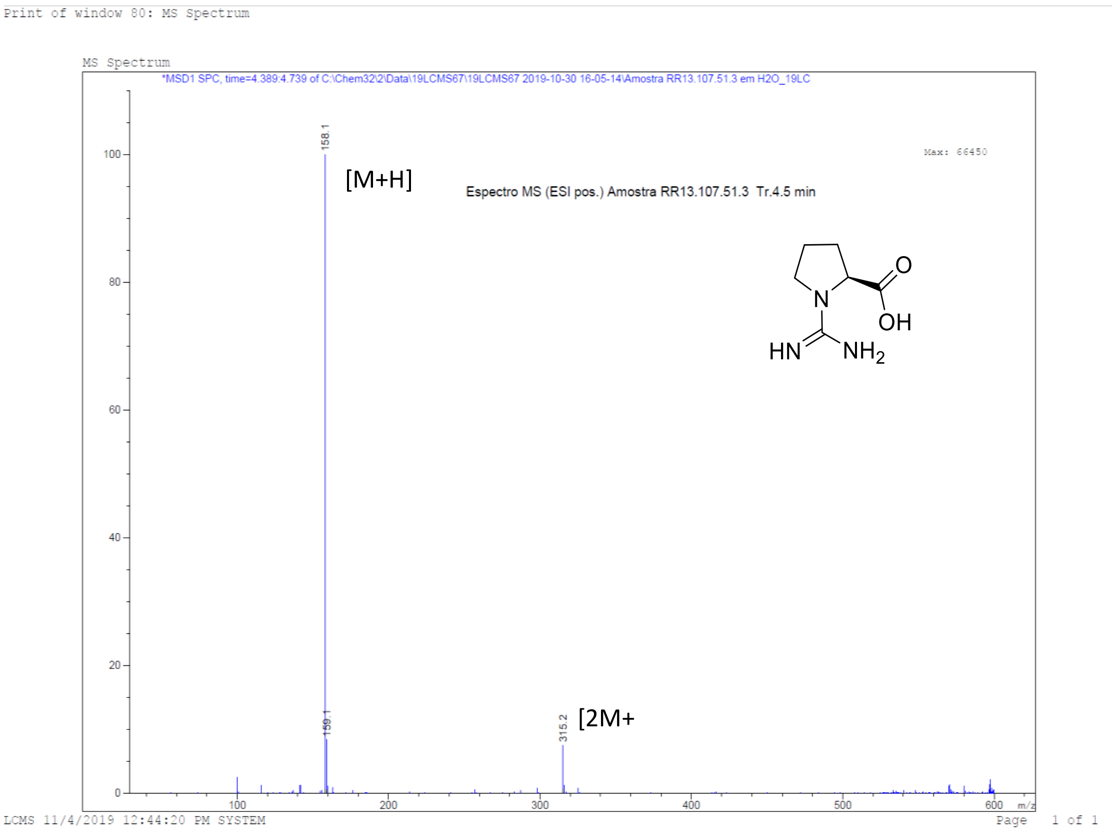
Print of window 80: MS Spectrum
MS Spectrum
*MSD1 SPC, time=4.389:4.739 of C:\Chem32\2\Data\19LCMS67\19LCMS67 2019-10-30 16-05-14\Amostra RR13.107.51.3 em H2O_19LC
Max: 66450
Espectro MS (ESI pos.) Amostra RR13.107.51.3 Tr.4.5 min
158.1
[M+H]
159.1
315.2
[2M+
N
O
OH
HN
NH2
m/z
LCMS 11/4/2019 12:44:20 PM SYSTEM
Page 1 of 1

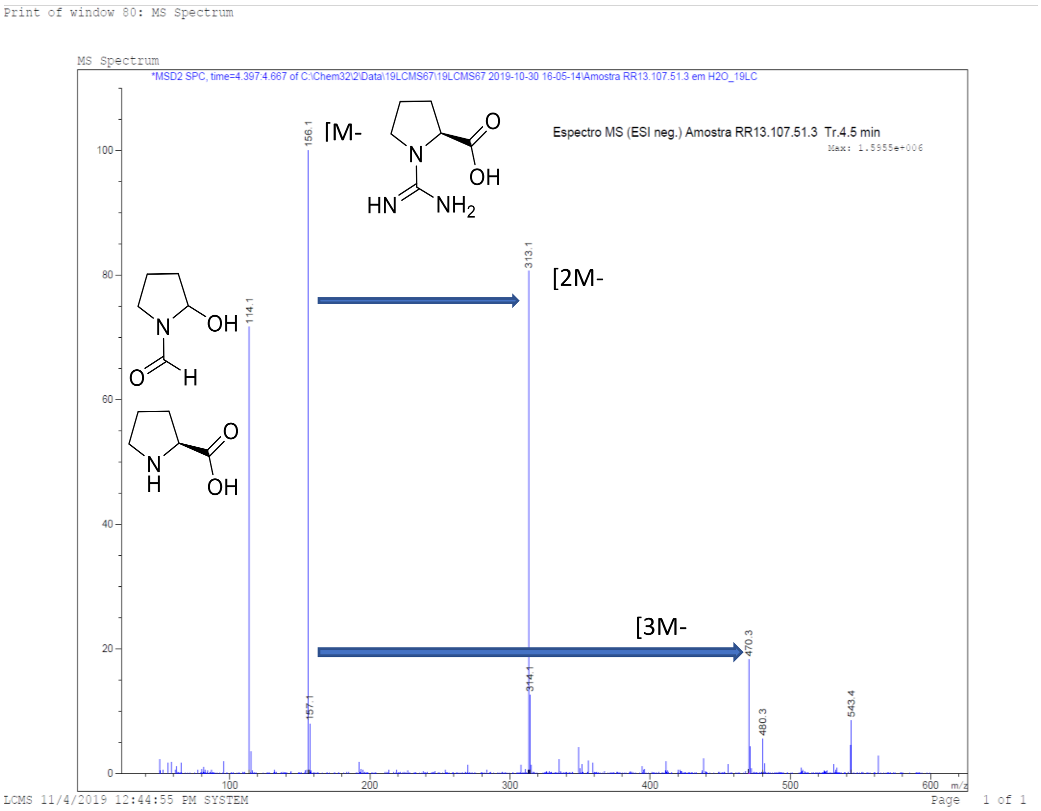
Print of window 80: MS Spectrum
MS Spectrum
*MSD2 SPC, time=4.397:4.667 of C:\Chem32\2\Data\19LCMS67\19LCMS67 2019-10-30 16-05-14\Amostra RR13.107.51.3 em H2O_19LC
Espectro MS (ESI neg.) Amostra RR13.107.51.3 Tr.4.5 min
Max: 1.5555e+006
156.1
[M-
313.1
[2M-
114.1
314.1
157.1
[3M-
470.3
480.3
543.4
N
OH
O
H
N
H
m/z
LCMS 11/4/2019 12:44:55 PM SYSTEM
Page 1 of 1

d)

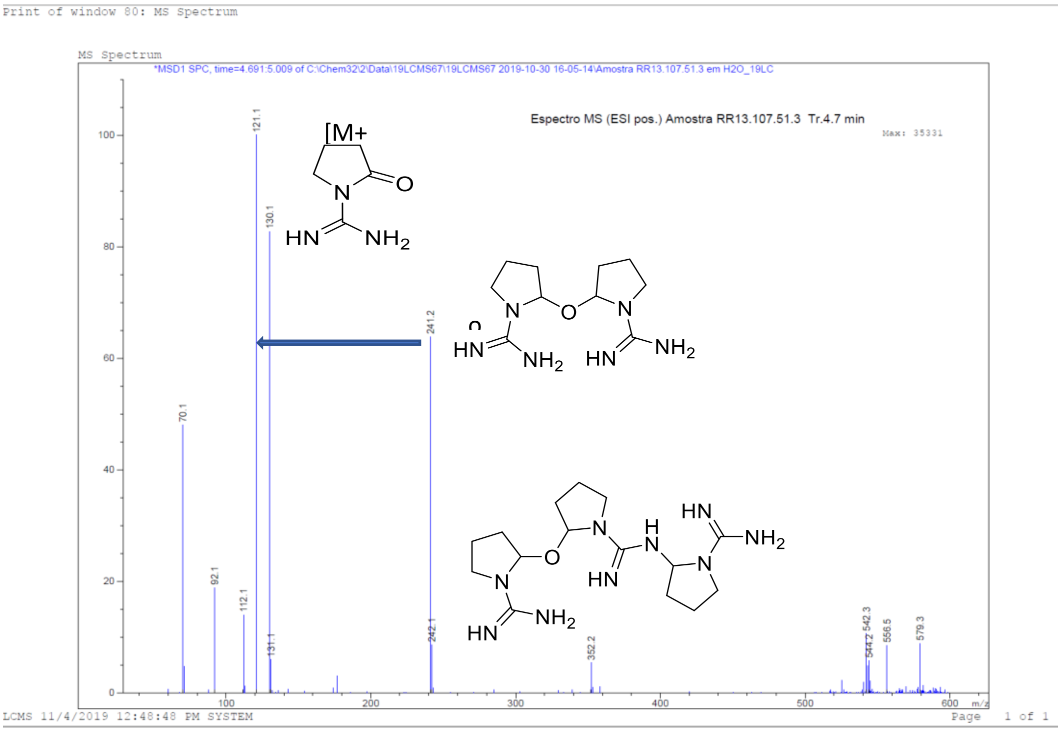

Figure S5 – a) HPLC-ESI-MS spectra of the decarboxylation of (**2**) in the absence of nucleophile; ion search for *m/z* 130 $[M+H]^+$ compound **9** and *m/z* 158 $[M+H]^+$ compound **2**; b) extracted spectra at the RT 4.1 min; c) extracted spectra at the RT 4.5 min in positive and negative mode; d) extracted spectra at the RT 4.7 min.
